# Lignin Biodegradation by a Cytochrome P450 Enzyme: A Computational Study into Syringol Activation by GcoA

**DOI:** 10.1002/chem.202002203

**Published:** 2020-09-16

**Authors:** Hafiz Saqib Ali, Richard H. Henchman, Sam P. de Visser

**Affiliations:** ^1^ Manchester Institute of Biotechnology The University of Manchester 131 Princess Street Manchester M1 7DN United Kingdom; ^2^ Department of Chemistry The University of Manchester Oxford Road Manchester M13 9PL United Kingdom; ^3^ Department of Chemical Engineering and Analytical Science The University of Manchester Oxford Road Manchester M13 9PL United Kingdom

**Keywords:** cyclization, density functional calculations, enzyme mechanisms, hydroxylation, reaction mechanisms

## Abstract

A recently characterized cytochrome P450 isozyme GcoA activates lignin components through a selective *O*‐demethylation or alternatively an acetal formation reaction. These are important reactions in biotechnology and, because lignin is readily available; it being the main component in plant cell walls. In this work we present a density functional theory study on a large active site model of GcoA to investigate syringol activation by an iron(IV)‐oxo heme cation radical oxidant (Compound I) leading to hemiacetal and acetal products. Several substrate‐binding positions were tested and full energy landscapes calculated. The study shows that substrate positioning determines the product distributions. Thus, with the phenol group pointing away from the heme, an *O*‐demethylation is predicted, whereas an initial hydrogen‐atom abstraction of the weak phenolic O‐H group would trigger a pathway leading to ring‐closure to form acetal products. Predictions on how to engineer P450 GcoA to get more selective product distributions are given.

## Graphical abstract

**Using an unnatural natural product**: Density functional theory calculations on a large cluster model of cytochrome P450 GcoA show that the enzyme can bind and activate lignin monomer units and convert them into hemiacetal and acetal products. Suggestions on how to bioengineer the enzyme to improve the selectivity are given.

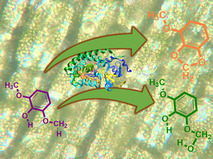

## Introduction

Lignin is a complex biopolymer that makes up the cell walls and tissues in plants as well as in some fungi. It is built up from mainly aromatic and phenolic residues bridged by ether and C−C bonds and has a highly branched structure that gives it its chemical and physical strength and biological properties. Several enzymes in nature can biodegrade lignin or parts thereof, including the lignin peroxidases, which contain a heme active site and utilize H_2_O_2_ as an oxidant.^1^ Currently, the agricultural and industrial sectors generate substantial amounts of lignocellulose, much of which currently goes to waste. However, lignocellulose has the potential to be converted into valuable materials or used as an energy source for liquid fuels. Therefore, ongoing studies to find biotechnological applications of lignin degrading enzymes are being conducted to convert lignin into small aromatic compounds or drugs.^2^

**Scheme 1:**
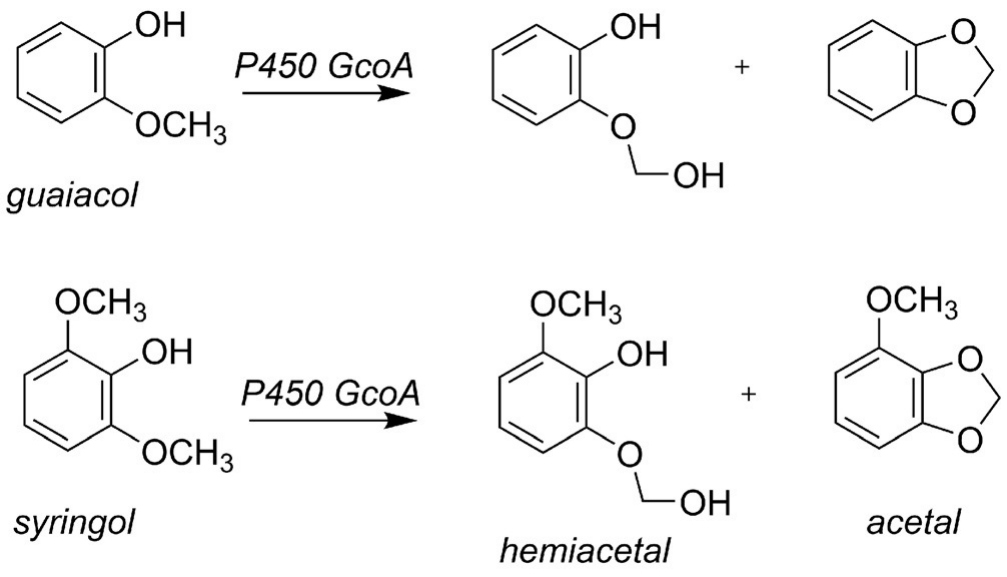
Possible reaction products of guaiacol and syringol activation by P450 GcoA.

Recently, it was found that the cytochromes P450 can also participate in lignin degradation pathways.^3^ These P450 enzymes are heme monoxygenases that utilize molecular oxygen, often as a means to hydroxylate aromatic or aliphatic substrates, although dealkylation reactions have also been reported.^4^ In particular, the P450 isozyme CYP255A (GcoA) was found to demethylate aromatic compounds such as those originating from lignin components, including guaiacol and various alkoxybenzoates.^5^ The work showed that engineered GcoA isozymes with enlarged substrate binding pockets, for example, through replacement of Phe_169_ by Ala, enhanced the reactivity with these substrates. Furthermore, studies with a variety of *O*‐methoxy‐aromatic compounds measured product distributions as well as substrate binding affinities and constants.^6^ A combined experimental and computational study looked into the mechanisms and possibilities of guaiacol activation by GcoA. Two pathways were considered, namely *O*‐demethylation proposed to start with methoxy hydroxylation to form hemiacetal and ring‐closure to form acetal (Scheme 1), whereby the former is expected to release formaldehyde to give catechol. A minimal density functional theory (DFT) cluster model was studied that did not consider the substrate‐binding pocket, but nevertheless gave insights into possible reaction pathways. Recent computational studies by us showed that the second coordination sphere has important functions in substrate and oxidant positioning and hence affects regio‐ and chemoselectivities of enzymatic reactions.^7^ Since, the substrate‐binding pocket in GcoA is tight, with various π‐stacking interactions, we felt a more advanced computational study that takes the effect of the protein into consideration would give a better model and more insight into the details of the reaction mechanism of GcoA enzymes, its substrate range and selectivity.

Moreover, acetal‐type structures and particularly cyclic ones are common in biomaterials, including corticosteroids and drug molecules like paroxetine. Hence, an enzyme that could synthesize cyclic acetal‐bound structures selectively would be useful in biotechnology. Therefore, we studied the reaction mechanism of the lignin fragment syringol (2,6‐dimethoxyphenol) activated by GcoA using a large active‐site cluster model of GcoA that includes much of the substrate‐binding pocket.

The *O*‐dealkylation of substrates by the P450s has been observed for various isozymes and, for instance, is part of the biodegradation and metabolism of drug molecules in the liver.^8^ Computational studies established a mechanism that starts with hydroxylation of the methyl group to form a hemiacetal‐like intermediate, which, in solution, upon addition of protons, releases formaldehyde to complete the *O*‐demethylation process.^9^ As such, the *O*‐demethylation reaction shows similarities with aliphatic hydroxylation by P450 enzymes that generally proceeds via a stepwise mechanism with an initial hydrogen‐atom abstraction by Compound I (CpdI; iron(IV)‐oxo heme cation radical intermediate) to form an iron(IV)‐hydroxo complex that rebounds its OH group to the substrate to form alcohol products.^10^ Experimental support for this hypothesis came from kinetic isotope effect (KIE) studies that established a large change in the rate constant when the transferring hydrogen atom was replaced with a deuterium atom.^11^

To gain insight into the lignin biodegradation pathways by P450 isozymes, we investigated the mechanism of syringol activation by a large GcoA model structure. In particular, we focused the work on the bifurcation pathways leading to *O*‐methoxy hydroxylation and acetal formation using two substrate‐binding orientations. The work shows that the protein environment is important: it sets up substrate approach, guides the reaction in a certain direction and leads to different product distributions with the different substrate orientations. As the phenol O−H bond is the weakest bond in the substrate, substrate activation preferentially takes place there, but is only possible with a substrate‐bound orientation that points the phenol group in the direction of the heme. Overall competing pathways to both products were identified and analyzed.

## Results

**Scheme 2:**
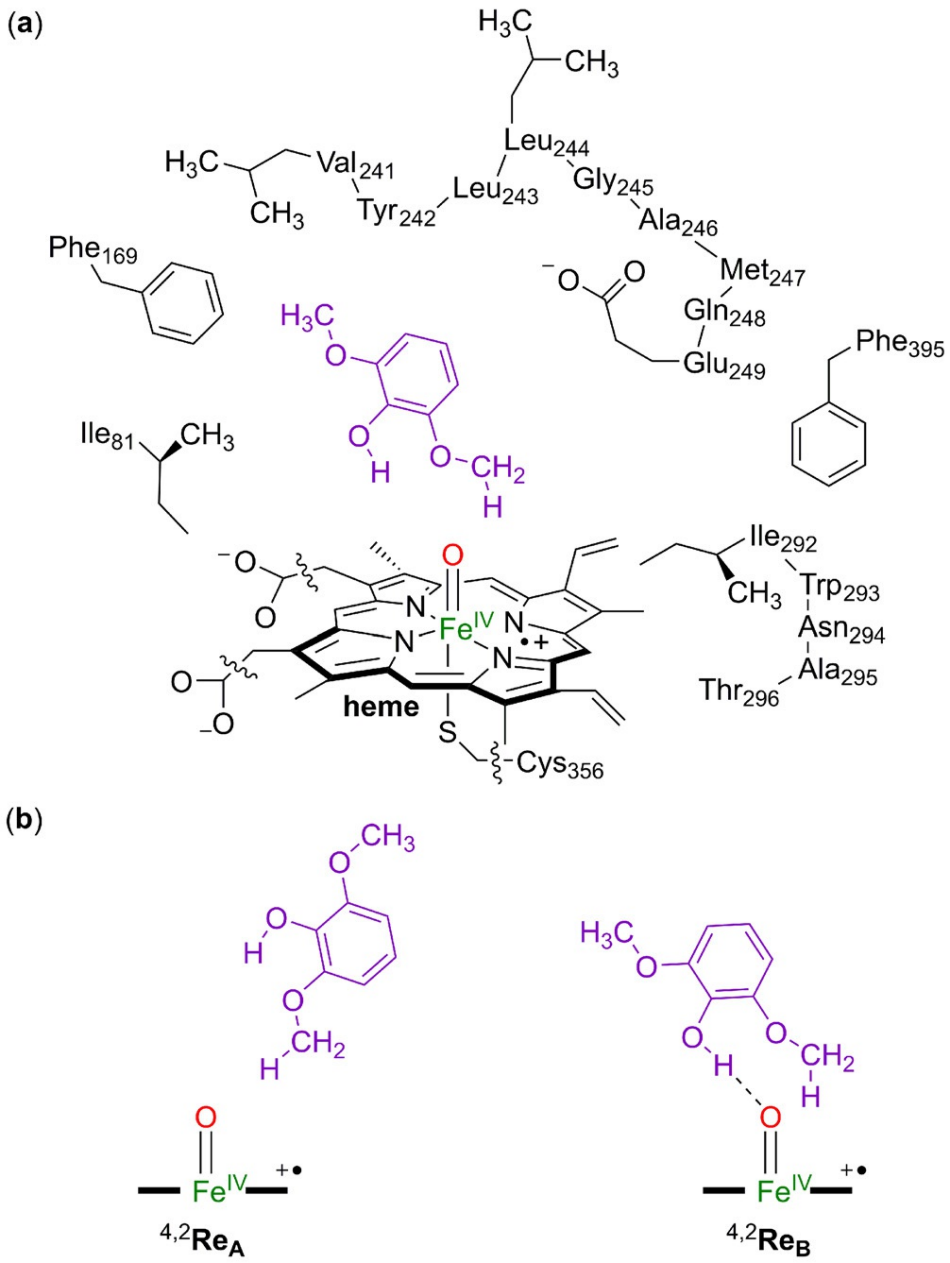
a) DFT cluster model studied in this work. Wiggly lines identify where covalent bonds were cut. b) Substrate orientations A and B.

Focusing on lignin biodegradation by P450 isozymes, we created a large active‐site cluster model of GcoA with syringol bound and studied substrate activation. Our model set‐up follows previously reported procedures from our group,^12^ that start from a deposited crystal structure from the protein databank (pdb),^13^ and a detailed analysis of the co‐factor and substrate environment. Based on key local environmental interactions from charged residues and hydrogen bonding and stereochemical influences, we created an active site cluster model of 302 atoms as shown in Scheme 2. The 5OMU protein databank file^6^ was used for the model as it is a P450 monomer structure of GcoA with syringol bound. The residues included in our model are highlighted in Scheme 2. We took the heme and kept all side chains except the propionate groups, which were replaced by methyl. The axial cysteinate of the heme (Cys_356_) was included as methylmercaptate and iron(III)‐heme was replaced with iron(IV)‐oxo heme cation radical, that is, Compound I (Cpd I). The substrate‐binding pocket was described through the residues Ile_81_ (as butane), Phe_169_ and Phe_395_ (as ethylbenzene). In addition, two elaborate protein chains were included in the model; namely, the chain Val_241_‐Tyr_242_‐Leu_243_‐Leu_244_‐Gly_245_‐Ala_246_‐Met_247_‐Gln_248_‐Glu_249_ and Ile_292_‐Trp_293_‐Asn_294_‐Ala_295_‐Thr_296_. The amino acid side chains pointing away from the substrate binding pocket were replaced with Gly; namely, those of Tyr_242_, Leu_243_, Met_247_, Trp_293_ and Asn_294_. The complete model was calculated in the doublet and quartet spin states. We decided to explore two different binding conformations of the substrate: model **A**, with one of the methoxy groups pointing toward CpdI, and model **B**, which has both the phenol and one of the methoxy groups in close proximity to CpdI (bottom of Scheme 2). These structures were manually created and are labelled as **Re_A_** and **Re_B_**, respectively.

**Figure 1:**
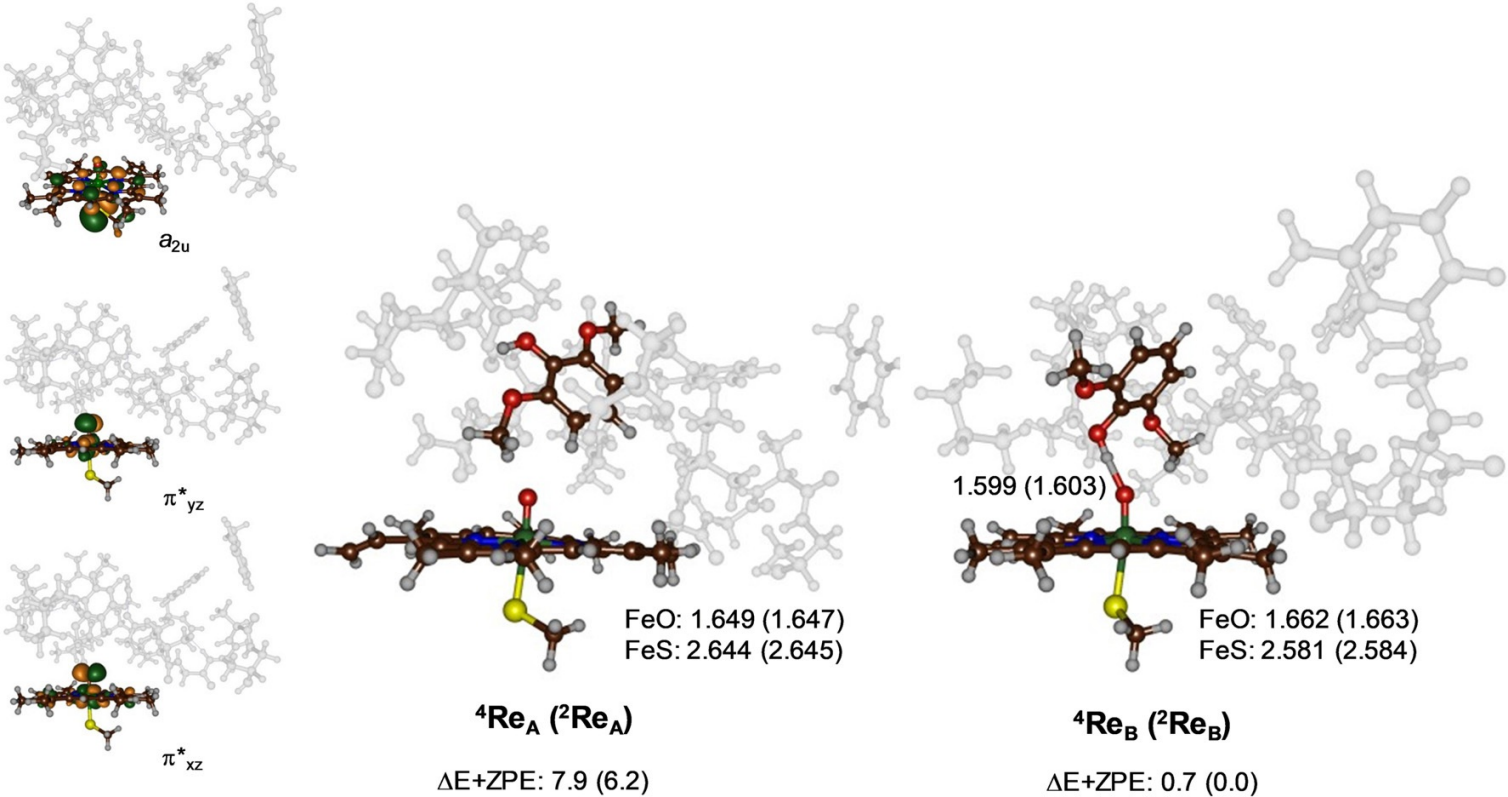
UB3LYP/BS1 optimized geometries of ^4,2^ **Re_A_** and ^4,2^ **Re_B_** with bond lengths in angstroms. Singly occupied orbitals shown on the left‐hand‐side for ^2^ **Re_A_** as an example. Relative energies (kcal mol^−1^) are UB3LYP/BS2//UB3LYP/BS1 values with zero‐point energy (ZPE) included.

DFT optimized geometries of the reactant complexes **Re_A_** and **Re_B_** in the doublet and quartet spin states are given in Figure 1. Both structures have close‐lying doublet and quartet spin state configurations with three unpaired electrons in the orbitals labelled as π*_xz_, π*_yz_ and a_2u_. Thus, the metal 3d‐orbitals interact with orbitals on the ligands and give the following five valence orbitals: *δ*_*x*2‐*y*2_, π*_xz_, π*_yz_, σ*_z2_ and σ*_xy_, whereby the *z*‐axis is defined along the S‐Fe‐O axis and the *xy*‐plane is in the porphyrin plane with both axes through the Fe−N bonds. The two σ* orbitals are virtual in Cpd I, whereas the *δ*_*x*2‐*y*2_ is nonbonding and doubly occupied. The singly occupied molecular orbitals of the CpdI reactant structures are shown on the left hand side of Figure 1 and represent the antibonding interactions of the metal with the oxo group (π*_xz_ and π*_yz_) and a mixed porphyrin‐axial ligand orbital labelled a_2u_.^13^ In the quartet spin state these three orbitals are ferromagnetically coupled, while in the doublet spin state the two π* orbitals are antiferromagnetically coupled to the a_2u_ electron.

As before,^14^ the doublet and quartet spin states of CpdI are close in energy, as can be seen from the pairs of energies for ^4,2^ **Re_A_** and ^4,2^ **Re_B_**. The structures **Re_B_** are the lowest in energy, probably due to the additional hydrogen bonding of the phenol group of the substrate with the oxo group of CpdI that gives these structures extra stability. Therefore, reactant configuration **Re_B_** has the substrate the strongest bound and hence represents the more favourable binding orientation.

Geometrically, there are differences between the reactant complexes **Re_A_** and **Re_B_**, mainly due to the hydrogen bond of the phenol group of the substrate to the oxo group in **Re_B_**. Thus, in **Re_A_** the Fe−O distance is short (1.649 and 1.647 Å for the quartet and doublet spin states), while they are elongated to 1.662/1.663 Å for ^4^ **Re_B_**/^2^ **Re_B_** as a result of the hydrogen‐bond interaction with the phenol group at 1.60 Å. At the same time, the Fe−S bond has shortened from about 2.64 Å in **Re_A_** to 2.58 Å in **Re_B_**. Overall, the optimized geometries and electronic configuration matches previous studies well on CpdI models with either DFT cluster models or QM/MM.^14^, ^15^

**Scheme 3:**
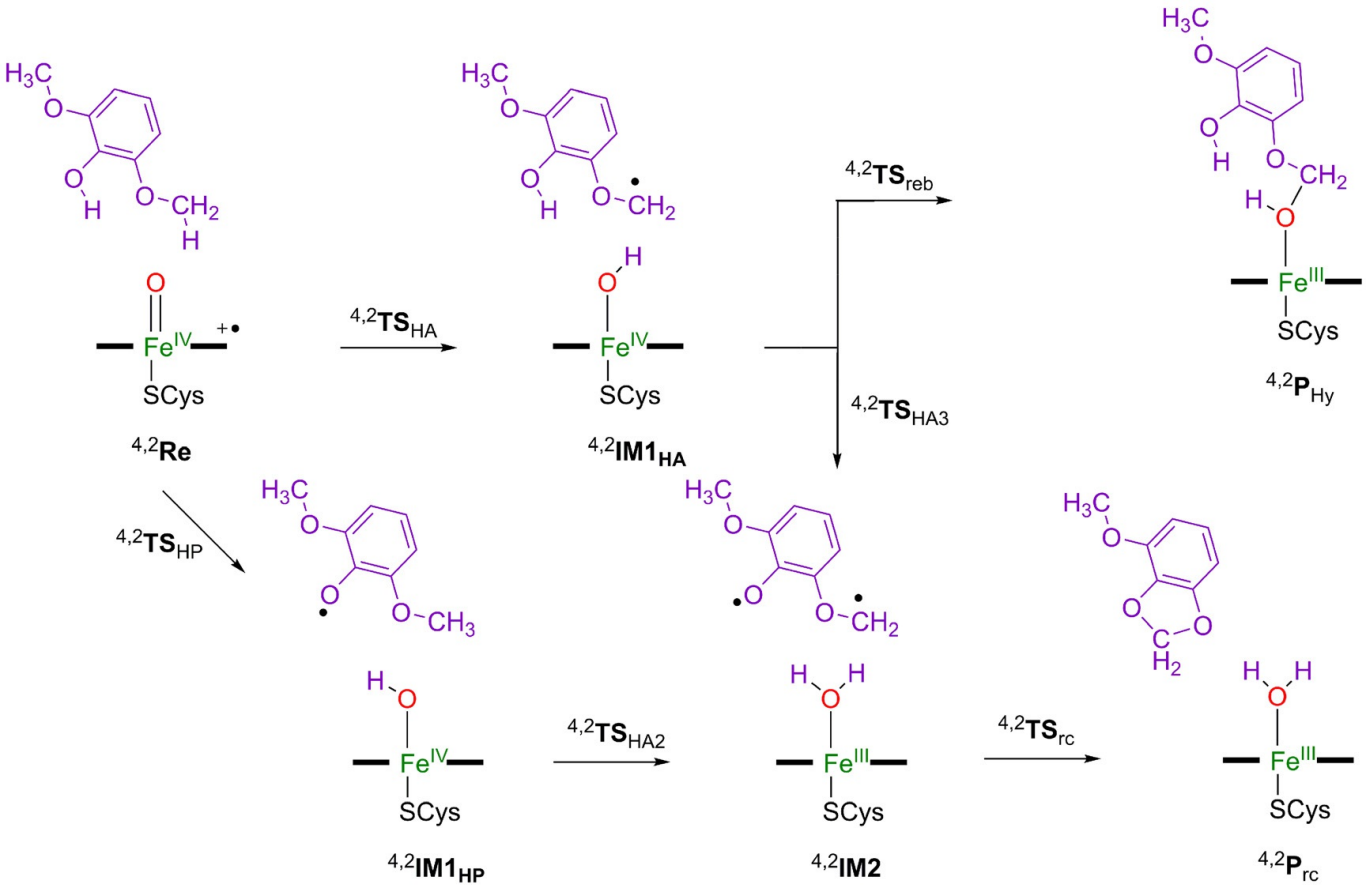
Reaction mechanism with definition of individual structures for syringol activation by GcoA.

Next we calculated the activation of syringol by CpdI using models **Re_A_** and **Re_B_** as starting points, whereby we give the substrate binding orientation with **A** or **B** as a subscript after the label. Details of the pathways explored with definition of the structures are given in Scheme 3. Firstly, we tested hydroxylation of the methoxy group of syringol for models **A** and **B** and calculated the hydrogen‐atom abstraction transition state (**TS_HA_**) from the methoxy C−H bond by CpdI that leads to an iron‐hydroxo complex and substrate radical (**IM1_HA_**). Radical rebound via **TS_reb_** gives the hemiacetal product complex (**P_Hy_**). Due to substrate positioning, these pathways are possible for both model **A** and model **B**. However, for substrate positioning **B**, we also explored alternative pathways that involve the phenol group of the substrate. Thus, for the substrate bound in orientation **B**, we investigated hydrogen‐atom abstraction from the phenol group via transition‐state **TS_HP,B_** to form the alternative radical intermediate **IM1_HP,B_**. In substrate orientation **A**, the phenol group points away from CpdI and hence O‐H hydrogen‐atom abstraction is not feasible in this orientation. From the intermediate **IM1**_HP,B_, a second hydrogen‐atom abstraction from the methoxy group of the substrate via transition‐state **TS**_HA2_ was tested to give an iron(III)‐water complex and a biradical on the substrate (**IM2**_HP,B_). Of course, **IM2**_HP,B_ can also be formed from **IM2**_HA,B_ by hydrogen‐atom abstraction from the phenol group in **IM1**_HA,B_ via transition state **TS**_HA3,B_. The biradical via a ring‐closure transition state (**TS**_rc,B_) leads to the acetal product complex (**P**_rc,B_).

**Figure 2:**
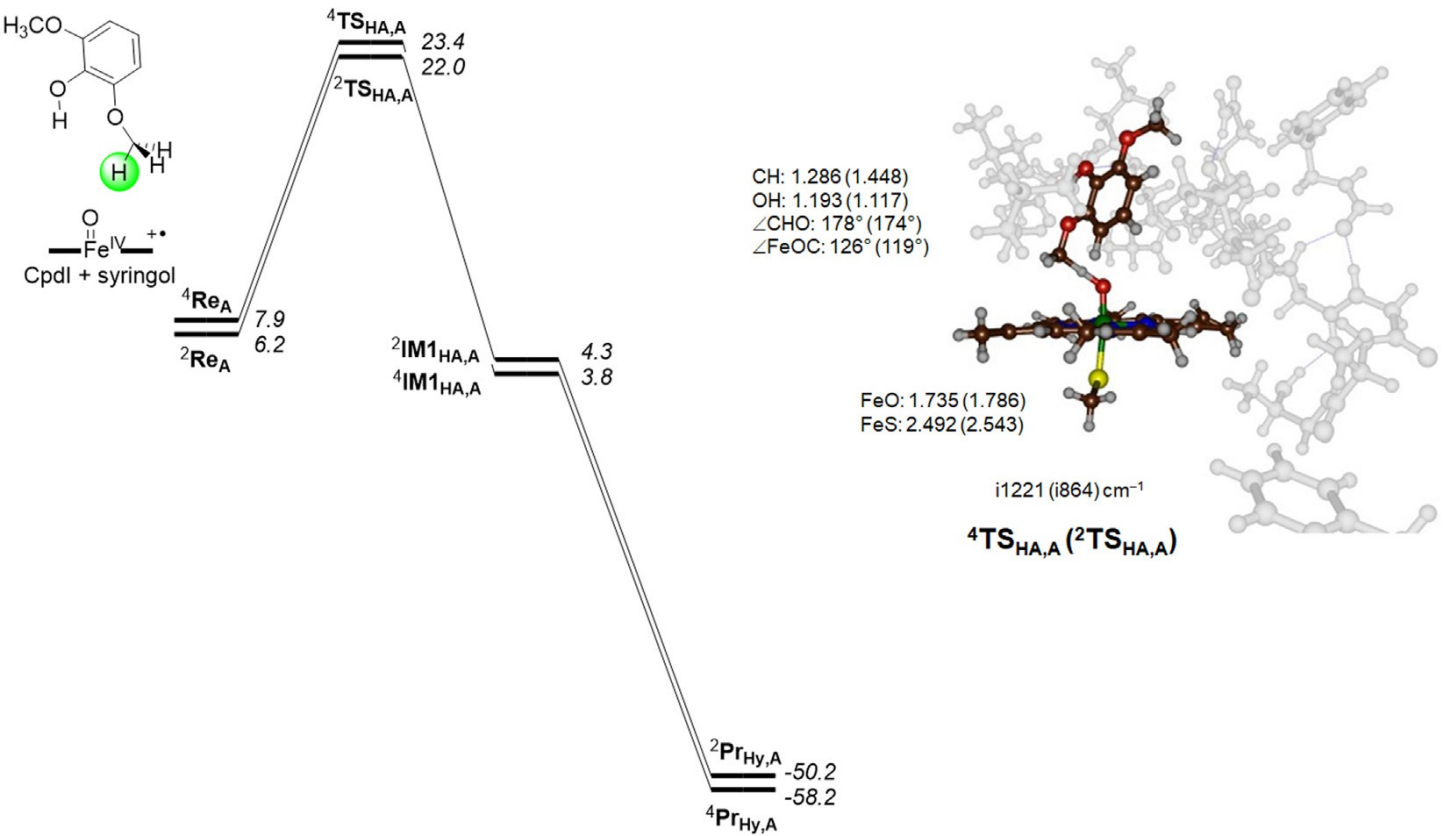
UB3LYP/BS2//UB3LYP/BS1 calculated potential energy surface for syringol activation by CpdI model **A** of GcoA. Energies contain ZPE and are given in kcal mol^−1^ relative to ^2^ **Re_B_**. Optimized geometries of the transition states give bond lengths in angstroms, angles in degrees and the imaginary frequency in cm^−1^.

We first consider the substrate activation using model **A**, where only aliphatic hydroxylation of the methoxy group is possible. The calculated potential energy landscape for hydroxylation of the methoxy group of syringol by a CpdI model **A** of GcoA is given in Figure 2. The hydrogen‐atom abstraction barriers (^4,2^ **TS**_HA,A_) are relatively high in energy: 22.0 and 23.4 kcal mol^−1^ in the doublet and quartet spin states, respectively. However, these values are relative to the more stable reactant conformation ^2^ **Re**_B_, although relative to the reactant in the same configuration, **Re**_A_, they are still 15.8 and 17.2 kcal mol^−1^ in energy. The optimized geometries of the transition states are given on the right hand side of Figure 2. Both structures have a characteristic, almost linear O−H−C angle ranging from 174°–178°, which is typical for hydrogen atom abstraction transition states.^16^ The transition states are product‐like, with larger C−H than O−H distances. Generally, product‐like transition states correspond to higher reaction barriers than earlier transition states,16a as confirmed from the relative energies.

The aliphatic hydrogen‐atom abstraction transition states ^4,2^ **TS_HA,A_** are characterized by a large imaginary frequency *i*1221 cm^−1^ in the quartet spin state and *i*864 cm^−1^ in the doublet spin state. The imaginary frequencies in the transition states represent the C−H−O stretch vibration along the reaction coordinate. The large values for the imaginary frequency are typical of hydrogen‐atom abstraction barriers and indicate a large amount of tunnelling and that the reaction likely will show a large kinetic isotope effect when the transferring hydrogen atom is replaced with deuterium.^17^

After the transition states, the system relaxes to a radical intermediate (^4,2^ **IM1**_HA,A_). On both spin‐state surfaces an electron transfer from the substrate into the a_2u_ orbital takes place to give an iron(IV)‐hydroxo(heme) and substrate radical, whereby the substrate has up‐spin radical in the quartet and down‐spin in the doublet. Both π*_xz_ and π*_yz_ orbitals remain singly occupied in the radical intermediates ^4,2^ **IM1**_HA,A_.

The radical intermediates in pathway **A**, that is, ^4,2^ **IM1**_HA,A_, are characterized as local minima on the potential energy surface, with real frequencies only. However, the radical rebound barriers for both spin states were found to be very low in energy (<1 kcal mol^−1^) and hence could not be characterized. Therefore, the radical intermediates will have a short lifetime and quickly collapse to form alcohol products. Indeed, the exothermicity from radical intermediates to products ^4,2^ **Pr**_Hy,A_ is very large. These short radical lifetimes of the intermediate complexes also make a possible ring‐closure to form the acetal products for this substrate binding orientation unlikely and, hence, the reaction will be highly selective in substrate‐binding position **A**. In previous work it was shown through valence bond rationalization that the doublet spin radical rebound barrier correlates with the ionization energy of the radical and the electron affinity of the iron(IV)‐hydroxo complex.16a, 17a, 18 In the quartet spin state the radical rebound in addition has a term for the electron excitation from the π*_xz_ to σ*_z2_ orbital. We calculated the ionization energy of the radical to be 166.4 kcal mol^−1^ and, with the reported electron affinity of the iron(IV)‐hydroxo species of 88.9 kcal mol^−1^,^19^ predict a negligible rebound barrier from valence bond principles and that consequently, the rebound will be fast.

**Figure 3:**
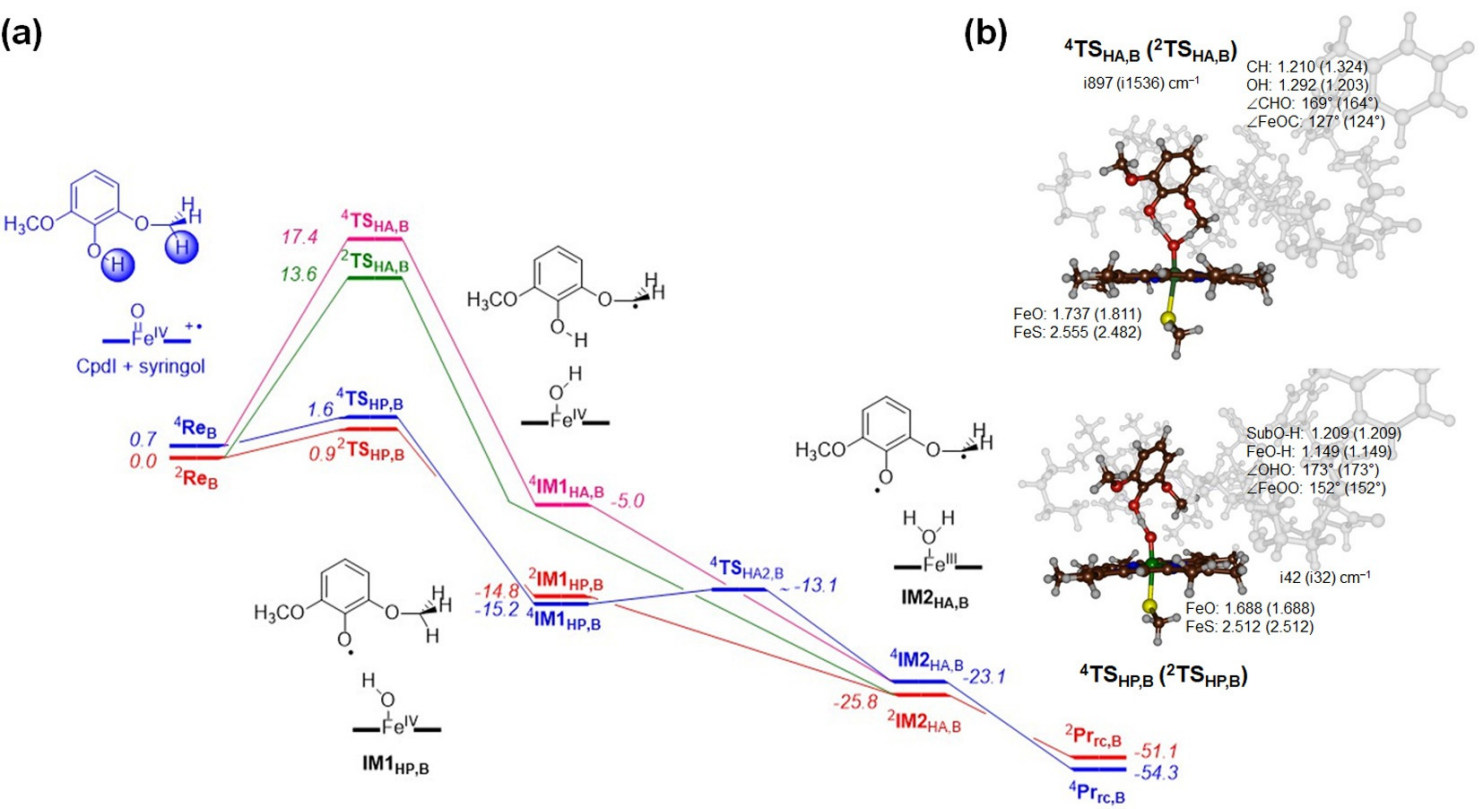
UB3LYP/BS2//UB3LYP/BS1 calculated potential energy surface for syringol activation by CpdI model **B** of GcoA. Energies contain ZPE and are given in kcal mol^−1^ relative to ^2^ **Re_B_**. Optimized geometries of the transition states give bond lengths in angstroms, angles in degrees and the imaginary frequency in cm^−1^.

Subsequently, the substrate activation pathways with substrate in binding position **B** were explored, and the results are presented in Figure 3. The lowest barriers were obtained for phenolic hydrogen‐atom abstraction, with a magnitude of Δ*E* ^≠^+ZPE=0.9 and 1.6 kcal mol^−1^ in the doublet and quartet spin states, respectively. Recent work of ours on the P450 isozyme responsible for the vancomycin biosynthesis in OxyB showed that two sequential phenolic hydrogen‐atom abstraction reactions can be performed by CpdI and CpdII (Compound II) to enable the aromatic cross‐linking of glycopeptide units.^20^ For the P450 OxyB system, the two hydrogen‐atom abstraction barriers were found to be very low in energy as the phenolic O−H bonds are very weak. The values of the hydrogen‐atom abstraction barriers in GcoA are also extremely low in energy, in line with the OxyB results. However, both of these sets of barriers are much lower in energy than those calculated previously for the abstraction from aliphatic C−H bonds.^16^ For instance, using the same computational methods as used here, a hydrogen‐atom abstraction barrier from the benzylic position of ethylbenzene gave a value of 12.6 kcal mol^−1^, whereas 14.5 kcal mol^−1^ was found for the C^5^−H bond cleavage in camphor.16a Our aliphatic hydrogen‐atom abstraction barriers from the methoxy group of syringol indeed have values of that size with ^2^ **TS_HA,B_** at 13.6 kcal mol^−1^ and ^4^ **TS_HA,B_** at 17.4 kcal mol^−1^. For pathway **A**, the barrier height with respect to **Re_A_** is similar, as expected because the same C−H bond is broken and the same electron transfer takes place. However, since the substrate‐bound complex **B** is more stable, its hydrogen‐atom abstraction barriers are lower in energy. Nevertheless, the ^2,4^ **TS_HA,B_** and ^2,4^ **TS_HA,A_** structures are strikingly different. Although the substrate binding position **A** has the substrate in an upright position, its transfer of a hydrogen atom takes place at an almost linear angle O−H−C of 178° and 174° for the quartet and doublet spin states, respectively. By contrast, in the ^4,2^ **TS_HA,B_** structures, the angles are slightly more bent (169° and 165°) because the hydrogen bond from the phenol group to the oxo gives the substrate approach less flexibility. These differences in orientation also affect the C−H and O−H distances in the transition states, as is seen in Figures 2 and 3.

After the aliphatic hydrogen‐atom abstraction in the quartet spin state, the system relaxes to a radical intermediate (^4^ **IM1**_HA,B_), which is similar to that seen for the structure in binding position **A**. However, due to additional hydrogen‐bonding interactions, ^4^ **IM1**_HA,B_ is much lower in energy than ^4^ **IM1**_HA,A_: −5.0 kcal mol^−1^ with respect to ^4^ **Re**_B_. Furthermore, the reaction is followed by an almost barrierless second hydrogen‐atom abstraction; namely, hydrogen‐atom abstraction from the phenol O−H group leads to ^4^ **IM2**_HA,B_ with large exothermicity. A subsequent, also barrierless ring‐closure step gives the acetal products. In addition to this pathway, we attempted to calculate the OH rebound from ^4^ **IM1**_HA,B_ to form the hemiacetal products. However, due to hydrogen‐bonding interactions between the phenol group and the iron‐hydroxo groups, the radical rebound is hampered. The constraint geometry scan for the radical rebound from ^4^ **IM1**_HA,B_ therefore gave a barrier of at least 13.7 kcal mol^−1^. Previously, in nonheme iron halogenases as well as in the P450 decarboxylase OleT and synthetic model complexes, we identified hydrogen bonds to an iron‐hydroxo intermediate that prevented radical rebound and guided the mechanism to a side reaction.18b, 21 Consequently, substrate positioning in GcoA enzymes is very important and determines the reaction mechanism, whereby substrate binding orientation **B** can lead to acetal products, while we do not see those products resulting from substrate binding position **A**. On the doublet spin‐state surface no radical intermediate (^2^ **IM1**_HA,B_) could be identified and its geometry optimization fell to ^2^ **IM2**_B_ directly. Similar to the high‐spin, this intermediate reverted to the acetal product in a barrierless fashion.

Finally, we tested phenol activation by our GcoA CpdI model. Phenolic hydrogen‐atom abstraction is possible in substrate binding position **B** and happens through a very small transition state with an imaginary frequency of only *i*42 (*i*32) cm^−1^ for ^4^ **TS_HP,B_** (^2^ **TS_HP,B_**), respectively. Analysis of the imaginary frequency gives a clear hydrogen‐transfer mode, although part of the substrate displaces as well. Similar to the aliphatic transition states, the O−H−O angle around the transferring hydrogen atom is almost linear: 173° in both spin states. The transition states are relatively central, with slightly shorter FeO−H distances than phenolic H−O distances.

After the phenolic hydrogen‐atom abstraction, the system relaxes to a radical intermediate (^4,2^ **IM1**_HP,B_). These structures are much lower in energy than reactants by 14.8 (15.2) kcal mol^−1^ in the doublet (quartet) spin states. As such, these radical intermediates will be quickly formed. Since, no OH rebound is possible after phenolic hydrogen‐atom abstraction, we explored a second hydrogen‐atom abstraction from the methoxy CH_3_ group. On the doublet spin state, the barrier (^2^ **TS**_HA2,B_) is negligible and the system transfers to the iron(III)‐water complex (^2^ **IM2**_HA,B_) with an exothermicity of more than 10 kcal mol^−1^. A geometry scan for the quartet spin pathway identified a small barrier (^4^ **TS**_HA2,B_) about 2.1 kcal mol^−1^ above ^4^ **IM1**_HP,B_ (see the Supporting Information, Figure S7). As this is a low‐barrier transition state that is located close to a local minimum, our TS search failed to converge and the optimization fell back to the intermediate. As discussed above, the iron(III)‐water complexes ^4,2^ **IM2**_HA,B_ quickly close the acetal ring to form the ^4,2^ **Pr**_rc,B_ products without much of a barrier.

Overall, the mechanism for substrate activation in binding position **B** shows that sequentially two hydrogen atoms are abstracted from the substrate, the first one from the phenol O−H group and second one from a methoxy C−H group. The first reaction barrier is rate‐determining, with the subsequent barriers being too small to be fully characterized. Therefore, the acetal formation will be a highly efficient and fast process and much faster than the substrate‐binding and product‐release steps in the protein. Consequently, the results on syringol activation by a large GcoA model shows that different products are predicted from substrate binding positions **A** and **B**.

## Discussion

**Scheme 4:**
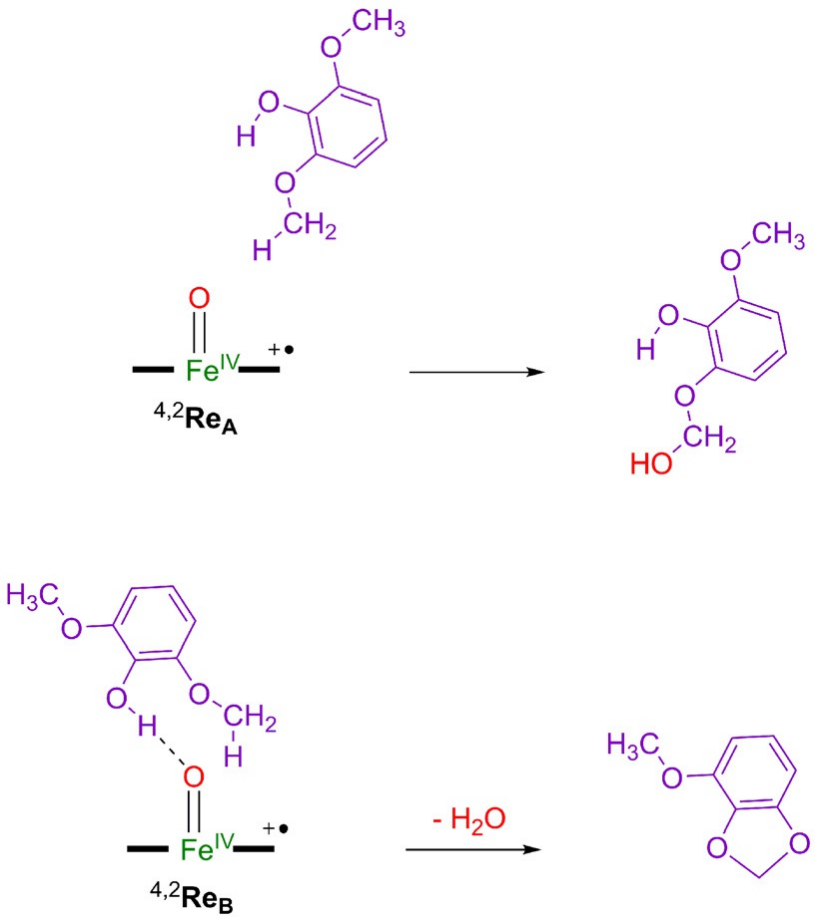
Products obtained for substrate activation by GcoA through substrate orientation **A** and **B**.

The work described here is focused on syringol activation by a lignin activating P450 isozyme; namely, GcoA. A large active site model of 302 atoms was considered that contains the heme active site and a large part of the substrate binding pocket with the substrate in two specific binding poses. The mechanism of substrate activation leading to hemiacetal and acetal products for the two binding poses was investigated. In substrate binding position **A** a rate‐determining hydrogen‐atom abstraction leads to methoxy hydroxylation efficiently, see Scheme 4. By contrast, in binding pose **B** the weak phenolic O−H bond points toward the heme and therefore can be abstracted by CpdI easily. In particular, the phenolic O−H group has a much lower barrier for hydrogen‐atom abstraction than the aliphatic C−H abstraction from the methoxy group. However, this hydrogen atom can be abstracted in a subsequent step and lead to ring‐closure to form acetal products. As such, the two substrate binding poses (Scheme 4) lead to different product distributions for syringol activation by GcoA. To understand the key factors that determine substrate activation, we analyzed the structures in more detail.

**Figure 4:**
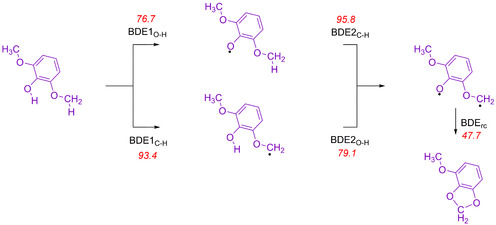
UB3LYP/6‐311++G** calculated bond dissociation and formation energies (kcal mol^−1^) including ZPE corrections in the substrate syringol.

Firstly, we calculated the various C−H and O−H bond dissociation energies (BDE1s) of syringol substrate (SubH) using Equation (1). The BDE1 values were estimated from the difference in energy of the individually calculated species in the reaction; that is, we calculated the substrate, a hydrogen atom and the substrate with one hydrogen atom removed from either the phenol or methoxy groups (Sub^.^). The reaction energy for Equation (1) was then evaluated for hydrogen‐atom abstraction from the phenol group of syringol (BDE1_O‐H_) and for hydrogen‐atom abstraction from the C−H group of the methoxy unit (BDE1_C‐H_), see Figure 4. At the UB3LYP/6‐311++G** level of theory, we find a BDE1_O−H_=76.7 kcal mol^−1^ and a BDE1_C−H_=93.4 kcal mol^−1^, see Figure 4. Therefore, the phenolic O−H bond is considerably weaker than the aliphatic C−H bond of the substrate and it should be easier to abstract the phenolic hydrogen atom than the methoxy hydrogen atom. Indeed, the potential energy landscape in Figure 3 for pathway **B** shows that the phenolic hydrogen‐atom abstraction has a much lower barrier than the one for aliphatic C−H abstraction, in line with the large differences in BDE values.

(1) $$\mathrm{Sub}\text{-}H\to \mathrm{Sub}{}^{•}+H{}^{•}+\mathrm{BDE}1{}_{\mathrm{Sub}\text{-}H}$$

For a small model complex of CpdI representing [Fe^IV^(O)(Por^+.^)SCH_3_], porphyrin without side chains, we calculated the BDE_CpdII_ from Equation (2) as the energy difference between [Fe^IV^(OH)(Por)(SCH_3_)] and CpdI and an isolated hydrogen atom and obtained a value of BDE_CpdII_=87.4 kcal mol^−1^. In previous work, a slightly smaller model with thiolate rather than SCH_3_^−^ as axial ligand was used, which gave a BDE_CpdII_=88.9 kcal mol^−1^ in the gas‐phase.^19^, ^22^ Therefore, the change in axial ligand from thiolate to methylmercaptate has little effect on the BDE_CpdII_ values. The energy differences in Figure 1 and 2 between the reactant complexes and **IM1**_HA_ and **IM1**_HP_ should be equal to the difference in energy between the C−H/O−H bonds broken and formed. The difference in energy between BDE1_O‐H_ and BDE_CpdII_ is −10.7 kcal mol^−1^, which is close in energy to the exothermicity to form **IM1**_HP,B_ from reactants. Hence, the potential energy landscape in Figure 3 for the hydrogen‐atom abstraction follows the strengths of the C−H and O−H bonds that are broken and formed.

**Figure 5:**
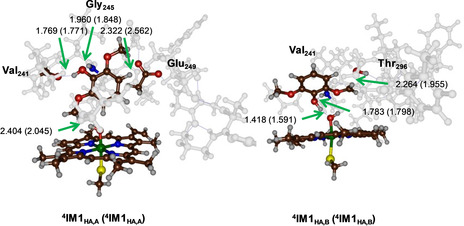
Optimized geometries of ^4,2^ **IM1**_HA,A_ and ^4,2^ **IM1**_HA,B_ as obtained at UB3LYP/BS1 with bond lengths in Ångstroms.

Similar to the phenol hydrogen atom abstraction pathway, we evaluated the difference in the bond strengths that are formed and broken for hydrogen atom abstraction from the methoxy group [Eq.(1)]. Thus, the difference in energy between BDE1_C‐H_ and BDE_CpdII_ is +6.0 kcal mol^−1^. The energy difference between ^2^ **Re**_A_ and ^2^ **IM1**_HA,A_ is −1.9 kcal mol^−1^ (Figure 2), while that between ^2^ **Re**_B_ and ^2^ **IM1**_HA,B_ is −5.7 kcal mol^−1^ (Figure 3). These driving forces are, therefore, somewhat lower in energy than what would have been predicted based on the difference in bond dissociation energy of the bonds that are broken and formed. To understand these differences better, we display in Figure 5 the optimized geometries of ^4,2^ **IM1**_HA,A_ and ^4, 2^ **IM1**_HA,B_. The **IM1**_HA,A_ radical intermediates have the substrate hydrogen bonded to the peptide carbonyl of Val_241_, the amide of Gly_245_, while the methoxy group hydrogen bonds to the carboxylate of Glu_249_. All of these interactions are well over 1.7 Å in length and, hence, there is only a small stabilization effect with respect to the thermodynamic bond energy differences due to hydrogen‐bonding interactions. By contrast, in the **IM1**_HA,B_ structures, the phenol OH group is close to the iron(IV)‐hydroxo, at a distance of 1.418 (1.591) Å in the quartet (doublet) spin state. This is a strong hydrogen‐bonding interaction that will stabilize these radical intermediates considerably. Indeed, the stabilization energy is much more exothermic than the difference in bond strength of the bond that is broken and formed implicates. The hydrogen‐bonding interactions of the protein and the iron‐hydroxo species, therefore, stabilize the radical intermediates and make the reaction more exothermic. Therefore, the first hydrogen‐atom abstraction barriers follow the thermodynamics of the individual hydrogen‐atom abstraction processes.

(2) $$\left[\mathrm{Fe}\left(\mathrm{Por}\right)\left(\mathrm{SCH}{}_{3}\right)\right]O-H\to \mathrm{CpdI}+H{}^{•}+\mathrm{BDE}{}_{\mathrm{CpdII}}$$

Subsequently, we calculated the phenolic O−H bond strength and the methoxy aliphatic C−H bond strength from the radicals as BDE2_O−H_ and BDE2_C−H_, also shown in Figure 4. Values of BDE2_O−H_=79.1 kcal mol^−1^ and BDE2_C‐H_=95.8 kcal mol^−1^ were calculated. This implies that the second hydrogen‐atom abstraction requires a similar energy to break as the first hydrogen‐atom abstraction. To predict the reaction energy for the second hydrogen‐atom abstraction step, we calculated the BDE2_water_ for the conversion of an iron(III)‐water(heme) complex into an iron(IV)‐hydroxo(heme) and a hydrogen atom, and obtained a value of 87.5 kcal mol^−1^. Based on the difference in energy between BDE2_O‐H_ and BDE2_water_, the aliphatic hydrogen‐atom abstraction should be followed by an exothermic second hydrogen‐atom abstraction from the phenol group by −8.4 kcal mol^−1^, whereas initial phenol activation should be followed by an endothermic aliphatic hydrogen‐atom abstraction with an energy of 8.3 kcal mol^−1^ (difference in energy between BDE2_C−H_ and BDE2_water_). As a matter of fact, the reaction energy from ^4^ **IM1**_HA,B_ to ^4^ **IM2**_HA_ is highly exothermic (by 18.1 kcal mol^−1^), in line with the difference in energy of the BDE values. Moreover, it explains why the second barrier has a negligible hydrogen‐atom abstraction barrier. The energy difference between ^2^ **IM1**_HP_ and ^2^ **IM2**_HA_ is −11.0 kcal mol^−1^, which is somewhat lower than the energy predicted based on BDE values and shows that the product is highly stabilized through local hydrogen bonds.

The biradical system was calculated in the triplet and open shell singlet spin states and the energy to close the ring to form acetal products gave a BDE_rc_ of 47.7 kcal mol^−1^. However, several hydrogen bonds are lost between the bound water molecule and the product complex upon ring‐closure, so that the stabilization energy for the ring‐closure is much lower than this. Therefore, an energy difference of Δ*E*+ZPE=28.5 kcal mol^−1^ is calculated between ^4^ **IM2**_HA,B_ and ^4^ **Pr**_rc,B_, in line with the energy difference required to close the acetal ring and the cost of breaking several short hydrogen bonds.

From the calculations it is clear that when the phenol group of the substrate is accessible by CpdI, a hydrogen‐atom abstraction from the O−H group will take place because its O−H bond is much weaker than aliphatic C−H bonds such as those of the methoxy group. If hemiacetal is the preferred product, however, the substrate should be positioned with the phenol group pointing away from CpdI, while at the same time the methoxy group points to CpdI.

**Figure 6:**
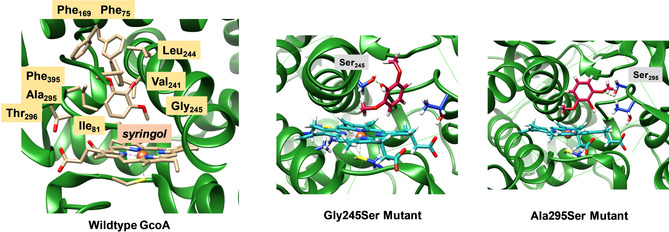
Extract of the substrate‐bound GcoA structure as taken from the 5OMU pdb file and two predicted mutants, namely Gly245Ser and Ala295Ser that position substrate differently.

To gain insight into probable product distributions based on substrate positioning in the enzyme, we analyzed the substrate binding pocket in more detail. Figure 6 displays the active site structure and key residues in the substrate binding pocket of GcoA. The substrate binding pocket is aligned with mostly aromatic and aliphatic amino acid residues including Phe_75_, Ile_81_, Phe_169_, Val_241_, Leu_244_, Ala_295_ and Phe_395_. Therefore, few polar interactions are available to position the substrate in a specific orientation. Probably, substrate positioning in GcoA enzymes is not important as long as the lignin degradation pathways proceed, and the selectivity of the enzyme seems limited.

To make GcoA more substrate‐ and regio‐selective, we decided to create two in silico mutants, whereby an additional hydroxyl group in the substrate binding pocket was included that could position the substrate better and tighter. To this end, we took the 5OMU pdb file, removed the substrate, and created the Gly245Ser and Ala295Ser mutants. Subsequently, using Autodock,^23^ syringol was docked into the substrate binding pocket. The lowest energy syringol bound conformation of the two mutants is shown in Figure 6. As can be seen, the Gly245Ser mutant gives a hydrogen bond between the Ser_245_ and phenolic O−H group of the substrate. This positions the methoxy group close to the heme and the phenol group away from the heme. We predict that the Gly245Ser mutant, therefore, will give predominantly methoxy hydroxylation or *O*‐demethylation products. In contrast, the Ala295Ser mutant has the substrate bound with a hydrogen bond between Ser_295_ and the oxygen atom of one of the methoxy groups. This structure has the other methoxy group and the phenol group both pointing towards the heme and are likely positioned to convert substrate into acetal products.

The GcoA wild‐type structure, however, has a tight and closed substrate binding pocket in which the substrate is locked in by bulky aromatic residues such as those of Phe_75_, Phe_169_ and Phe_395_. Therefore, GcoA should only be able to bind relatively small substrates such as syringol. Actually, experimental studies showed only activity with lignin monomers, which indicates that the substrate binding pocket is closed and only accessible to small substrates. In particular, slow guaiacol and even slower syringol activation by GcoA was observed.5b Furthermore, mutations of Phe_169_ by Ala enabled syringol activation with better turnover numbers, but only *O*‐demethylation products were obtained. Clearly, substrate binding in wild‐type GcoA positions the substrate with the phenol group away from the heme centre and drives the reaction via pathway **A** to give predominantly methoxy hydroxylation followed by deformylation. Based on the structural analysis in this work, it is clear that acetal products from syringol activation in GcoA will require further mutations to position the substrate better and enhance its selectivity. This could also be done by opening the substrate binding pocket so that longer lignin molecules or components can be inserted into the heme active site, which will enable its oxidation.

## Conclusions

A computational study has been presented on lignin activation by the cytochrome P450 isozyme GcoA. We tested several substrate‐binding orientations and spin‐state structures. The work shows that syringol activation should predominantly lead to acetal products through two sequential hydrogen‐atom abstraction steps from the phenol and methoxy groups, followed by radical coupling to close the acetal ring. We then analyzed P450 structures and give suggestions on how to engineer the P450 and give higher contribution of hemiacetal and acetal products. Overall, the work shows that the P450s are efficient oxidants and should be able to activate and degrade lignin molecules easily. The fact that this does not happen regularly in nature reflects the point that the substrate binding pocket is accessible to small substrates only and that it will require some protein engineering to enable it to bind large lignin strands.

## Experimental Section

**Computational methods**: The calculations reported in this work were done using density functional theory methods as implemented in Gaussian 09 software package.^24^ In general, the unrestricted B3LYP hybrid density functional method^25^ was employed in combination with a basis set containing an LANL2DZ+ECP on iron and 6‐31G* on the rest of the atoms (basis set BS1).^26^ Full geometry optimization and frequencies were run for all structures at UB3LYP/BS1 in the gas‐phase. Subsequent single‐point calculations with the conductor‐like polarizable continuum model (CPCM) were performed with a dielectric constant mimicking ethylphenylether,^27^ and a triple‐*ζ* quality basis set (basis set BS2): LACV3P+with ECP on iron and 6‐311+G* on the rest of the atoms. In previous work we extensively tested and benchmarked models and methods for P450 reaction mechanisms and well reproduced experimental structures and rate constants.^28^ These studies showed that the electronic configuration of CpdI and the general reaction mechanisms are little affected by the choice of the density functional method or basis set, and most methods predict close‐lying doublet and quartet spin configurations with similar hydrogen atom abstraction barriers.

## Conflict of interest

The authors declare no conflict of interest.

## Supporting information

As a service to our authors and readers, this journal provides supporting information supplied by the authors. Such materials are peer reviewed and may be re‐organized for online delivery, but are not copy‐edited or typeset. Technical support issues arising from supporting information (other than missing files) should be addressed to the authors.

Supplementary
