# Supplementary material for: Lignin Biodegradation by a Cytochrome P450 Enzyme: A Computational Study into Syringol Activation by GcoA
Source: Chemistry. 2020 Sep 16;26(57):13093–102. doi: 10.1002/chem.202002203 (PMC7590115; doi:10.1002/chem.202002203)
Supplement: Supplementary file 1 — Supplementary [file CHEM-26-13093-s001.pdf]

Chemistry–A European Journal

Supporting Information

Lignin Biodegradation by a Cytochrome P450 Enzyme: A Computational Study into Syringol Activation by GcoA

Hafiz Saqib Ali,[a, b] Richard H. Henchman,[a, b] and Sam P. de Visser*[a, c]

Methods:

Model set-up:

Our model set-up follows previously reported methods and will be briefly summarized here.[1] We started our work from the 5OMU protein databank (pdb) file,[2] which is a P450 monomer structure with of GcoA with syringol bound. The model was based on the active site of this pdb structure with residues selected as highlighted in Scheme S1 below. We took the heme and kept all side chains except the propionate groups, which were replaced by methyl. The axial cysteinate (Cys_{356}) was included as methylmercaptate and iron(III)-heme replaced by iron(IV)-oxo heme. The substrate binding pocket was included through the residues Ile_{81} (as butane), Phe_{169} (as ethylbenzene) and Phe_{395}. In addition, two elaborate protein chains were included in the model, namely the chain Val_{241}-Tyr_{242}-Leu_{243}-Leu_{244}-Gly_{245}-Ala_{246}-Met_{247}-Gln_{248}-Glu_{249} and Ile_{292}-Trp_{293}-Asn_{294}-Ala_{295}-Thr_{296}. The amino acid side chains pointing away from the substrate binding pocket were replaced by Gly, i.e. Tyr_{242}, Leu_{243}, Met_{247}, Trp_{293} and Asn_{294}. The complete model (model **A**) had a total of 302 atoms and was calculated in the doublet and quartet spin states. In addition, an alternative substrate-bound orientation was considered (model **B**), where the substrate was manually rotated to form interactions with the heme through the phenol and one of the methoxy groups, see Scheme S2.

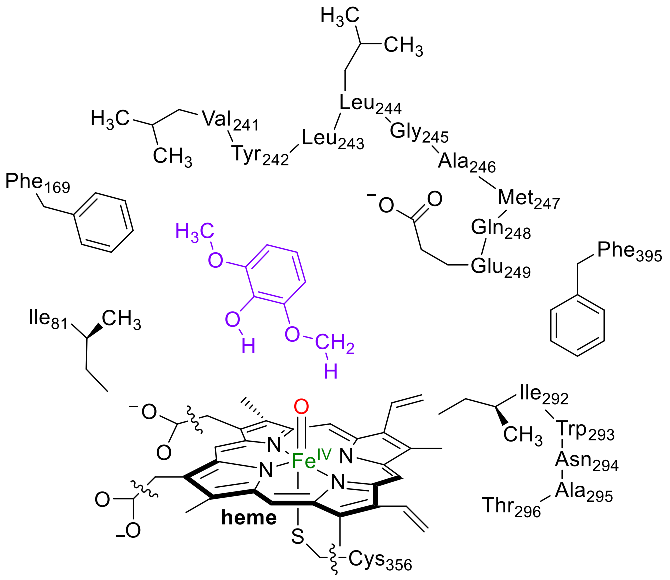

Scheme S1: DFT cluster model investigated in this work.

Computational methods:

Calculations were done using density functional theory methods as implemented in Gaussian-09.[3] In previous work we extensively tested and benchmarked models and methods for P450 reaction mechanisms and reproduced experimental structures and rate constants well.[4] All calculations used the unrestricted B3LYP hybrid density functional method,[5] in combination with a basis set containing an LANL2DZ + ECP on iron and 6-31G* on the rest of the atoms (basis set BS1).[6] Full geometry optimization and frequencies were run for all structures at UB3LYP/BS1 in the gas-phase. Subsequent single point calculations with the polarized continuum model (CPCM) were performed with a dielectric constant mimicking chlorobenzene,[7] and a triple-ζ quality basis set (Basis set BS2): LACV3P+ + ECP on iron and 6-311+G* on the rest of the atoms.

References:

1. a) M. G. Quesne, T. Borowski, S. P. de Visser, *Chem. Eur. J.* **2016**, *22*, 2562; b) A. S. Faponle, M. G. Quesne, S. P. de Visser, *Chem. Eur. J.* **2016**, *22*, 5478.
2. S. J. B. Mallinson, M. M. Machovina, R. L. Silveira, M. Garcia-Borràs, N. Gallup, C. W. Johnson, M. D. Allen, M. S. Skaf, M. F. Crowley, E. L. Neidle, K. N. Houk, G. T. Beckham, J. L. DuBois, J. E. McGeehan, *Nature Commun*. **2018**, *9*, 2487.
3. Gaussian-09, Revision D.01, M. J. Frisch, G. W. Trucks, H. B. Schlegel, G. E. Scuseria, M. A. Robb, J. R. Cheeseman, G. Scalmani, V. Barone, B. Mennucci, G. A. Petersson, H. Nakatsuji, M. Caricato, X. Li, H. P. Hratchian, A. F. Izmaylov, J. Bloino, G. Zheng, J. L. Sonnenberg, M. Hada, M. Ehara, K. Toyota, R. Fukuda, J. Hasegawa, M. Ishida, T. Nakajima, Y. Honda, O. Kitao, H. Nakai, T. Vreven, J. A. Montgomery, Jr., J. E. Peralta, F. Ogliaro, M. Bearpark, J. J. Heyd, E. Brothers, K. N. Kudin, V. N. Staroverov, T. Keith, R. Kobayashi, J. Normand, K. Raghavachari, A. Rendell, J. C. Burant, S. S. Iyengar, J. Tomasi, M. Cossi, N. Rega, J. M. Millam, M. Klene, J. E. Knox, J. B. Cross, V. Bakken, C. Adamo, J. Jaramillo, R. Gomperts, R. E. Stratmann, O. Yazyev, A. J. Austin, R. Cammi, C. Pomelli, J. W. Ochterski, R. L. Martin, K. Morokuma, V. G. Zakrzewski, G. A. Voth, P. Salvador, J. J. Dannenberg, S. Dapprich, A. D. Daniels, O. Farkas, J. B. Foresman, J. V. Ortiz, J. Cioslowski and D. J. Fox, Gaussian, Inc., Wallingford CT, 2010.
4. a) S. Kumar, A. S. Faponle, P. Barman, A. K. Vardhaman, C. V. Sastri, D. Kumar, S. P. de Visser, *J. Am. Chem. Soc*. **2014**, *136*, 17102; b) F. G. Cantú Reinhard, A. S. Faponle, S. P. de Visser, *J. Phys. Chem. A* **2016**, *120*, 9805.
5. a) A. D. Becke, *J. Chem. Phys*. **1993**, *98*, 5648; b) C. Lee, W. Yang, R. G. Parr, *Phys. Rev. B* **1988**, *37*, 785.
6. a) P. J. Hay, W. R. Wadt, *J. Chem. Phys*. **1985**, *82*, 270; b) W. J. Hehre, R. Ditchfield, J. A. Pople, *J. Chem. Phys*. **1972**, *56*, 2257.
7. J. Tomasi, B. Mennucci, R. Cammi, *Chem. Rev*. **2005**, *105*, 2999.

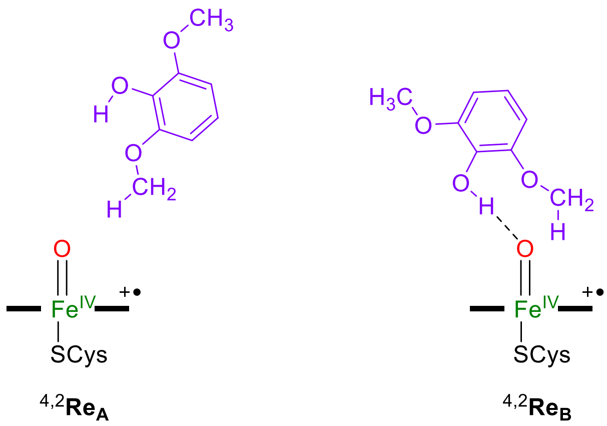

Scheme S2: Substrate binding positions A and B.

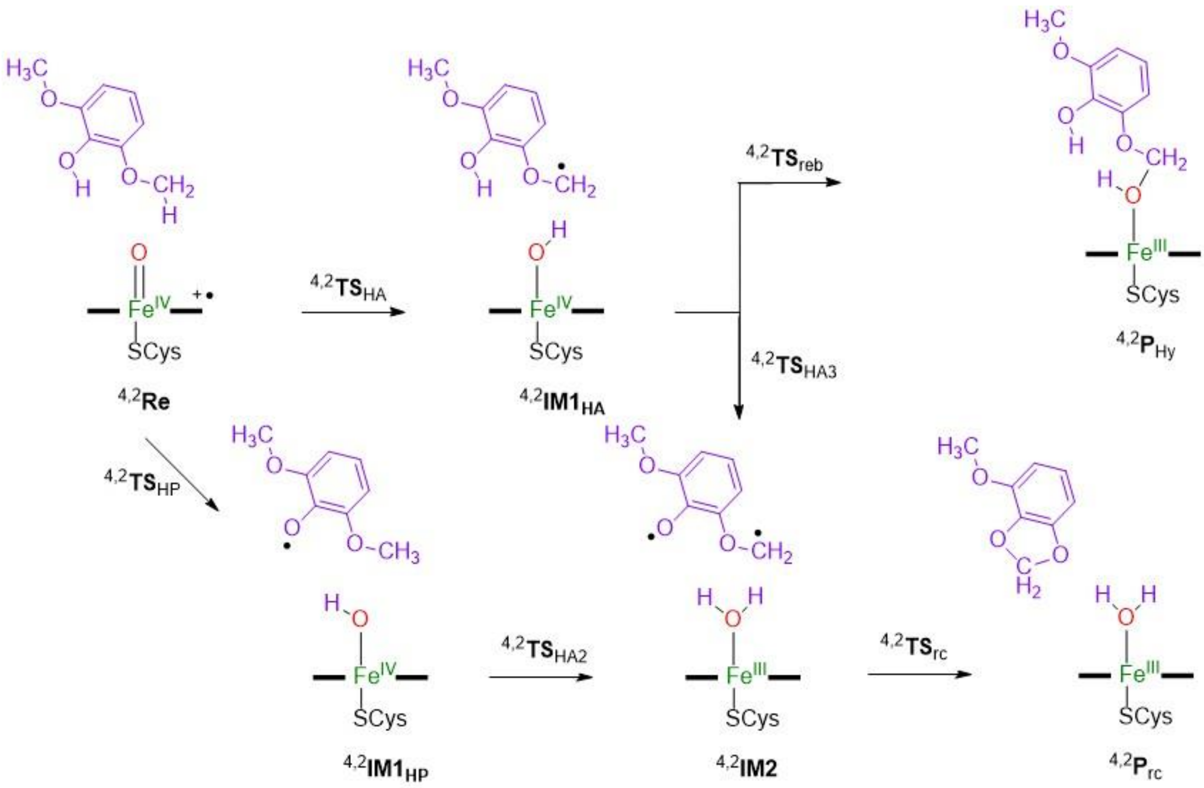

Scheme S3: Reaction mechanism and definition of structures.

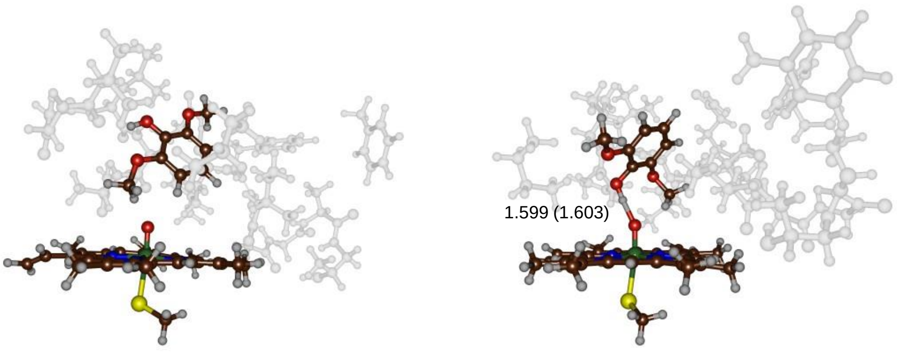

$^{4}Re_{A}$ ($^{2}Re_{A}$)

FeO: 1.649 (1.647)
FeS: 2.644 (2.645)

$^{4}Re_{B}$ ($^{2}Re_{B}$)

FeO: 1.662 (1.663)
FeS: 2.581 (2.584)

Figure S1. UB3LYP/BS1 optimized geometries of the reactant complexes in substrate binding positions A and B. Bond lengths are in angstroms.

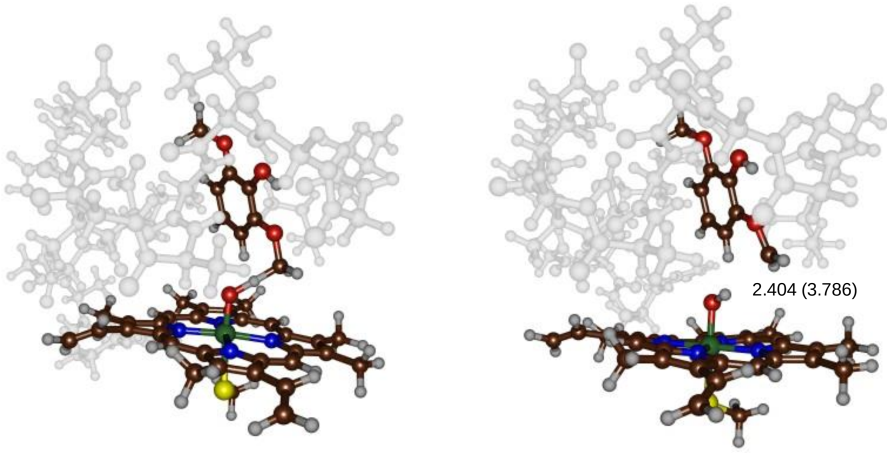

$^{4}TS_{HA,A}$ ($^{2}TS_{HA,A}$)

FeO: 1.735 (1.786)
FeS: 2.492 (2.543)

i1221 (i864) cm^{-1}

CH: 1.286 (1.448)
OH: 1.193 (1.117)
∠CHO: 178° (174°)
∠FeOC: 126° (119°)

$^{4}IM1_{A}$ ($^{2}IM1_{A}$)

FeO: 1.822 (1.836)
FeS: 2.350 (2.488)

Figure S2. UB3LYP/BS1 optimized geometries of $^{4,2}TS_{HA,A}$ and $^{4,2}IM1_{A}$ complexes with substrate in binding position A. Bond lengths are in angstroms, angles in degrees and the imaginary frequencies in cm^{-1}.

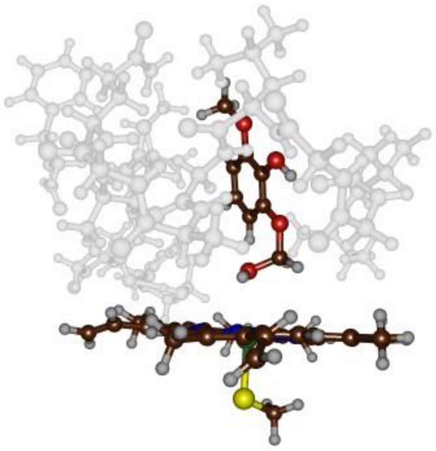

$^4P_{hy}$ ($^2P_{hy}$)

FeO: 2.852 (2.193)
FeS: 2.467 (2.283)

Figure S3. UB3LYP/BS1 optimized geometries of the product complexes ($^{4,2}P_{hy}$) in substrate binding positions A. Bond lengths are in angstroms.

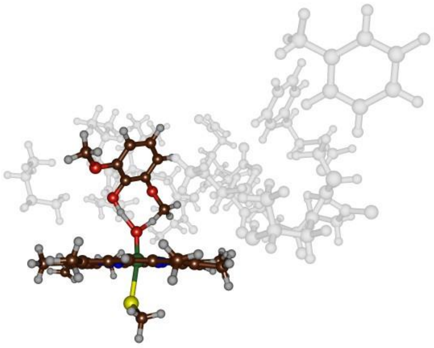

$^4TS_{HA,B}$ ($^2TS_{HA,B}$)

FeO: 1.737 (1.811)
FeS: 2.555 (2.482)

i897 (i1536) cm^{-1}

CH: 1.210 (1.324)
OH: 1.292 (1.203)
∠CHO: 169° (164°)
∠FeOC: 127° (124°)

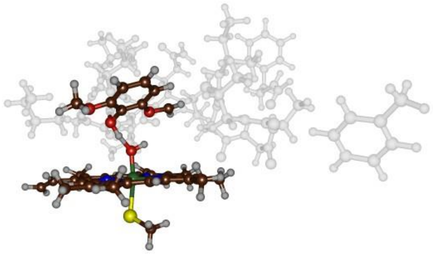

$^4IM1_B$

FeO: 1.865
FeS: 2.449

Figure S4. UB3LYP/BS1 optimized geometries of $^{4,2}TS_{HA,B}$ and $^4IM1_B$ complexes with substrate in binding position B. Bond lengths are in angstroms, angles in degrees and the imaginary frequencies in cm^{-1}.

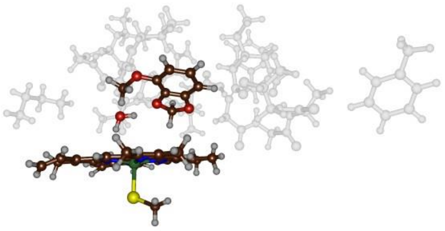

$^{4}P_{rc}$ ($^{2}P_{rc}$)

FeO: 3.565 (3.491)
FeS: 2.454 (2.294)

Figure S5. UB3LYP/BS1 optimized geometries of the product complexes ($^{4,2}P_{rc}$) in substrate binding positions B. Bond lengths are in angstroms.

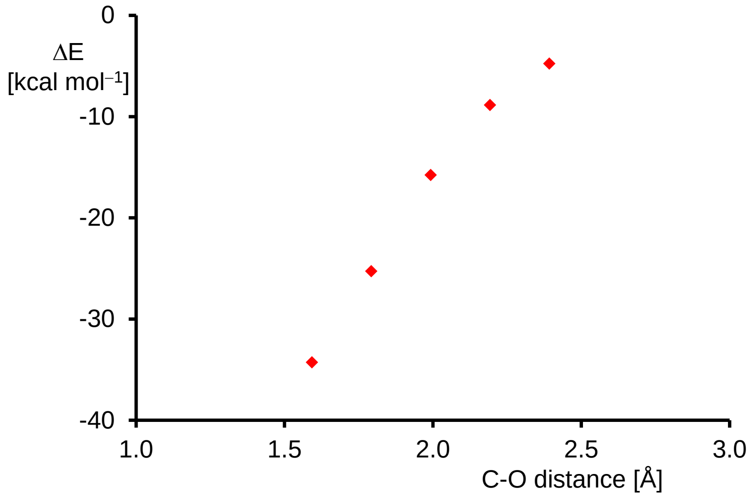

Figure S6. UB3LYP/BS1 calculated geometry scan for ring-closure from ^{4}IM2$_{B}$ as calculated in Gaussian. As follows, the ring-closure is barrierless and collapses to acetal products with large driving force.

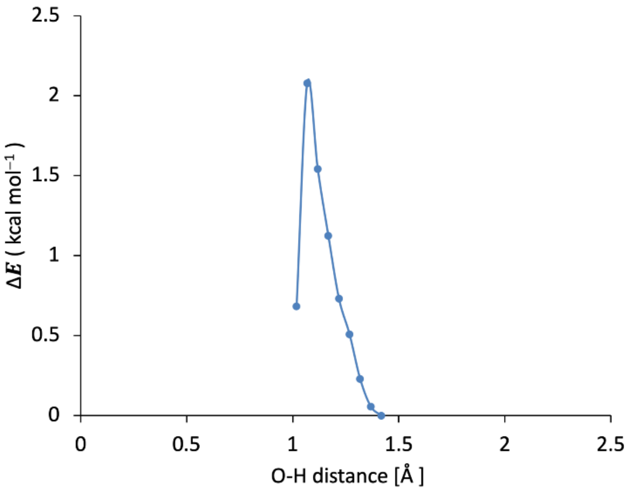

Figure S7. UB3LYP/BS1 calculated constraint geometry scan for hydrogen-atom abstraction from ^{4}IM1$_{HP}$ as calculated in Gaussian. An estimate for the barrier height from the scan is 2.1 kcal mol^{-1} above the energy of ^{4}IM1$_{HP,B}$.

Table S1: Absolute (free) energies (in au) of optimized geometries of the GcoA model with substrate in binding position A for the reaction of CpdI with syringol as calculated at the UB3LYP level of theory in the quartet spin state.

System	BS1			BS2 + Solv		
	E	E+ZPE	G	E	E+ZPE	G
^{4}**Re$_{A}$**	-7255.578397	-7253.012720	-7253.254548	-7259.274122	-7256.708445	-7256.950273
^{4}**TS$_{HA,A}$**	-7255.553260	-7252.993718	-7253.231125	-7259.243211	-7256.683669	-7256.921076
^{4}**IM1$_{A}$**	-7255.574127	-7253.011282	-7253.252258	-7259.277707	-7256.714862	-7256.955838
^{4}**P$_{hy,A}$**	-7255.674710	-7253.106792	-7253.355713	-7259.379000	-7256.811082	-7257.060003

Table S2: Absolute (free) energies (in au) of optimized geometries of the GcoA model with substrate in binding position A for the reaction of CpdI with syringol as calculated at the UB3LYP level of theory in the doublet spin state.

System	BS1			BS2 + Solv		
	E	E+ZPE	G	E	E+ZPE	G
^{2}**Re$_{A}$**	-7255.563193	-7252.998440	-7253.250637	-7259.275915	-7256.711162	-7256.963359
^{2}**TS$_{HA,A}$**	-7255.544692	-7252.985319	-7253.227948	-7259.245344	-7256.685971	-7256.928600
^{2}**IM1$_{A}$**	-7255.590447	-7253.027783	-7253.271366	-7259.276776	-7256.714112	-7256.957695
^{2}**P$_{hy,A}$**	-7255.680062	-7253.110657	-7253.348281	-7259.379045	-7256.809640	-7257.047263

Table S3: Relative (free) energies (in kcal mol^{-1}) of optimized geometries of the GcoA model with substrate in binding position A for the reaction of CpdI with syringol as calculated at the UB3LYP level of theory in the quartet spin state.

System	BS1			BS2 + Solv		
	ΔE	ΔE+ZPE	ΔG	ΔE	ΔE+ZPE	ΔG
^{4}**Re$_{A}$**	0.00	0.00	0.00	0.00	0.00	0.00
^{4}**TS$_{HA,A}$**	15.77	11.92	14.70	19.40	15.55	18.32
^{4}**IM1$_{A}$**	2.68	0.90	1.44	-2.25	-4.03	-3.49
^{4}**P$_{hy,A}$**	-60.44	-59.03	-63.48	-65.81	-64.41	-68.86

Table S4: Relative (free) energies (in kcal mol^{-1}) of optimized geometries of the GcoA model with substrate in binding position A for the reaction of CpdI with syringol as calculated at the UB3LYP level of theory in the doublet spin state.

System	BS1			BS2 + Solv		
	ΔE	ΔE+ZPE	ΔG	ΔE	ΔE+ZPE	ΔG
^{2}**Re$_{A}$**	9.54	8.96	2.45	-1.13	-1.71	-8.21
^{2}**TS$_{HA,A}$**	21.15	17.19	16.69	18.06	14.10	13.60
^{2}**IM1$_{A}$**	-7.56	-9.45	-10.55	-1.67	-3.56	-4.66
^{2}**P$_{hy,A}$**	-63.80	-61.46	-58.82	-65.84	-63.50	-60.86

Table S5: Absolute (free) energies (in au) of optimized geometries of the GcoA model with substrate in binding position B for the reaction of CpdI with syringol as calculated at the UB3LYP level of theory in the quartet spin state.

System	BS1			BS2 + Solv		
	E	E+ZPE	G	E	E+ZPE	G
^{4}Re$_{B}$	-7255.593352	-7253.028498	-7253.269319	-7259.284697	-7256.719843	-7256.960664
^{4}TS$_{HA,B}$	-7255.564815	-7253.005395	-7253.249018	-7259.252571	-7256.693151	-7256.936774
^{4}TS$_{HP,B}$	-7255.587777	-7253.027429	-7253.268037	-7259.278818	-7256.718470	-7256.959078
^{4}IM1$_{HA,B}$	-7255.625270	-7253.061131	-7253.299135	-7259.293135	-7256.728996	-7256.967000
^{4}IM1$_{HP,B}$	-7255.625274	-7253.060555	-7253.298238	-7259.309968	-7256.745249	-7256.982932
^{4}IM2$_{B}$	-7255.632056	-7253.066895	-7253.312748	-7259.322990	-7256.757829	-7257.003682
^{4}P$_{rc,B}$	-7255.682869	-7253.115059	-7253.360926	-7259.375333	-7256.807523	-7257.053390

Table S6: Absolute (free) energies (in au) of optimized geometries of the GcoA model with substrate in binding position B for the reaction of CpdI with syringol as calculated at the UB3LYP level of theory in the doublet spin state.

System	BS1			BS2 + Solv		
	E	E+ZPE	G	E	E+ZPE	G
^{2}Re$_{B}$	-7255.594910	-7253.030065	-7253.276323	-7259.285803	-7256.720958	-7256.967216
^{2}TS$_{HA,B}$	-7255.567349	-7253.010301	-7253.250925	-7259.256400	-7256.699352	-7256.939976
^{2}TS$_{HP,B}$	-7255.588393	-7253.028341	-7253.268406	-7259.279534	-7256.719482	-7256.959547
^{2}IM2$_{B}$	-7255.643661	-7253.076743	-7253.318535	-7259.328994	-7256.762076	-7257.003868
^{2}IM1$_{HP,B}$	-7255.635597	-7253.070448	-7253.313513	-7259.309753	-7256.744604	-7256.987669
^{2}P$_{rc,B}$	-7255.681542	-7253.113209	-7253.358315	-7259.370754	-7256.802421	-7257.047526

Table S7: Relative (free) energies (in kcal mol^{-1}) of optimized geometries of the GcoA model with substrate in binding position B for the reaction of CpdI with syringol as calculated at the UB3LYP level of theory in the quartet spin state.

System	BS1			BS2 + Solv		
	ΔE	ΔE+ZPE	ΔG	ΔE	ΔE+ZPE	ΔG
^{4}Re$_{B}$	0.98	0.98	4.40	0.69	0.70	4.11
^{4}TS$_{HA,B}$	17.91	14.50	12.74	20.16	16.75	14.99
^{4}TS$_{HP,B}$	3.50	0.67	0.80	3.69	0.86	0.99
^{4}IM1$_{HA,B}$	-19.05	-19.49	-14.31	-4.60	-5.04	0.14
^{4}IM1$_{HP,B}$	-20.03	-20.12	-18.15	-15.86	-15.94	-13.97
^{4}IM2$_{B}$	-23.31	-23.11	-22.86	-23.34	-23.14	-22.88
^{4}P$_{rc,B}$	-60.44	-59.03	-63.48	-65.81	-64.41	-68.86

Table S8: Relative (free) energies of optimized geometries of the GcoA model with substrate in binding position B for the reaction of CpdI with syringol as calculated at the UB3LYP level of theory in the doublet spin state.

System	BS1			BS2 + Solv		
	ΔE	ΔE+ZPE	ΔE+G	ΔE	ΔE+ZPE	ΔE+G
^{2}Re$_{B}$	0.00	0.00	0.00	0.00	0.00	0.00
^{2}TS$_{HA,B}$	18.54	13.04	20.52	17.95	12.45	19.93
^{2}TS$_{HP,B}$	4.09	1.08	4.97	3.93	0.93	4.81
^{2}IM2$_{B}$	-30.59	-29.29	-26.49	-27.10	-25.80	-23.00
^{2}IM1$_{HP,B}$	-25.53	-25.34	-23.34	-15.03	-14.84	-12.83
^{2}P$_{rc,B}$	-54.36	-52.17	-51.45	-53.31	-51.12	-50.40

Table S9: Group spin densities of optimized geometries of the GcoA model with substrate in binding position A for the reaction of CpdI with syringol as calculated at the UB3LYP level of theory in the quartet spin state.

System		Fe	O	Porphyrin	Substrate	Amino Acids	Total
$^{4}Re_{A}$	Gas	1.1285	0.8872	0.3007	0.0039	0.6797	3.00
	Solvent	1.2094	0.8523	0.2659	-0.0022	0.6747	3.00
$^{4}TS_{HA,A}$	Gas	1.0885	0.7173	0.2625	0.3481	0.5836	3.00
	Solvent	1.1982	0.6856	0.1829	0.3711	0.5622	3.00
$^{4}IM1_{A}$	Gas	1.0477	0.4262	0.1930	0.7391	0.5940	3.00
	Solvent	2.1810	0.2233	-0.3469	0.9273	0.0153	3.00
$^{4}P_{hy,A}$	Gas	2.3699	-0.0310	-0.0008	0.0653	0.5966	3.00
	Solvent	2.9486	-0.0520	-0.2684	0.0034	0.3684	3.00

Table S10: Group spin densities of optimized geometries of the GcoA model with substrate in binding position A for the reaction of CpdI with syringol as calculated at the UB3LYP level of theory in the doublet spin state.

System		Fe	O	Porphyrin	Substrate	Amino Acids	Total
$^{2}Re_{A}$	Gas	1.2271	0.8676	-0.3818	-0.0005	-0.7124	1.00
	Solvent	1.6196	0.8001	-0.6813	-0.0028	-0.7356	1.00
$^{2}TS_{HA,A}$	Gas	2.1321	0.1078	-0.1904	-0.6233	-0.4262	1.00
	Solvent	2.3529	0.0146	-0.4305	-0.5896	-0.3474	1.00
$^{2}IM1_{A}$	Gas	1.0350	0.0741	-0.4980	0.9692	-0.5802	1.00
	Solvent	1.2893	0.0596	-0.7481	0.9184	-0.5192	1.00
$^{2}P_{hy,A}$	Gas	1.5013	0.0024	-0.1749	0.0004	-0.3292	1.00
	Solvent	1.2460	-0.0050	-0.2334	0.0018	-0.0094	1.00

Table S11: Group charges of optimized geometries of the GcoA model with substrate in binding position A for the reaction of CpdI with syringol as calculated at the UB3LYP level of theory in the quartet spin state.

System		Fe	O	Porphyrin	Substrate	Amino Acids	Total
$^{4}Re_{A}$	Gas	0.5200	-0.3516	-0.5701	0.0219	-0.6201	-1.00
	Solvent	-4.1291	0.4298	3.0122	-0.0634	-0.2494	-1.00
$^{4}TS_{HA,A}$	Gas	0.4798	-0.4965	-0.6033	0.1698	-0.5498	-1.00
	Solvent	-4.1630	0.3290	3.0256	-0.0015	-0.1900	-1.00
$^{4}IM1_{A}$	Gas	0.4680	-0.5806	-0.6634	0.3236	-0.5476	-1.00
	Solvent	-4.6799	0.2955	3.2597	0.2049	-0.0802	-1.00
$^{4}P_{hy,A}$	Gas	0.3956	-0.3087	-0.7686	0.4195	-0.7379	-1.00
	Solvent	-1.6188	0.3660	1.6624	-0.4837	-0.9259	-1.00

Table S12: Group charges of optimized geometries of the GcoA model with substrate in binding position A for the reaction of CpdI with syringol as calculated at the UB3LYP level of theory in the doublet spin state.

System		Fe	O	Porphyrin	Substrate	Amino Acids	Total
$^{2}Re_{A}$	Gas	0.5093	-0.3562	-0.5509	0.0542	-0.6564	-1.00
	Solvent	-3.6824	0.3109	2.7434	-0.0445	-0.3273	-1.00
$^{2}TS_{HA,A}$	Gas	0.5139	-0.5835	-0.6446	0.3310	-0.6168	-1.00
	Solvent	-4.6877	0.3381	3.3200	0.1355	-0.1059	-1.00
$^{2}IM1_{A}$	Gas	0.4515	-0.6395	-0.6581	0.3605	-0.5144	-1.00
	Solvent	-3.4456	0.1562	2.4707	0.2230	-0.4044	-1.00
$^{2}P_{hy,A}$	Gas	0.4183	-0.5591	-0.8557	0.6691	-0.6726	-1.00
	Solvent	-2.6748	0.3752	2.1005	-0.4476	-0.3533	-1.00

Table S13: Group spin densities of optimized geometries of the GcoA model with substrate in binding position B for the reaction of CpdI with syringol as calculated at the UB3LYP level of theory in the quartet spin state.

System		Fe	O	Porphyrin	Substrate	Amino Acids	Total
^4Re_B	Gas	1.2600	0.7676	0.3422	0.0000	0.6302	3.00
	Solvent	1.3457	0.7787	0.2620	-0.0278	0.6414	3.00
$^4TS_{HA,B}$	Gas	1.1039	0.6525	0.3532	0.2677	0.6227	3.00
	Solvent	1.3457	0.7787	0.2620	-0.0278	0.6414	3.00
$^4TS_{HP,B}$	Gas	1.2600	0.7676	0.3422	0.0000	0.6302	3.00
$^4IM1_{HA,B}$	Gas	2.5060	0.0477	-0.0634	0.0070	0.5028	3.00
	Solvent	3.0415	-0.0174	-0.3682	0.0110	0.3330	3.00
$^4IM1_{HP,B}$	Gas	0.9293	0.1355	0.3525	0.9858	0.5970	3.00
	Solvent	0.9330	0.1186	0.3714	0.9772	0.5998	3.00
$^4IM2_B$	Gas	2.5060	0.0477	-0.0634	0.0070	0.5028	3.00
	Solvent	3.0415	-0.0174	-0.3682	0.0110	0.3330	3.00
$^4P_{rc,B}$	Gas	2.4405	0.0007	0.0453	-0.0040	0.5175	3.00
	Solvent	2.4739	0.0064	0.0966	-0.0017	0.4249	3.00

Table S14: Group spin densities of optimized geometries of the GcoA model with substrate in binding position B for the reaction of CpdI with syringol as calculated at the UB3LYP level of theory in the doublet spin state.

System		Fe	O	Porphyrin	Substrate	Amino Acids	Total
^2Re_B	Gas	1.3897	0.7430	-0.4488	-0.0018	-0.6821	1.00
	Solvent	1.6637	0.7375	-0.6937	-0.0273	-0.6801	1.00
$^2TS_{HA,B}$	Gas	1.2235	0.6195	-0.4232	0.2521	-0.6718	1.00
	Solvent	1.3286	0.4912	-0.6516	0.4660	-0.6343	1.00
$^2TS_{HP,B}$	Gas	1.6135	0.5728	-0.5862	-0.0197	-0.5804	1.00
	Solvent	1.9320	0.5623	-0.8914	-0.0395	-0.5634	1.00
$^2IM1_{HA,B}$	Gas	1.0898	0.0376	-0.4910	0.9549	-0.5913	1.00
	Solvent	1.2294	-0.0009	-0.2370	-0.0009	0.0094	1.00
$^2IM1_{HP,B}$	Gas	0.9374	0.1503	0.3250	-1.0006	0.5880	1.00
	Solvent	0.9309	0.1187	0.3723	-0.9971	0.5751	1.00
$^2P_{rc,B}$	Gas	1.5651	0.0016	-0.2060	-0.0013	-0.3594	1.00
	Solvent	1.3163	0.0060	-0.2225	0.0013	-0.1011	1.00

Table S15: Group charges of optimized geometries of the GcoA model with substrate in binding position A for the reaction of CpdI with syringol as calculated at the UB3LYP level of theory in the quartet spin state.

System		Fe	O	Porphyrin	Substrate	Amino Acids	Total
^4Re_B	Gas	0.5649	-0.4987	-0.5343	0.0490	-0.5809	-1.00
	Solvent	-4.5483	0.6225	3.3443	-0.4160	-0.0026	-1.00
$^4TS_{HA,B}$	Gas	0.5443	-0.5898	-0.5539	0.1804	-0.5811	-1.00
	Solvent	-4.5483	0.6225	3.3443	-0.4160	-0.0026	-1.00
$^4TS_{HP,B}$	Gas	0.5717	-0.5932	-0.4004	-0.0094	-0.5687	-1.00
$^4IM1_{HA,B}$	Gas	0.4964	-0.7592	-0.9086	0.8766	-0.7052	-1.00
	Solvent	-2.9980	0.0834	2.4247	0.2829	-0.7930	-1.00
$^4IM1_{HP,B}$	Gas	0.4531	-0.6281	-0.6400	0.3897	-0.5747	-1.00
	Solvent	-1.9818	0.0280	1.3370	0.4000	-0.7832	-1.00
$^4IM2_B$	Gas	0.4964	-0.7592	-0.9086	0.8766	-0.7052	-1.00
	Solvent	-2.9980	0.0834	2.4247	0.2829	-0.7930	-1.00
$^4P_{rc,B}$	Gas	0.4651	-0.7943	-0.7741	0.9055	-0.8021	-1.00
	Solvent	-2.1762	-0.5324	1.2868	0.8862	-0.4643	-1.00

Table S16: Group charges of optimized geometries of the GcoA model with substrate in binding position B for the reaction of CpdI with syringol as calculated at the UB3LYP level of theory in the doublet spin state.

System		Fe	O	Porphyrin	Substrate	Amino Acids	Total
**^{2}Re$_B$**	**Gas**	0.5559	-0.5000	-0.5126	0.0551	-0.5984	-1.00
	**Solvent**	-4.5077	0.6561	3.2920	-0.5629	0.1225	-1.00
**^{2}TS$_{HA,B}$**	**Gas**	0.5407	-0.5912	-0.5524	0.1814	-0.5785	-1.00
	**Solvent**	-4.0860	0.2391	2.8817	-0.1452	0.1103	-1.00
**^{2}TS$_{HP,B}$**	**Gas**	0.5758	-0.5996	-0.3932	-0.0120	-0.5710	-1.00
	**Solvent**	-5.1816	0.4993	3.8571	-0.0392	-0.1354	-1.00
**^{2}IM2$_{HA,B}$**	**Gas**	0.4800	-0.7484	-0.5868	0.3684	-0.5131	-1.00
	**Solvent**	-3.4602	0.2381	2.1731	0.2510	-0.2020	-1.00
**^{2}IM1$_{HP,B}$**	**Gas**	0.4519	-0.6263	-0.6034	0.3950	-0.6172	-1.00
	**Solvent**	-1.9846	0.0297	1.3355	0.3946	-0.7751	-1.00
**^{2}P$_{rc,B}$**	**Gas**	0.3849	-0.7910	-0.7838	0.8942	-0.7042	-1.00
	**Solvent**	-2.4622	-0.5142	1.3103	0.8988	-0.2326	-1.00

Cartesian coordinates:

^{4}Re$_{A}$

C	37.58579700	80.84216300	35.84837800
C	38.14008600	79.61379200	35.09530100
C	37.90667200	80.85199300	37.35264200
C	39.67534400	79.57457000	35.00176900
H	37.98418200	81.75595300	35.38172700
H	37.71905300	79.60356100	34.07911800
H	37.77739700	78.69869500	35.58757100
H	37.47864300	81.73610700	37.84182800
H	37.49145500	79.96318700	37.84629900
H	38.98540500	80.85674300	37.54130200
H	40.01160100	78.70251200	34.42775500
H	40.14747300	79.51839900	35.98821800
H	40.05704000	80.47188600	34.49845500
H	52.74049800	79.20139000	29.07269500
C	51.66919800	79.05809800	29.25935500
C	50.92546200	80.37741400	29.26629200
C	51.40259700	81.46783800	30.01728100
C	49.73447900	80.53969600	28.53994700
C	50.71028400	82.68386100	30.05361000
C	49.03733200	81.75436200	28.56616900
C	49.52195300	82.82615200	29.32375600
H	51.57568800	78.54085000	30.22353300
H	51.28060300	78.38523200	28.48744200
H	52.32756300	81.36147600	30.57882200
H	49.35053100	79.70911400	27.95322400
H	51.08338100	83.50980900	30.65329300
H	48.11749700	81.85934500	27.99795700
H	48.98066600	83.76650600	29.35059800
C	38.43768800	74.59882400	40.68166100
C	38.71685200	75.25363400	42.01718600
O	39.77593300	75.89478100	42.26605300
C	37.30050900	75.28004200	39.86291800
C	37.69388900	76.70671400	39.43691300
C	36.95069000	74.41133500	38.64005000
H	39.36774300	74.62897400	40.10618100
H	36.40411900	75.34023500	40.50273900
H	36.62505700	73.40610700	38.93519300
H	37.82188800	74.30622000	37.98122900
H	36.14548800	74.87216900	38.05579300
H	37.98329700	77.33723300	40.28407600
H	38.55110300	76.68030800	38.75331700
H	36.86083400	77.19292600	38.91621300
N	37.77066700	75.11999300	42.99759500
C	37.87902000	75.87277700	44.25204000
C	39.05442500	75.47730900	45.15159600
O	39.46323600	76.27610400	46.02024600
H	36.90099900	74.65263900	42.78076600
H	38.00635000	76.93953800	44.04674000
N	39.57379500	74.23017300	44.96128700
C	40.72534000	73.73476100	45.70616600
C	41.96829700	73.44108600	44.85909000
O	42.79307900	72.59557700	45.26894900
H	39.16835900	73.64478100	44.24542000
H	40.97310600	74.49775100	46.45147300
N	42.11667400	74.15638100	43.70284200
C	43.29155800	73.95103300	42.82494000
C	44.09012100	75.26426400	42.77579300
O	45.08577000	75.42696700	43.54215900
C	42.87644600	73.34781900	41.46211500
C	44.01317800	72.63098900	40.68927600
C	43.42411800	71.91413300	39.45744300
C	45.15645300	73.57396700	40.26876200
H	41.39026000	74.79965200	43.40299900
H	43.92712500	73.24216600	43.36096100
H	42.09346700	72.60797600	41.67958800
H	42.41789000	74.10808700	40.81409800
H	44.43076500	71.86385300	41.36100000
H	45.91919700	73.03298500	39.69720400
H	45.66674800	74.01333000	41.13128800
H	44.77525700	74.39450200	39.64928400
H	42.63485200	71.20638200	39.74289400
H	42.99270300	72.64180100	38.75717200
H	44.20307800	71.35756600	38.92398400
N	43.63687300	76.24807100	41.96501900
C	44.35205600	77.52335800	41.83297200
C	44.50521900	78.28048900	43.14493400
O	45.45704100	79.07929200	43.33050200
H	42.88856100	76.08335600	41.28785500
H	45.34347900	77.38415200	41.38910000
H	43.78575300	78.14857700	41.13566600
N	43.56288600	78.05395400	44.09976200
C	43.60485000	78.70764200	45.41029100
C	44.96565800	78.52654500	46.10732100
O	45.38014200	79.41950900	46.89156100
C	42.46576100	78.15914200	46.28682900
H	42.79041500	77.43819900	43.88688600
H	43.49284400	79.78782900	45.28045700
H	42.56787500	77.07737800	46.42870700
H	42.48788900	78.63660300	47.26907400
H	41.48842400	78.35416200	45.83408000
N	45.61377700	77.35036600	45.88509700
C	46.95999600	77.09880100	46.38925200
C	48.08963200	77.07237500	45.34655200
O	49.26792100	77.26711900	45.72778500
H	45.20938500	76.67930200	45.23829700
H	47.20191900	77.89048200	47.09941500
N	47.76343200	76.77000800	44.05793800
C	48.79169300	76.61435000	43.02271500
C	49.29324200	77.94058300	42.43152900
O	50.43558500	77.96741500	41.88544800
C	48.22662200	75.72663900	41.87796100
C	49.30997900	75.21164500	40.90544200
C	48.74302700	74.58227400	39.63741600
O	48.94158900	73.37085700	39.35436900
N	48.03307200	75.41009200	38.81974100
H	46.81176300	76.51217900	43.81501100
H	49.66867900	76.14297300	43.47736600
H	47.70990600	74.87344400	42.33284100
H	47.48567200	76.31239900	41.32179300
H	49.92047700	74.44480500	41.39103600
H	49.97163100	76.04235500	40.63509700
H	47.75209400	76.37892100	39.04272900
H	47.67779300	75.00061000	37.96710400
N	48.45027800	78.99963200	42.41961100
C	48.81940800	80.23260700	41.69504600
C	49.71335700	81.12800200	42.57149300
C	47.58264100	80.97763800	41.15994800
C	46.58170200	80.11303900	40.35979900
C	47.21725100	79.14012900	39.36066400
O	48.00559100	79.61977300	38.46562200
O	46.90144900	77.88716000	39.48167300
H	47.53642400	78.94252800	42.86306700
H	49.40662200	79.90392700	40.83438500
H	47.03892900	81.44345100	41.99211500
H	47.96058500	81.79033500	40.52811800
H	45.96818400	79.54853200	41.05728800
H	45.91198800	80.78650100	39.80585700
C	47.91234100	82.58652200	37.25792800
C	48.27284900	83.71122700	38.20724500
O	47.44794300	84.56850200	38.62889200
C	46.57149600	82.77573600	36.51978200
C	46.22133200	81.47231500	35.76092000
C	46.60432700	84.00207500	35.58561300
C	44.80909700	81.45001200	35.15445500
H	48.72449500	82.47203100	36.52332000
H	45.79682300	82.94678100	37.28201300
H	46.96253500	81.32050300	34.96036900
H	46.34029600	80.62730000	36.45144600
H	46.85304200	84.90718800	36.14808100
H	45.63622100	84.16605200	35.09823000
H	47.35614000	83.86426800	34.79473500
H	44.60235900	80.48482300	34.67546300
H	44.04649500	81.60882400	35.92848000
H	44.67551500	82.22837200	34.39348400
N	49.59000600	83.73543700	38.59395800
C	50.12137100	84.80267300	39.44172900
C	51.21079000	85.59900400	38.72006100

O	51.06442300	86.81590100	38.42556600
H	50.19025900	82.93379600	38.41024600
H	49.29999900	85.47985600	39.67277400
N	52.33451500	84.88468500	38.42068800
C	53.50368400	85.47169500	37.77930400
C	53.72623500	85.06274200	36.32024800
O	54.82487800	85.28125400	35.76153900
H	52.34938600	83.87812200	38.62332800
H	53.38360100	86.56064500	37.80010100
N	52.64045000	84.53213100	35.68402900
C	52.59877800	84.23612000	34.24929000
C	51.85850200	85.34268700	33.45766400
O	51.49614800	85.15649700	32.26610200
C	51.96579600	82.86261000	33.99298000
H	51.82613600	84.30768100	36.24173300
H	53.64026400	84.24274400	33.90567400
H	51.87928300	82.68370300	32.92008500
H	52.56695300	82.06887700	34.44390300
H	50.95624300	82.80758200	34.41803800
N	51.65444200	86.49199500	34.13566000
C	50.93873600	87.66579700	33.66074700
C	50.12979100	88.23738300	34.84720100
O	51.08103400	88.32857600	35.95602200
C	48.90186600	87.39499700	35.19690200
H	51.99026900	86.59260700	35.08540500
H	50.29348800	87.38201100	32.82458800
H	49.81245500	89.25753400	34.58902600
H	50.73784400	87.99726400	36.81579900
H	48.35717100	87.82833900	36.04250600
H	48.21667200	87.34444900	34.34280100
H	49.18826100	86.37181000	35.46029200
H	40.29141700	86.92914500	42.34040600
C	41.17493300	87.22321200	42.91523800
S	41.45498000	85.95590600	44.27618900
H	40.99394000	88.19277800	43.38456900
H	42.03971100	87.26583500	42.25261200
C	52.83355000	81.10175500	38.56745900
O	52.00334600	82.05889400	38.58165000
N	52.54888000	79.79852300	38.65290600
C	51.20904500	79.18721400	38.78877600
C	51.02320900	78.01323600	37.80531200
C	51.00329200	78.42818800	36.34719300
C	49.85385700	79.04505600	35.81549000
C	52.11218700	78.22820300	35.50929900
C	49.82825200	79.45534100	34.47869400
C	52.08664300	78.64185500	34.17060600
C	50.94349500	79.25985700	33.65096100
H	50.45530100	79.95236100	38.60146700
H	51.81128900	77.26453400	37.97513200
H	50.07271700	77.53762100	38.06913800
H	48.99685700	79.19476300	36.46904800
H	52.99834300	77.73442700	35.90252200
H	48.93493900	79.92579300	34.07750100
H	52.95388700	78.47762800	33.53662100
H	50.91786900	79.58904900	32.61599600
H	53.33360600	79.15833400	38.64559300
C	39.89559400	84.65087400	40.60521900
C	44.66617300	84.75021300	41.46410200
C	43.92128500	82.58523700	45.73764800
C	39.12803200	82.94406500	45.08511000
C	41.26608900	84.80256900	40.43923100
C	41.89633300	85.34303300	39.24320000
C	43.24574100	85.40781100	39.49889100
C	43.43611600	84.88390600	40.84683500
C	44.34618900	85.92596100	38.62331700
C	41.15846800	85.75715300	38.00532100
H	40.49485700	86.61362800	38.18987400
C	44.87662000	84.18536100	42.71571400
C	46.18860300	83.98365000	43.30926200
C	45.98121300	83.30075300	44.49386300
C	44.53628300	83.13583900	44.62526900
C	47.48167800	84.42409900	42.69509800
C	46.94497000	82.76464000	45.45310700
C	48.13276400	83.31020600	45.77436800
C	42.55334600	82.52474800	45.95264000

C	41.93936100	82.01586400	47.16561600
C	40.57107300	82.13187300	46.99000100
C	40.36864900	82.69807200	45.65928400
C	42.69381500	81.45095700	48.33087500
C	39.48636200	81.74604300	47.88706300
C	39.51005000	81.75218300	49.23274600
C	38.91738200	83.45856000	43.82075000
C	37.61129800	83.65476200	43.21360400
C	37.82784600	84.12028700	41.93738300
C	39.26813900	84.22227200	41.76417600
C	36.30879600	83.38117300	43.90381600
C	36.82355100	84.47217400	40.88142100
H	36.89897200	85.52651900	40.58332000
N	42.21619500	84.52838300	41.40533500
N	43.88475400	83.68430200	43.54002200
N	41.58415100	82.94727100	45.05386100
N	39.92069000	83.82918100	42.92654000
Fe	41.91227100	83.60008500	43.16622800
H	40.53508200	84.94391600	37.61092700
H	41.85278300	86.04948000	37.21257100
H	46.66363100	81.82528700	45.92932100
H	38.56813500	81.41545900	47.40117800
H	35.80061300	84.30410200	41.23029500
H	36.96299400	83.87093700	39.97264300
H	48.47210300	84.25701200	45.36625100
H	48.79870800	82.81861100	46.47600600
H	40.36527800	82.10343600	49.79967900
H	38.65228600	81.41846500	49.80836800
H	35.45811800	83.57770000	43.24471800
H	36.18513700	84.00916100	44.79594700
H	36.23457900	82.33628300	44.23330000
H	44.00393400	86.05311400	37.59131800
H	44.70280000	86.90683500	38.96862100
H	45.21986200	85.26434300	38.60274700
H	42.06335300	80.75779700	48.89737100
H	43.59104700	80.90951500	48.01058600
H	43.01591800	82.23678200	49.02901100
H	48.30513700	83.79118500	43.03697600
H	47.72798700	85.45963200	42.97054000
H	47.45301700	84.37656200	41.60164900
O	41.37794300	76.55714600	40.20909100
C	41.65955500	77.76753900	39.57314900
C	42.66456300	77.80216000	38.59212500
O	43.26974400	76.59100300	38.30022100
C	44.50289000	76.61124100	37.50760300
C	43.00966000	79.02855600	38.00434500
C	42.36504900	80.20235200	38.40991300
C	41.36945100	80.17978300	39.39280700
C	41.02515200	78.95340900	39.97132000
O	40.07147300	78.78069800	40.97736400
C	39.60811700	79.96593200	41.69919500
H	43.79882300	79.07681000	37.26609500
H	42.65967600	81.15023000	37.97313400
H	40.91942500	81.10121300	39.73774800
H	40.69716700	76.64687600	40.92565800
H	45.28028900	77.19290900	38.01569100
H	44.80382900	75.56582100	37.44652900
H	44.31119300	76.99693300	36.49975100
H	39.03633000	79.57601300	42.54168300
H	40.45170800	80.56697400	42.05377200
H	38.95858400	80.57565400	41.06034800
O	41.96508500	82.08402800	42.51954800
H	54.41380500	85.22104700	38.33218100
H	50.52389300	84.38756100	40.37476500
H	51.64709000	88.42565600	33.30519500
H	36.49452000	80.87935400	35.71431900
H	50.03052400	82.01793600	42.01174800
H	49.17956800	81.45111700	43.47229300
H	50.60676000	80.57475400	42.87530800
H	45.53762900	85.06792500	40.90396100
H	44.56335300	82.18453500	46.51114100
H	38.25050600	82.71284500	45.67655500
H	39.26290800	84.91337100	39.76478900
H	47.89537400	81.63673300	37.82308000
H	38.17877100	73.54377500	40.84916100

H	36.94890100	75.73986000	44.81304800
H	40.48418400	72.81012500	46.23819700
H	53.90832900	81.31050200	38.48208300
H	51.08774100	78.82983000	39.81847800
H	46.98534600	76.14178800	46.92675700

$^{4}TS_{HA,A}$

C	38.74285100	80.31998800	35.07863900
C	39.30596200	79.09229100	34.33086500
C	38.76587900	80.18454800	36.61072600
C	40.81726200	78.87904000	34.52242800
H	39.31439400	81.21224700	34.77997200
H	39.09324300	79.20413700	33.25772900
H	38.76265500	78.19334400	34.65999400
H	38.33533800	81.07182300	37.09227700
H	38.18178300	79.31281500	36.93426300
H	39.78394300	80.06133300	36.99552800
H	41.16872500	78.01404600	33.94764800
H	41.07897000	78.70517700	35.57173500
H	41.38116100	79.75646500	34.18019600
H	50.90156500	90.57243000	30.32696400
C	50.39970000	89.91089500	29.61013000
C	49.07066200	89.42222200	30.14656000
C	48.99000400	88.81503500	31.41296900
C	47.89275400	89.55604500	29.39294400
C	47.76720300	88.35353500	31.91099900
C	46.66605400	89.09494100	29.88750500
C	46.59984600	88.49205000	31.14848600
H	51.08423300	89.07504400	29.41332300
H	50.27378100	90.46396400	28.67299000
H	49.87528600	88.69454100	32.02949700
H	47.93718100	90.02505000	28.41288000
H	47.74942500	87.89458600	32.89338000
H	45.76643100	89.20877500	29.28882000
H	45.64926200	88.13541400	31.53431400
C	38.25406600	75.35584000	41.68435300
C	38.62977800	76.26752200	42.83277500
O	39.77276000	76.78939900	42.95971300
C	37.26158500	75.99442900	40.66685400
C	37.89367400	77.19997200	39.94746700
C	36.79607500	74.92725300	39.65792900
H	39.17975100	75.08600100	41.16710100
H	36.37614700	76.34472100	41.22334200
H	36.30272900	74.08433900	40.15762700
H	37.64916600	74.53383700	39.09174100
H	36.08875600	75.35787100	38.94020100
H	38.24312800	77.96775200	40.64474100
H	38.76090700	76.88230600	39.35677700
H	37.17060600	77.66233700	39.26563600
N	37.67205000	76.51572000	43.77887500
C	37.89570300	77.51094500	44.83187800
C	38.94213600	77.12644200	45.88224800
O	39.43921100	78.01168700	46.60958800
H	36.74322700	76.14195000	43.64669100
H	38.24163400	78.45225600	44.39560300
N	39.25614000	75.80160700	45.97535800
C	40.27285100	75.29753100	46.89057100
C	41.46706400	74.61481900	46.21666200
O	42.12250300	73.76595500	46.86022600
H	38.78187600	75.15101700	45.36600700
H	40.63135600	76.15573000	47.46916900
N	41.76709200	75.00384900	44.94040400
C	42.91713500	74.41867200	44.21392600
C	43.89140200	75.55577800	43.86419700
O	44.89109100	75.78715400	44.60642200
C	42.45154900	73.53099800	43.03473800
C	43.48653100	72.47664500	42.56519900
C	42.84345100	71.55992900	41.50480300
C	44.78963900	73.09536500	42.02568300
H	41.17298600	75.67222800	44.45977400
H	43.43519800	73.80171100	44.95219700
H	41.55605200	72.99836100	43.38340800
H	42.13766800	74.14226700	42.17633900
H	43.73885500	71.85703800	43.44082900
H	45.48182800	72.31710200	41.68557100
H	45.31908000	73.67204700	42.79036000
H	44.58432300	73.76534400	41.18236700
H	41.93440600	71.07720800	41.88685700
H	42.57241000	72.13603100	40.61010100
H	43.54209200	70.77304600	41.19833900
N	43.58730800	76.33390000	42.80001800
C	44.46915300	77.42241600	42.36442600
C	44.69710700	78.49519800	43.42015600
O	45.73853000	79.19921400	43.41528100
H	42.82917100	76.09952000	42.15642500
H	45.44094400	77.04100000	42.03507300
H	44.00918100	77.88879300	41.49002400
N	43.72806900	78.64754300	44.36115000
C	43.83640500	79.62170400	45.44973800
C	45.16147800	79.48524500	46.21987400
O	45.67256300	80.50509800	46.74986600
C	42.63601400	79.45938600	46.39753300
H	42.88177000	78.10221900	44.27947200
H	43.85196600	80.63310400	45.03311900
H	42.60500400	78.44893900	46.82042900
H	42.71434000	80.17194300	47.22168500
H	41.69067700	79.64267600	45.87729900
N	45.66823900	78.22672200	46.34442300
C	46.97711500	77.97189500	46.93647700
C	48.09162200	77.52446800	45.97594700
O	49.28647600	77.68150300	46.32116300
H	45.18406100	77.45500800	45.89400800
H	47.31374000	78.89835600	47.40416900
N	47.72789100	76.91130600	44.81297600
C	48.72831100	76.36190300	43.89056500
C	49.36595500	77.40885200	42.96251100
O	50.50152300	77.16476800	42.45849500
C	48.06834600	75.25014300	43.02804500
C	49.08970800	74.36889300	42.27568300
C	48.45364200	73.46160700	41.22898200
O	48.50387600	72.20628900	41.32147700
N	47.84799000	74.08639400	40.17983600
H	46.75157400	76.70640600	44.62424300
H	49.55143600	75.95141200	44.48343800
H	47.46143800	74.62119100	43.69083300
H	47.39564700	75.72751200	42.30633500
H	49.61656700	73.71823800	42.97957600
H	49.83356100	75.01613600	41.79778800
H	47.68584600	75.10340400	40.09947300
H	47.44471100	73.48678700	39.47414000
N	48.63800600	78.50266700	42.63970800
C	49.13936400	79.45974900	41.63525800
C	50.06311000	80.49577200	42.30239100
C	47.99546600	80.12187400	40.84388600
C	46.93294300	79.16183300	40.26383800
C	47.47796400	77.89450300	39.59656900
O	48.32226000	78.02970300	38.63579300
O	47.02645000	76.76172900	40.03826100
H	47.72907000	78.67216900	43.06569400
H	49.73409100	78.86631100	40.93602700
H	47.47792100	80.84932700	41.48307400
H	48.46646400	80.68314100	40.02888100
H	46.25138100	78.87198100	41.05891100
H	46.34468600	79.71425500	39.51695300
C	48.31209000	80.33914400	36.41940700
C	48.74293100	81.60127200	37.14077500
O	48.10752600	82.69308500	37.11461000
C	47.06216500	80.47854600	35.53054800
C	46.57634100	79.06593800	35.12039800
C	47.32682800	81.38244400	34.30953800
C	45.22723900	79.03781500	34.38378600
H	49.15994400	79.98120000	35.81362500
H	46.27185600	80.94818700	36.13557500
H	47.34687800	78.59699100	34.48794100
H	46.51107200	78.44778900	36.02664000
H	47.66487100	82.37037200	34.63627500
H	46.42466200	81.52024100	33.70236500
H	48.10025400	80.94122900	33.66461900
H	44.91522100	78.00681800	34.17668400
H	44.43839800	79.50813600	34.98558300

H	45.27230200	79.56641200	33.42452600
N	49.90016200	81.46359500	37.86104000
C	50.47437900	82.56012700	38.62119300
C	51.56323800	83.32928700	37.88292400
O	51.71709400	84.57444700	38.07154600
H	50.38685600	80.56998400	37.87184600
H	49.69077500	83.27845600	38.86378500
N	52.34549600	82.63190700	37.02539200
C	53.45436000	83.26189800	36.31208800
C	53.06994100	84.30283800	35.25291200
O	53.96633200	85.00627700	34.73497100
H	52.26912600	81.60994000	37.01779400
H	54.11458900	83.78688100	37.01110600
N	51.74554000	84.41709500	34.94872200
C	51.21392600	85.47606800	34.08814100
C	50.55285800	86.62027600	34.89215300
O	49.86641000	87.48719500	34.28738200
C	50.23888800	84.90967000	33.04572000
H	51.09283400	83.76707400	35.36595700
H	52.08327600	85.91389400	33.58332200
H	49.81127300	85.72543900	32.45941200
H	50.75983100	84.21550300	32.37939000
H	49.41370800	84.37314900	33.53066600
N	50.78111600	86.64611200	36.22804400
C	50.22222300	87.68006800	37.10416900
C	49.20704200	87.14802300	38.13781700
O	49.85605100	86.41687400	39.22035100
C	48.07563000	86.32841600	37.51099600
H	51.34242900	85.91859200	36.66220200
H	49.73794500	88.41318200	36.45345300
H	48.78337900	88.02853500	38.63587000
H	50.46017800	85.71630500	38.87198700
H	47.31845600	86.10888700	38.27037800
H	47.60156400	86.87868700	36.68869700
H	48.44059500	85.37279900	37.12131600
H	40.81702000	86.55653600	40.05229800
C	41.75581700	86.93088300	40.46944200
S	42.07167900	86.09519400	42.12606800
H	41.66752600	88.00356100	40.65633800
H	42.57233400	86.72350700	39.77775400
C	52.99779800	78.89755800	37.93290200
O	52.17220200	79.80068300	37.61251000
N	52.72576500	77.77348700	38.60711800
C	51.39764200	77.32301300	39.07594800
C	51.10763400	75.87509200	38.62754200
C	51.02772600	75.70141300	37.12295800
C	49.92712300	76.22436200	36.41591500
C	52.03753600	75.03767900	36.40817100
C	49.85186800	76.08377600	35.02654100
C	51.96111600	74.89750700	35.01618300
C	50.86692500	75.42250500	34.32076800
H	50.63459700	77.99039800	38.67369000
H	51.87003300	75.20060400	39.04435200
H	50.15347100	75.59398300	39.08655900
H	49.14726300	76.73879600	36.97467000
H	52.88335200	74.61489400	36.94617000
H	48.99619600	76.48841000	34.49276800
H	52.75022500	74.37714200	34.48013400
H	50.80268600	75.31469000	33.24160400
H	53.50398400	77.15391500	38.79502200
C	40.30409700	83.92333600	38.99305600
C	45.08760500	83.94397200	39.83212900
C	44.25161300	83.35863700	44.56477700
C	39.47254800	83.71150400	43.77046500
C	41.67981900	83.92505500	38.80638900
C	42.33390900	83.99576400	37.50784500
C	43.68939000	84.02954800	37.74393400
C	43.85875300	83.96592300	39.19042300
C	44.81221800	84.13453100	36.75558900
C	41.61275500	84.03716100	36.19359200
H	40.99711800	84.94162400	36.09306300
C	45.27705400	83.79227300	41.19671500
C	46.58300000	83.73094400	41.84116300
C	46.34583400	83.49875300	43.18064300
C	44.89180100	83.46107800	43.34260600
C	47.88883800	83.84857700	41.12015700
C	47.28558400	83.26669500	44.27637200
C	48.49320100	83.84355700	44.41724100
C	42.88080100	83.44313200	44.76870800
C	42.24613900	83.37347800	46.07098900
C	40.88323700	83.48758100	45.85106300
C	40.70192500	83.60952800	44.40699900
C	42.97547700	83.16946800	47.36471000
C	39.78564200	83.45733300	46.81188500
C	39.82310300	83.85971900	48.09599400
C	39.27539100	83.80424200	42.40292400
C	37.97630700	83.90843500	41.75498600
C	38.20931800	83.96081300	40.39999500
C	39.65266800	83.89429300	40.21981900
C	36.66717300	83.95562900	42.48503000
C	37.21925700	84.07430700	39.27934700
H	37.38395400	84.98065100	38.68173400
N	42.62635000	83.90998200	39.82016900
N	44.26392800	83.63598200	42.12741700
N	41.93149700	83.60194100	43.77131500
N	40.28472400	83.80116100	41.44901900
Fe	42.29350100	83.63600900	41.78715900
H	40.94436200	83.17503100	36.06733300
H	42.31720400	84.03021600	35.35731200
H	46.96562100	82.54387200	45.02756100
H	38.84182700	83.06158800	46.43707600
H	36.19265700	84.11394100	39.65568400
H	37.28226600	83.22050000	38.59084100
H	48.86567800	84.59474400	43.72789900
H	49.14014600	83.58442000	45.24926900
H	40.70540900	84.30298700	48.54419000
H	38.94983500	83.77091900	48.73471500
H	35.82463000	84.02994800	41.79103200
H	36.61205600	84.81884900	43.16132400
H	36.50903700	83.05751100	43.09733200
H	44.46387700	83.93386500	35.73763300
H	45.25247800	85.14106600	36.75430400
H	45.63228800	83.43858500	36.96661300
H	42.31931500	82.70426200	48.10759500
H	43.85385800	82.52602100	47.24211200
H	43.32375400	84.12040700	47.79251800
H	48.68918700	83.38112000	41.70101300
H	48.18528900	84.88965000	40.93126700
H	47.84548300	83.35058100	40.14574200
O	41.47261500	76.57639500	40.89041700
C	41.97227000	77.45927800	39.93403000
C	42.93762200	76.99687900	39.02329500
O	43.29405700	75.66338000	39.13916500
C	44.50424800	75.21861300	38.44106400
C	43.48813400	77.89019100	38.09299900
C	43.09000800	79.23221000	38.09131700
C	42.14546400	79.70847900	39.00546000
C	41.59565400	78.81116100	39.92752300
O	40.68267500	79.15169500	40.92661700
C	40.28988100	80.52609200	41.09131500
H	44.24869500	77.55536000	37.40053700
H	43.54632500	79.92230300	37.39035900
H	41.90099700	80.76100000	39.04600100
H	40.82717000	76.99569400	41.51694600
H	45.37333100	75.79884200	38.77147300
H	44.61944300	74.17506700	38.73255600
H	44.37196400	75.28319600	37.35473400
H	39.59387700	80.54994800	41.92844200
H	41.30683400	81.25009900	41.39867300
H	39.86726900	80.94640000	40.17265500
O	42.27246300	81.90773100	41.63957900
H	54.03746300	82.47542900	35.82530900
H	50.89270900	82.17846900	39.55972700
H	51.03808900	88.17449800	37.64470000
H	37.70792000	80.49352900	34.74910800
H	50.48263100	81.18231600	41.55524900
H	49.51435400	81.08930000	43.04224400
H	50.89138400	79.98785500	42.80579800
H	45.97192700	84.01515700	39.20988400
H	44.87545400	83.22065500	45.43851200

H	38.58871400	83.72990900	44.39698500
H	39.68550000	83.96805000	38.10364200
H	48.15998400	79.56182700	37.18905300
H	37.81130100	74.43469500	42.08932600
H	36.94430400	77.69745300	45.33881200
H	39.85178600	74.57194700	47.59241700
H	54.05674600	78.99982700	37.65938800
H	51.35977000	77.38166800	40.17060400
H	46.89226100	77.20873100	47.72120400

^{4}IM1$_{HA,A}$

C	39.00606400	81.58358700	35.63437700
C	39.85139600	80.69840400	34.69358300
C	38.99195300	81.10922800	37.09741700
C	41.36160800	80.73673000	34.98383000
H	39.38557600	82.61582300	35.58793800
H	39.67789900	81.02045600	33.65665800
H	39.49029200	79.66059900	34.75798700
H	38.36296300	81.76061000	37.71731800
H	38.59460400	80.08823200	37.17316600
H	39.99688500	81.10627500	37.53316900
H	41.91661300	80.12731700	34.26099500
H	41.59761900	80.35674800	35.98390300
H	41.74562000	81.76286900	34.91798800
H	50.84483100	89.26826900	29.23069600
C	50.68500100	88.46444600	28.50143700
C	49.42286300	87.68522600	28.80632700
C	49.21159800	87.13977500	30.08561000
C	48.44166900	87.48233400	27.82142700
C	48.05346400	86.41001100	30.37180000
C	47.28038800	86.75170500	28.10364000
C	47.08341400	86.21315800	29.38019400
H	51.57244000	87.81873700	28.53862800
H	50.64516800	88.91724700	27.50463500
H	49.94251500	87.27411100	30.87676100
H	48.58908900	87.89922800	26.82781100
H	47.93163000	86.00983900	31.37216100
H	46.53331900	86.60561300	27.32800200
H	46.18339500	85.64627600	29.60061900
C	38.33932500	75.17375600	40.86580900
C	38.59898800	75.94257100	42.14254700
O	39.70711900	76.48891900	42.40835300
C	37.40142500	75.91004600	39.86254800
C	38.01180600	77.24539700	39.39846100
C	37.10372800	74.98770800	38.66510800
H	39.30867200	74.99741500	40.38957700
H	36.44790400	76.12131300	40.37479700
H	36.63905100	74.04685100	38.98460600
H	38.02750100	74.74405200	38.12640700
H	36.42444000	75.47893700	37.95962900
H	38.19924000	77.92912400	40.23285000
H	38.96980500	77.07464100	38.89374300
H	37.34064300	77.74921200	38.69372200
N	37.57632600	76.03572800	43.04565700
C	37.68741300	76.90947900	44.21883000
C	38.72545200	76.47474000	45.25821000
O	39.18199500	77.31300200	46.06313500
H	36.67570000	75.64457300	42.80902900
H	37.97458300	77.91996100	43.91516200
N	39.07285600	75.15481300	45.25650300
C	40.07363100	74.60486500	46.16303500
C	41.32597800	74.04709200	45.48040400
O	41.98777100	73.15781200	46.05881200
H	38.63608000	74.54202700	44.58324700
H	40.36599300	75.41383600	46.84119400
N	41.66886000	74.58883900	44.27141500
C	42.87361300	74.12734200	43.54600200
C	43.84730500	75.31009900	43.43381700
O	44.79546300	75.43295300	44.26259900
C	42.50370300	73.41471100	42.22363100
C	43.60116700	72.47541700	41.65950900
C	43.02481500	71.65235600	40.48938900
C	44.87627800	73.21853900	41.21737000
H	41.06965200	75.28360600	43.83662100
H	43.35555200	73.41546700	44.22081900
H	41.61420800	72.80795800	42.44234200
H	42.20710000	74.13814400	41.45020200
H	43.87412200	71.77407800	42.46401600
H	45.61523200	72.52147400	40.80731500
H	45.36547800	73.73112500	42.05123700
H	44.64138200	73.96736400	40.45083100
H	42.14150500	71.07776400	40.79721100
H	42.73156300	72.31047900	39.66061400
H	43.77222900	70.94757400	40.10803800
N	43.60473900	76.24419700	42.48197900
C	44.51991700	77.37227500	42.28059500
C	44.65267800	78.30000500	43.48083300
O	45.67467900	79.01676200	43.62625600
H	42.90438500	76.09634800	41.75407800
H	45.52205500	77.02410300	42.01305400
H	44.15157300	77.95393200	41.43370400
N	43.62952300	78.31751100	44.37452500
C	43.68562300	79.13257200	45.59203600
C	44.92643300	78.81505100	46.44955300
O	45.40336300	79.70972400	47.18921300
C	42.39289600	78.94296700	46.40172200
H	42.80835100	77.75777300	44.19305900
H	43.80045500	80.18454200	45.31228000
H	42.26100800	77.89586000	46.69697000
H	42.43369800	79.55214600	47.30772200
H	41.51555900	79.24800700	45.82236700
N	45.40403000	77.53947600	46.39846800
C	46.65383700	77.15981900	47.05014900
C	47.85562900	76.91967500	46.12129700
O	49.01610300	77.03258500	46.57905700
H	44.95543300	76.86307400	45.78813100
H	46.93292700	77.96624800	47.72960500
N	47.59443600	76.52506000	44.84084900
C	48.66879500	76.15259200	43.91397900
C	49.34977500	77.34867400	43.22622000
O	50.52246400	77.20474100	42.77634700
C	48.09269800	75.19473700	42.83443000
C	49.17860800	74.41933100	42.05696700
C	48.62452200	73.68215900	40.84303300
O	48.57610300	72.42468900	40.79458800
N	48.18840300	74.46445200	39.81635300
H	46.64045900	76.32957700	44.55504800
H	49.45739700	75.65396000	44.48609000
H	47.42525400	74.48166600	43.33386200
H	47.49232700	75.78142400	42.12887500
H	49.63681400	73.66621600	42.70433400
H	49.96228100	75.11853100	41.74667600
H	48.11249300	75.49000900	39.84176200
H	47.84385100	73.98477000	38.99737800
N	48.61935400	78.47476300	43.04564100
C	49.15877800	79.61329400	42.27997500
C	49.93516700	80.57164100	43.20166400
C	48.05607500	80.35106400	41.49777300
C	47.16509200	79.47293800	40.59212100
C	47.89261800	78.40956400	39.76225400
O	48.82370100	78.79209100	38.94982500
O	47.50917500	77.18941900	39.91267300
H	47.68152500	78.55271400	43.43279200
H	49.85968900	79.18213300	41.55950600
H	47.40151000	80.88845600	42.19709500
H	48.56068800	81.11554900	40.89538000
H	46.42506300	78.96806800	41.20879000
H	46.62009600	80.13377400	39.90278100
C	48.92626100	79.84993400	35.72923900
C	49.26847100	81.17860200	36.36967600
O	49.45137200	82.23251400	35.67772200
C	47.46276600	79.78046700	35.19837200
C	47.10483000	78.30333000	34.89966200
C	47.25163600	80.69195800	33.97440000
C	45.65104800	78.07398500	34.45325300
H	49.61686600	79.69211500	34.89144500
H	46.79957400	80.12411900	36.00951900
H	47.78827500	77.92611400	34.12280700
H	47.30247000	77.70983400	35.80328700
H	47.59093600	81.70950800	34.18368900

H	46.19676900	80.73092700	33.67996900
H	47.82906600	80.31815200	33.11736500
H	45.44079700	77.00289600	34.34354600
H	44.94283000	78.48066500	35.18738600
H	45.43905300	78.55107000	33.49002100
N	49.35259000	81.18860400	37.72028000
C	49.54756000	82.43341600	38.45733000
C	50.69646100	83.30399400	37.96426000
O	50.56003700	84.56960700	37.93918300
H	49.18001100	80.31151500	38.25627700
H	48.65271200	83.06442600	38.42798800
N	51.84365700	82.69882100	37.61378000
C	53.00642100	83.44424300	37.14172300
C	52.84477700	84.19197700	35.81168500
O	53.74629100	84.99264100	35.46036300
H	51.96187200	81.68862200	37.72501100
H	53.30270800	84.20435400	37.87246500
N	51.71320000	83.95797500	35.09529500
C	51.37334000	84.72673300	33.89747400
C	50.24837600	85.75124700	34.16756300
O	49.65352700	86.30862000	33.20468200
C	50.98857200	83.79674600	32.73627600
H	51.02618800	83.26492600	35.40074700
H	52.26938400	85.30318800	33.63727600
H	50.63349600	84.38694800	31.88851800
H	51.85519300	83.20211700	32.43053300
H	50.19122400	83.11321200	33.04832700
N	49.97335800	86.02427200	35.46753200
C	48.90155300	86.93286600	35.87268100
C	47.73277600	86.22916800	36.59580800
O	48.08016300	85.85236500	37.96466400
C	47.18333900	85.03078600	35.81663200
H	50.47122100	85.52552000	36.20087900
H	48.53599900	87.41365900	34.96089200
H	46.94283600	86.98060000	36.72153500
H	48.95144500	85.37626200	37.99919300
H	46.32370600	84.60428400	36.34478500
H	46.86560100	85.33021100	34.81030400
H	47.94229500	84.24845500	35.71689000
H	39.81630800	87.04588600	42.03197500
C	40.79239200	87.51620400	42.16190700
S	41.85598700	86.48403400	43.32890000
H	40.67431000	88.49423300	42.63459400
H	41.28751000	87.62319500	41.19600900
C	53.63412000	79.34445100	38.60024000
O	52.81937600	80.12361700	38.05015900
N	53.32151200	78.30335300	39.39720600
C	51.95518500	77.86300500	39.73227200
C	51.72049800	76.38231800	39.36347600
C	51.80476500	76.08395900	37.88095700
C	50.75797200	76.47847300	37.02647700
C	52.91151100	75.42053400	37.32775000
C	50.82483700	76.21507200	35.65508500
C	52.98011000	75.15692500	35.95380600
C	51.93561300	75.55442000	35.11283500
H	51.25323500	78.50273100	39.19498800
H	52.43356500	75.75342000	39.91547000
H	50.72369600	76.12503600	39.73643000
H	49.90304600	76.99548400	37.45553600
H	53.71998500	75.09754500	37.97954600
H	50.00905900	76.52398500	35.00771700
H	53.84348000	74.63982300	35.54425500
H	51.98410500	75.35042800	34.04702900
H	54.08782100	77.76267500	39.77646000
C	39.77941600	84.78411000	39.89672600
C	44.55052700	85.56481300	40.39410000
C	44.37364100	83.48153400	44.77216800
C	39.52123800	83.62353700	44.60697500
C	41.09449000	85.13742000	39.64143600
C	41.56607100	85.69334500	38.38158600
C	42.90216100	85.97545600	38.54419800
C	43.25987800	85.55873200	39.89313300
C	43.86283100	86.57612800	37.56424900
C	40.70850700	85.89323800	37.16798800
H	39.82124700	86.50067600	37.38907800
C	44.92638000	85.02413200	41.61404500
C	46.30178900	84.90563300	42.07166000
C	46.25509300	84.24960000	43.28896600
C	44.84313700	84.00070600	43.57827100
C	47.50587800	85.38169700	41.31952700
C	47.34299600	83.82642300	44.16360300
C	48.56246500	84.38816600	44.26829600
C	43.03722700	83.38735200	45.13308100
C	42.57114200	83.01260900	46.45716600
C	41.18864700	83.09805900	46.42631400
C	40.82178500	83.49895400	45.07148800
C	43.45880200	82.60012400	47.59224100
C	40.21697600	82.81513000	47.47934200
C	40.37689200	83.01101500	48.80106900
C	39.16630000	83.91591700	43.29968100
C	37.80478100	83.89829700	42.78634800
C	37.88193900	84.17253500	41.43907800
C	39.28855900	84.38554700	41.13249800
C	36.58775800	83.62747200	43.62014300
C	36.77258100	84.26867400	40.43415500
H	36.69949100	85.27593400	40.00286600
N	42.14006400	85.07472100	40.55066700
N	44.05863500	84.47235700	42.54296300
N	41.96317200	83.68757100	44.31238200
N	40.05055600	84.21465100	42.27495900
Fe	42.06806700	84.33601200	42.39987700
H	40.35428900	84.93547300	36.76266700
H	41.26063800	86.39872200	36.37075400
H	47.13381300	82.95800800	44.78692800
H	39.26522300	82.40344600	47.14310900
H	35.80291200	84.03673900	40.88491600
H	36.92183900	83.57176300	39.59853900
H	48.85162700	85.27438700	43.71466000
H	49.30778900	83.97238700	44.93857100
H	41.27199200	83.45620000	49.22145500
H	39.59372400	82.74441800	49.50405200
H	35.67914000	83.61998900	43.01045500
H	36.45281900	84.38997500	44.39881800
H	36.64994000	82.65713200	44.13038800
H	43.37205100	86.81658200	36.61688700
H	44.30647800	87.50216800	37.95176100
H	44.69263400	85.89509500	37.34209700
H	42.88182000	82.08024100	48.36256600
H	44.25832500	81.91991800	47.27718200
H	43.93088300	83.46874000	48.07287800
H	48.36345000	84.73019700	41.52185300
H	47.79944000	86.39596400	41.62639200
H	47.35782500	85.41112600	40.23564500
O	41.55010200	76.69151000	40.46759700
C	42.11843800	77.79202900	39.83110900
C	43.20965600	77.58240700	38.96730900
O	43.60686800	76.26693700	38.80391000
C	44.87160900	76.01089900	38.10652200
C	43.83993800	78.68645200	38.37614600
C	43.40793500	79.98541600	38.67258100
C	42.34080600	80.21287600	39.54738900
C	41.70126300	79.10182800	40.10424300
O	40.64482600	79.17639900	41.03199900
C	39.99465200	80.37068700	41.29947100
H	44.69348300	78.54020700	37.72783000
H	43.93408200	80.83230500	38.24704000
H	42.09400200	81.21928200	39.86522200
H	40.83913500	76.92588000	41.12116300
H	45.70765300	76.49863700	38.62075000
H	44.99045400	74.92890800	38.14743200
H	44.80840200	76.33568300	37.06189000
H	39.24927500	80.27009500	42.07425600
H	41.52691500	82.01071000	42.16080700
H	39.94061600	81.12473500	40.52439500
O	42.19224400	82.62757600	41.77841400
H	53.82855200	82.73073400	37.04309900
H	49.75178100	82.17418300	39.50143300
H	49.30598000	87.70455700	36.53869000
H	37.97316500	81.61851100	35.25788000
H	50.36199700	81.39984500	42.62247100

H	49.27385400	80.99602600	43.96592400
H	50.74875700	80.03692500	43.70098800
H	45.33376100	85.95833600	39.75757100
H	45.10422800	83.18880900	45.51477800
H	38.71818800	83.44448200	45.31109900
H	39.06850200	84.88769300	39.08552500
H	49.08207400	79.05210500	36.46033200
H	37.90261600	74.19646400	41.11545600
H	36.70710400	76.96190900	44.70194400
H	39.65779000	73.79341600	46.76668500
H	54.71853400	79.47155600	38.45964100
H	51.78025300	77.98330200	40.80738100
H	46.50383900	76.24915100	47.64414200

$^4P_{hy,A}$

C	38.17795200	82.49708300	35.68758200
C	39.28982900	81.67298900	35.00414800
C	37.69942000	81.91652000	37.02943500
C	40.61424800	81.63049300	35.78487200
H	38.53996600	83.52464000	35.84338400
H	39.47579300	82.09530300	34.00650700
H	38.92636400	80.64699100	34.84026900
H	36.87564800	82.50969800	37.44575700
H	37.33828300	80.88712200	36.90180200
H	38.50497400	81.89985400	37.77221000
H	41.38379700	81.08673700	35.22543300
H	40.50496200	81.12994000	36.75350700
H	40.99237000	82.64358100	35.97263700
H	51.01672700	89.78869200	29.37067300
C	51.39837600	88.92713700	28.80795000
C	50.34476500	87.84856500	28.66768100
C	49.76660700	87.26233000	29.80830400
C	49.92272800	87.40862700	27.40202200
C	48.79474400	86.26410000	29.68688100
C	48.94948400	86.40895300	27.27590800
C	48.38265500	85.83380600	28.41864700
H	52.27916400	88.55804000	29.34906400
H	51.73435900	89.28818800	27.82975800
H	50.06288100	87.57147900	30.80575300
H	50.36012300	87.85160100	26.51029700
H	48.37566400	85.83987800	30.59255100
H	48.63741900	86.08190400	26.28766000
H	47.62873800	85.05771300	28.32285300
C	38.19369400	76.24044400	40.89836100
C	38.64163700	76.97397500	42.14307600
O	39.84471300	77.27768400	42.37602600
C	37.46075000	77.14774800	39.86344600
C	38.39897200	78.22867300	39.29749500
C	36.87193200	76.27736100	38.73714200
H	39.08685000	75.80900300	40.43584800
H	36.62495400	77.64745600	40.38066200
H	36.16334900	75.53537400	39.12537700
H	37.66840600	75.74095800	38.20680200
H	36.34468400	76.90000800	38.00585500
H	38.84768400	78.84614800	40.08192100
H	39.22110600	77.76883200	38.73570000
H	37.85402000	78.89009800	38.61472300
N	37.67895500	77.31221200	43.05632300
C	37.99382100	78.17969300	44.19520100
C	38.88963800	77.55468400	45.26802800
O	39.46410600	78.29560700	46.09594800
H	36.71125000	77.11580000	42.84393500
H	38.51591000	79.07901400	43.85490400
N	38.98687500	76.19504000	45.26953900
C	39.82132700	75.45991200	46.21324100
C	40.95913600	74.64628200	45.58616200
O	41.43666100	73.68374000	46.22480900
H	38.45647500	75.67811500	44.58290800
H	40.25221700	76.19656500	46.89953500
N	41.41657400	75.05395600	44.36263900
C	42.53618200	74.35885400	43.68246900
C	43.70393000	75.35310400	43.58649300
O	44.70821400	75.25294500	44.34886000
C	42.07430100	73.72842100	42.34730700
C	43.01531100	72.64003000	41.77067800
C	42.35217500	71.99475700	40.53677000
C	44.41554300	73.17256100	41.41292500
H	40.95772400	75.82049600	43.88010900
H	42.85859700	73.57696500	44.37366100
H	41.09621900	73.26875000	42.54673600
H	41.90980300	74.50403600	41.58529200
H	43.12983100	71.86112900	42.54167200
H	45.03809500	72.39215500	40.96292800
H	44.95949200	73.52148600	42.29578600
H	44.33986300	74.00632800	40.70308200
H	41.36677500	71.57557000	40.77926900
H	42.21989300	72.73801200	39.73928900
H	42.97723700	71.18569100	40.14245900
N	43.54290500	76.39568000	42.73455600
C	44.53762100	77.46072900	42.63012800
C	44.78477300	78.23512000	43.92056200
O	45.83366400	78.91713000	44.04775100
H	42.83534000	76.38348200	41.99316000
H	45.50804200	77.06669700	42.31589900
H	44.20487200	78.15739100	41.85635500
N	43.85698600	78.13328500	44.90525400
C	44.06946000	78.70506400	46.24042900
C	45.40338800	78.25407500	46.86700000
O	46.00328700	79.02475400	47.65058500
C	42.89517800	78.32145200	47.15613600
H	43.01472100	77.60328500	44.73138200
H	44.14216300	79.79196300	46.15892800
H	42.85311500	77.23341700	47.29133100
H	43.03354300	78.78010600	48.13832700
H	41.93997600	78.66720700	46.74877000
N	45.80779400	76.98048400	46.58545500
C	47.08798000	76.46207800	47.05515700
C	48.16844000	76.24959300	45.98483800
O	49.37474400	76.26135600	46.31865500
H	45.24883600	76.40360000	45.96330100
H	47.48948800	77.17671200	47.77521600
N	47.75106200	75.98644400	44.70883700
C	48.70289100	75.65784900	43.63978500
C	49.25289200	76.88332000	42.88642500
O	50.34143200	76.76939200	42.26084700
C	48.00592600	74.68953600	42.64600200
C	48.88851900	74.19695400	41.47844100
C	48.06237900	73.36629300	40.50641600
O	47.67415300	72.20095200	40.78840900
N	47.74009600	73.98564500	39.33978800
H	46.76155200	75.86283500	44.51698500
H	49.57231000	75.17587400	44.09576900
H	47.64316400	73.82201200	43.20936400
H	47.12082000	75.19514400	42.23341900
H	49.69047000	73.56202500	41.87046800
H	49.35669700	75.04026100	40.96807800
H	47.97153500	74.96360800	39.12469300
H	47.19558000	73.45875100	38.67253800
N	48.47332200	77.99215900	42.84018300
C	48.79065300	79.15057000	41.98368200
C	49.15164200	80.37139000	42.84642800
C	47.61295800	79.45525200	41.02989800
C	47.19392600	78.28540800	40.12773600
C	48.18763400	77.90066600	39.02065800
O	48.95028400	78.82144700	38.54249300
O	48.16638900	76.68256700	38.60217100
H	47.66009900	78.08124100	43.44491300
H	49.65932300	78.85247900	41.39059000
H	46.75468900	79.78160500	41.63224400
H	47.90764600	80.29992300	40.39603700
H	47.00062900	77.38507800	40.71911900
H	46.24783200	78.54067600	39.62735400
C	49.85937400	79.90178400	35.37302300
C	49.39733300	81.22618400	35.94788500
O	49.40773400	82.29967100	35.26712200
C	48.70538300	78.88279700	35.15294800
C	49.28099400	77.50959900	34.72768600
C	47.67448100	79.41884000	34.14150800
C	48.29133500	76.34804400	34.92240600
H	50.36196500	80.11602200	34.42393000

H	48.21916000	78.73931000	36.12570600
H	49.61172200	77.56286300	33.67818500
H	50.17390600	77.29844200	35.33117200
H	47.24953600	80.37529500	34.46712400
H	46.84673700	78.71306500	34.00566000
H	48.14190300	79.58349000	33.16139200
H	48.73006100	75.39918500	34.58838600
H	48.04282900	76.25428900	35.98648100
H	47.36459000	76.49895100	34.35391500
N	48.96739500	81.20699800	37.23504000
C	48.38699700	82.39419800	37.84666100
C	49.30135400	83.61382100	37.86021400
O	48.79043800	84.78186400	37.80203300
H	48.95929400	80.31988200	37.77153300
H	47.47495600	82.71120900	37.32876700
N	50.62488300	83.41424900	37.94106200
C	51.59112300	84.50777800	37.89662400
C	51.66635600	85.27763500	36.57165000
O	52.32265800	86.34673200	36.52846000
H	51.01833400	82.47195500	37.98944600
H	51.36862700	85.25298800	38.66613600
N	50.99673800	84.75388800	35.50878200
C	50.88185500	85.45829100	34.23186900
C	49.49471600	86.11293000	34.05629200
O	49.13819500	86.55733900	32.93120800
C	51.18932000	84.51635900	33.05685200
H	50.51692300	83.85428500	35.57490200
H	51.61188200	86.27624900	34.26185800
H	50.99020300	85.02314000	32.11003800
H	52.23917100	84.20817500	33.09098300
H	50.56254100	83.61981400	33.11708600
N	48.72040700	86.20379500	35.16769600
C	47.37106400	86.76453300	35.14199400
C	46.26945200	85.73746700	35.47607100
O	46.24032800	85.41446500	36.90250800
C	46.34281500	84.46733900	34.62403400
H	49.05792200	85.81815200	36.04509900
H	47.21601900	87.16204000	34.13486300
H	45.30811100	86.23686300	35.30227000
H	47.13441900	85.13307000	37.23241800
H	45.49896900	83.81275500	34.86628300
H	46.30261900	84.71226600	33.55566600
H	47.27187500	83.91746600	34.80863900
H	39.52536200	86.89492200	42.66688700
C	40.45542600	87.44819200	42.81292300
S	41.67798900	86.41190500	43.82121800
H	40.25443700	88.36082700	43.37907400
H	40.88782700	87.70519900	41.84382700
C	53.19470600	80.42229400	37.60789000
O	52.53292000	81.41296100	38.01234900
N	53.08047500	79.16966900	38.08054100
C	52.13485400	78.76791800	39.14364300
C	52.55505400	77.41888400	39.75964900
C	52.66009300	76.30438200	38.73160600
C	51.50934100	75.79644100	38.09441900
C	53.91341500	75.77300200	38.37609200
C	51.62115900	74.78180300	37.13782700
C	54.02386700	74.75916900	37.41422300
C	52.87566700	74.26027400	36.79207900
H	51.11841900	78.70399400	38.73744200
H	53.52076900	77.53702200	40.26896400
H	51.81681500	77.16467100	40.53028800
H	50.52471600	76.18770000	38.33975900
H	54.80723100	76.14225700	38.87435900
H	50.72167600	74.39501700	36.66867100
H	55.00155500	74.35890600	37.16038500
H	52.95439600	73.47066000	36.05046000
H	53.61885000	78.43093100	37.64390100
C	39.42045600	84.83662100	40.35072900
C	44.23690100	85.45578400	40.28482200
C	44.56118500	83.47612500	44.70412400
C	39.77826800	82.62041100	44.66140200
C	40.69897600	85.21639300	39.97482700
C	41.02345800	85.87691600	38.72138800
C	42.38828300	86.05084900	38.70136700
C	42.89622700	85.49618300	39.94720000
C	43.24148300	86.69184400	37.65020700
C	40.01830700	86.27747200	37.68382000
H	39.31977700	87.03507500	38.06444200
C	44.74493200	84.94229400	41.46766500
C	46.15604300	84.92679900	41.80707300
C	46.25133400	84.36937500	43.06985700
C	44.89096400	84.03686600	43.48190500
C	47.26206100	85.38915200	40.91067100
C	47.43810600	84.07881500	43.86775300
C	48.60785000	84.74457100	43.83996100
C	43.28635800	83.09490200	45.09066400
C	42.95928200	82.50517400	46.37208500
C	41.60350100	82.21502500	46.34010200
C	41.10942700	82.66153500	45.04749300
C	43.93901900	82.26101500	47.48021700
C	40.77174200	81.55110000	47.34533100
C	40.85850700	81.71226600	48.67787900
C	39.26812300	83.15212700	43.48790100
C	37.86418300	83.11840200	43.11381700
C	37.75954500	83.75364100	41.89646800
C	39.10020800	84.17303600	41.52538700
C	36.77586500	82.51725800	43.95276600
C	36.53236200	84.00588300	41.07204600
H	36.33483800	85.08013000	40.95924800
N	41.85157700	85.00177600	40.72101400
N	43.98640700	84.39093600	42.49349100
N	42.14874100	83.19004200	44.28851100
N	40.01088400	83.80112300	42.50656200
Fe	41.97478000	84.29322600	42.59321600
H	39.41578200	85.42328700	37.34672000
H	40.50888100	86.70023500	36.80269300
H	47.35707200	83.22878400	44.54360700
H	40.03340700	80.84764800	46.95904200
H	35.64398800	83.55867400	41.52817200
H	36.62813700	83.58924000	40.06060800
H	48.76831800	85.62350000	43.22541800
H	49.44118800	84.42967600	44.45962300
H	41.54732600	82.41424700	49.13669900
H	40.21878000	81.15089300	49.35189700
H	35.81186800	82.54216900	43.43562900
H	36.65305400	83.05645000	44.90133300
H	36.98710600	81.46992600	44.20804900
H	42.69962000	86.78699300	36.70365300
H	43.54964200	87.70322900	37.95106200
H	44.15966400	86.12178700	37.46245200
H	43.54450000	81.53826900	48.19910400
H	44.89412700	81.86480200	47.11889900
H	44.15666000	83.18567400	48.03240100
H	48.18120900	84.82900600	41.11111400
H	47.49627200	86.45225300	41.06228900
H	47.02446500	85.26230700	39.85137900
O	41.81288200	76.99060600	40.58031300
C	42.44289400	77.86073000	39.68916700
C	43.35075200	77.34558300	38.74738600
O	43.55936800	75.97547800	38.77792000
C	44.63126200	75.43137000	37.94435600
C	43.99395000	78.22248100	37.86179300
C	43.75541200	79.59887400	37.94245100
C	42.88334200	80.12608400	38.89904800
C	42.23263100	79.24433900	39.76824500
O	41.33601600	79.62101700	40.78423300
C	41.14888700	81.03505500	41.06465600
H	44.69945000	77.84339000	37.13464300
H	44.27630600	80.26991400	37.26876500
H	42.76759000	81.19648200	39.00028700
H	41.09696300	77.42158700	41.11712200
H	45.59272400	75.90251000	38.17599300
H	44.66644000	74.37324500	38.19856000
H	44.39555000	75.55196800	36.88081000
H	40.32057800	81.05299500	41.77188900
H	42.41936100	81.55549900	42.54429000
H	40.89554000	81.56665100	40.14681700
O	42.32368700	81.65337200	41.57276400
H	52.57173200	84.07431400	38.10530200

H	48.11537600	82.14049900	38.87691700
H	47.30061400	87.59146900	35.85921100
H	37.32014300	82.57319300	35.00401100
H	49.39595600	81.23411300	42.21520500
H	48.30933000	80.64712900	43.49227400
H	50.01516600	80.15015000	43.48215100
H	44.94178900	85.84742700	39.56181400
H	45.36173000	83.31342300	45.41385500
H	39.07566100	82.17614200	45.35601700
H	38.61037000	85.06868100	39.66940400
H	50.58463400	79.45132400	36.06069600
H	37.52888800	75.41379800	41.18584400
H	37.05467200	78.48763700	44.66451400
H	39.22499700	74.76001400	46.80563300
H	53.94906500	80.53323700	36.81473000
H	52.13638900	79.55290300	39.90657800
H	46.93901300	75.50525400	47.57301800

$^{2}\mathrm{Re_A}$

C	36.50465100	81.68575200	35.53008600
C	36.44790000	80.47209800	34.57765500
C	36.62763700	81.31296600	37.01732200
C	37.73783000	79.63507500	34.54795400
H	37.35130700	82.32669300	35.24268500
H	36.23147000	80.83157000	33.56150700
H	35.59984800	79.83117800	34.86377200
H	36.61468100	82.20785100	37.65122100
H	35.79371700	80.67096700	37.33080800
H	37.56038800	80.77698600	37.22618600
H	37.66095900	78.81753900	33.82151500
H	37.95689800	79.18878700	35.52468100
H	38.59976300	80.25249000	34.26522500
H	51.76092900	77.90777300	28.53437300
C	50.94057500	77.92750000	29.26600200
C	50.27479700	79.28697300	29.31427200
C	50.89965700	80.37146500	29.95833500
C	49.02778500	79.50288200	28.70497400
C	50.30210400	81.63706300	29.99464300
C	48.42357800	80.76610900	28.73451800
C	49.05828300	81.83335800	29.37870500
H	51.36875300	77.65590900	30.23818500
H	50.22867100	77.14428000	28.98270800
H	51.86406000	80.22086800	30.43767800
H	48.52695800	78.67532100	28.20838800
H	50.78824400	82.46332100	30.50614100
H	47.45696600	80.91182700	28.26010200
H	48.59053900	82.81271900	29.41015600
C	38.67399100	75.16680700	39.96141000
C	38.94189800	75.76513200	41.32507300
O	40.04458100	76.28310500	41.65496500
C	37.78879600	76.07210400	39.05082400
C	38.49697900	77.39530900	38.70660000
C	37.40161000	75.30107000	37.77459400
H	39.64214700	74.99651100	39.48039700
H	36.86276200	76.30632700	39.60193900
H	36.84866400	74.38275600	38.00823800
H	38.29750500	75.02197200	37.20648100
H	36.77263800	75.92036200	37.12506000
H	38.82577000	77.93957100	39.59746100
H	39.38803200	77.20721800	38.09587500
H	37.82764600	78.04883100	38.13479300
N	37.91613000	75.73577900	42.23313700
C	38.02049400	76.44883700	43.50859100
C	38.96507600	75.82204000	44.53817200
O	39.34041400	76.49903200	45.51934500
H	37.01860300	75.37321900	41.94567200
H	38.38798900	77.46524700	43.34142700
N	39.32553500	74.52294800	44.32943300
C	40.23107400	73.80574400	45.21991700
C	41.56739000	73.38503400	44.59885000
O	42.20610300	72.44378500	45.12003100
H	38.95640800	74.04697300	43.51901300
H	40.43370500	74.46907000	46.06757300
N	42.00207600	74.10144600	43.52116800
C	43.31252100	73.82774600	42.88458000
C	44.12739100	75.13196700	42.94384100
O	45.00515800	75.29626300	43.83328700
C	43.13197500	73.17681300	41.49333800
C	44.39094800	72.47578400	40.92265900
C	43.99854200	71.67974900	39.66079000
C	45.54191400	73.45036200	40.60941300
H	41.41566100	74.82892000	43.12638100
H	43.81987700	73.12784500	43.55248700
H	42.34340700	72.42074800	41.61456900
H	42.75805300	73.90559300	40.76000400
H	44.74330500	71.75788300	41.68108000
H	46.40523900	72.92584600	40.18525200
H	45.90354800	73.96296900	41.50607000
H	45.21735700	74.21337300	39.89120600
H	43.22412800	70.93266900	39.87995500
H	43.60719800	72.35780100	38.89040900
H	44.86833500	71.15685800	39.24609000
N	43.76580800	76.11691000	42.07923700
C	44.44429800	77.41974500	42.03000900
C	44.57353100	78.09226000	43.38830600
O	45.56372800	78.82554400	43.65932600
H	43.15801000	75.91101200	41.28517100
H	45.44016400	77.36472300	41.57413500
H	43.84018800	78.06711200	41.38580600
N	43.57850300	77.89314400	44.28620000
C	43.58354300	78.49808700	45.62279600
C	44.89322000	78.26860900	46.39882700
O	45.23661600	79.11940000	47.26023500
C	42.39024600	77.95690100	46.42935700
H	42.80223700	77.30167000	44.02533400
H	43.50345900	79.58555800	45.52295900
H	42.47517000	76.87301500	46.56581200
H	42.37142000	78.42640200	47.41574700
H	41.43979000	78.16631700	45.92880500
N	45.57366400	77.11204900	46.16986600
C	46.84341000	76.82587900	46.83124600
C	48.06714100	76.72212300	45.90877800
O	49.14147400	76.27423000	46.37493400
H	45.25180400	76.45948800	45.45498900
H	47.01904500	77.62800300	47.55578600
N	47.90339300	77.13915700	44.62325400
C	48.93046000	76.95543700	43.59367200
C	49.46635900	78.26710500	43.00454600
O	50.61457500	78.28270700	42.47395600
C	48.36402500	76.06610000	42.44404100
C	49.43785400	75.54228600	41.46458100
C	48.85393400	74.87746200	40.22249300
O	49.07131000	73.66361300	39.95701100
N	48.10521800	75.67285400	39.40780400
H	46.99739700	77.49279200	44.34091700
H	49.77254600	76.45891600	44.08168200
H	47.84243000	75.22270900	42.91122200
H	47.62442800	76.65685700	41.89306800
H	50.06663400	74.79447500	41.95632300
H	50.08496100	76.37361200	41.16324000
H	47.82748200	76.65056800	39.61076100
H	47.72757800	75.23385700	38.57997300
N	48.63972900	79.34213300	42.97993200
C	49.00148500	80.55768500	42.22420900
C	49.89251700	81.48386100	43.07063900
C	47.74939700	81.28065600	41.69192700
C	46.73334700	80.38505100	40.94721600
C	47.36097000	79.43528800	39.91661900
O	48.06452400	79.95378200	38.97135300
O	47.12800500	78.17414800	40.07536300
H	47.70529500	79.27794500	43.36710300
H	49.58550300	80.21377700	41.36604100
H	47.23014300	81.78264700	42.52178900
H	48.11023900	82.06862000	41.02080700
H	46.17220700	79.79278800	41.66827500
H	46.01919700	81.03867500	40.42708000
C	47.31550400	83.09243500	38.39665500
C	48.15746400	84.21155900	38.97045900
O	47.64246900	85.32278700	39.29699900
C	46.82094100	83.38247700	36.95366900

C	45.74768700	82.33728800	36.56376300
C	47.99665900	83.40787900	35.95952400
C	45.03959700	82.62238700	35.22620700
H	47.82222900	82.11992600	38.43972300
H	46.34542700	84.37544800	36.95744500
H	46.21536600	81.34135400	36.54488900
H	44.99494800	82.30571600	37.36566600
H	48.73505400	84.16572800	36.25291500
H	47.66236700	83.64313100	34.94109800
H	48.50045300	82.43137200	35.93711500
H	44.24585900	81.88891100	35.03552200
H	44.57437200	83.61827500	35.23376800
H	45.73223100	82.58099000	34.37675900
N	49.49351300	83.96803400	39.13136400
C	50.37516100	84.96357100	39.74317300
C	51.39721800	85.61848800	38.80891500
O	51.54850600	86.86242000	38.79181200
H	49.87572600	83.04830500	38.92439100
H	49.74735200	85.76496200	40.13186700
N	52.16908900	84.76003000	38.06913900
C	53.31019500	85.24784300	37.30567800
C	53.37985800	84.76224000	35.86258100
O	54.46021500	84.80210100	35.23021500
H	52.06104100	83.74789100	38.21560700
H	53.25565400	86.34279700	37.31168600
N	52.20316900	84.33051700	35.31000800
C	52.13396600	83.76229500	33.96014700
C	51.49838000	84.72001600	32.93430000
O	51.23790500	84.32828900	31.76870800
C	51.41306500	82.40600600	33.96618200
H	51.40349000	84.23551200	35.92492700
H	53.17636700	83.62834400	33.64250000
H	51.34987700	82.01091800	32.95053100
H	51.94903500	81.68875500	34.59477700
H	50.39110800	82.50157400	34.35282600
N	51.27779400	85.98979600	33.35465500
C	50.72373200	87.03721400	32.50756500
C	49.64966200	87.82426200	33.26532900
O	50.33669400	88.28977600	34.47615600
C	48.40528600	87.00589200	33.60847200
H	51.52170700	86.25611300	34.29936100
H	50.31301100	86.56589600	31.61118300
H	49.36495200	88.69459100	32.65436200
H	49.69660700	88.59183900	35.15073400
H	47.68807300	87.60630100	34.18320900
H	47.90115300	86.66828400	32.69552800
H	48.67202500	86.12517300	34.20048000
H	42.11084500	87.67285200	42.51229600
C	42.68637600	87.58379600	43.43442000
S	42.03707300	86.15919000	44.47464200
H	42.62176900	88.49818300	44.02832700
H	43.73422500	87.38720800	43.18810000
C	52.58184200	81.12897500	38.51399100
O	51.69173700	82.02892200	38.54183300
N	52.40016200	79.82604800	38.75346600
C	51.11746900	79.16720100	39.07489600
C	50.89159600	77.92446100	38.19071000
C	50.70138800	78.23340600	36.71737100
C	49.53227800	78.89198100	36.28557300
C	51.66590800	77.87302100	35.76280500
C	49.34625800	79.17734900	34.92884000
C	51.47850500	78.16234500	34.40422500
C	50.31603800	78.81633100	33.98149300
H	50.30594900	79.88211300	38.92742700
H	51.72842100	77.22160600	38.32071600
H	49.99978400	77.42137000	38.58069300
H	48.79019500	79.17871100	37.02989300
H	52.56509500	77.35063900	36.08271300
H	48.43852100	79.68054100	34.60627100
H	52.23618800	77.87375800	33.68047900
H	50.16581900	79.04400900	32.92990300
H	53.21812300	79.23114900	38.70803400
C	40.15535200	84.97167300	40.43526800
C	44.85580200	85.38784200	41.56375600
C	44.06557500	82.96895700	45.69612000

C	39.31099800	82.92764700	44.75284700
C	41.50574200	85.26877900	40.36956500
C	42.15119900	85.93071600	39.24528000
C	43.48074700	86.06192100	39.56899800
C	43.64456300	85.46677400	40.89106500
C	44.57996400	86.70833100	38.78072300
C	41.44419000	86.38016300	38.00160000
H	40.71139500	87.17197600	38.21071300
C	45.04569900	84.80969900	42.80981800
C	46.33054700	84.69398500	43.47493300
C	46.11297200	83.97343700	44.63689700
C	44.68801000	83.66691600	44.67948000
C	47.62305400	85.23051300	42.94252200
C	47.06535200	83.52838900	45.64923300
C	48.22318800	84.12759100	45.98503600
C	42.70050400	82.74361600	45.80047200
C	42.06699700	82.10891300	46.94463100
C	40.70647800	82.12470700	46.69509000
C	40.53033300	82.74748700	45.38492300
C	42.80369800	81.54045900	48.11969700
C	39.60643900	81.60857600	47.50533300
C	39.54381300	81.56982200	48.84889900
C	39.12897700	83.47177300	43.49019000
C	37.83913200	83.58401600	42.82402500
C	38.08004300	84.14130600	41.59030400
C	39.51403700	84.37960900	41.51388000
C	36.53287200	83.15363900	43.42172100
C	37.10400100	84.47051500	40.50068200
H	37.12107400	85.53862100	40.24720300
N	42.43115900	85.00284300	41.36802200
N	44.04558900	84.21002400	43.57241000
N	41.75361500	83.13073300	44.86790000
N	40.13292600	83.96439700	42.67751800
Fe	42.11186600	83.93453700	43.04612400
H	40.90096100	85.55598800	37.52082000
H	42.15173800	86.77729100	37.26826700
H	46.80155200	82.61124300	46.17370300
H	38.75541300	81.21487200	46.94925000
H	36.07963600	84.22212900	40.79383900
H	37.32331600	83.91695200	39.57725000
H	48.55338200	85.05944200	45.53997900
H	48.87240300	83.69671700	46.74021300
H	40.32648100	81.97514500	49.48106900
H	38.68624900	81.13931800	49.35660300
H	35.70065900	83.33676300	42.73545000
H	36.31406800	83.69364600	44.35243000
H	36.52852900	82.08220300	43.66307400
H	44.30719800	86.79720800	37.72337600
H	44.79003700	87.72455400	39.14656000
H	45.52113700	86.14861500	38.84110900
H	42.16160400	80.85470500	48.68014600
H	43.69693600	80.98031900	47.81813500
H	43.12423300	82.32680700	48.81789900
H	48.45785700	84.59210400	43.24887100
H	47.83142200	86.24061600	43.32284200
H	47.62564800	85.28925500	41.85035000
O	41.97038500	76.18896500	39.79045800
C	42.47219400	77.09753200	38.85872100
C	43.26934600	76.63499100	37.80201100
O	43.47269800	75.25984900	37.74437100
C	44.40905600	74.75194000	36.75518200
C	43.81290300	77.55391600	36.89283500
C	43.56929900	78.92127400	37.05336600
C	42.78531900	79.39547800	38.10949500
C	42.24555700	78.47416800	39.01277900
O	41.46828100	78.79367200	40.12573200
C	41.38934700	80.19183000	40.55371600
H	44.44224500	77.21553700	36.08023700
H	44.01160500	79.62571300	36.35770600
H	42.62375900	80.45798500	38.23937000
H	41.28273500	76.58055300	40.39326600
H	45.39846000	75.20587500	36.87816500
H	44.46913800	73.68135700	36.94522200
H	44.04563800	74.93063200	35.73598200
H	40.84129100	80.17570800	41.49297100

H	42.38072100	80.61405900	40.72889400
H	40.84922500	80.79441200	39.81525700
O	42.36946600	82.48075800	42.31617900
H	54.26096900	84.96750300	37.77449800
H	50.92161500	84.49706700	40.57403600
H	51.51658300	87.73076600	32.20106300
H	35.59775100	82.29041900	35.38632400
H	50.20061900	82.35887600	42.48311800
H	49.35766300	81.83588300	43.96066200
H	50.79164400	80.94694200	43.38816900
H	45.73026600	85.76951500	41.05084000
H	44.68545600	82.59427900	46.50023100
H	38.42316000	82.60221200	45.28149100
H	39.54526900	85.24327000	39.58126300
H	46.43571100	83.01546400	39.04640700
H	38.18092600	74.19231700	40.08444400
H	37.02253900	76.51517700	43.95204700
H	39.76262000	72.89691200	45.60835800
H	53.62272500	81.39088600	38.27800800
H	51.12508300	78.87745000	40.13296100
H	46.79033400	75.87987800	47.38361000

$^{2}TS_{HA,A}$

C	38.66010500	78.93495700	34.97476800
C	38.77672400	77.45078200	35.38306100
C	38.95100100	79.92475800	36.11597300
C	40.20294000	77.01220000	35.75613800
H	39.34766700	79.12888000	34.13801100
H	38.41424100	76.82717400	34.55294100
H	38.09845800	77.25878200	36.22949000
H	38.81788500	80.96110800	35.78132000
H	38.27025900	79.75723100	36.96159700
H	39.97583100	79.82549400	36.48979000
H	40.23883000	75.94178700	35.99039200
H	40.58377700	77.55177000	36.62946100
H	40.89667000	77.19517100	34.92570800
H	50.91173500	90.63012700	30.09613100
C	50.65833000	89.70854500	29.55502100
C	49.30971500	89.16999500	29.98378500
C	49.13071300	88.63230900	31.27120200
C	48.20707400	89.20344500	29.11391800
C	47.88473300	88.14502800	31.67906800
C	46.95731700	88.71664800	29.51799800
C	46.79258100	88.18614700	30.80236500
H	51.46005200	88.98833400	29.75864200
H	50.67577200	89.93805600	28.48404700
H	49.95705900	88.58443600	31.97346800
H	48.32905500	89.61217800	28.11363500
H	47.79116600	87.73924200	32.68030500
H	46.11738500	88.75052400	28.82943700
H	45.82448200	87.80716700	31.11670900
C	38.18922200	76.24184200	41.32184300
C	38.73275200	76.88939000	42.58126500
O	39.95557900	77.17441600	42.72469300
C	36.94106400	76.92059400	40.69735000
C	37.20309900	78.40204100	40.36756700
C	36.50614600	76.13963300	39.44182600
H	39.00829600	76.22866900	40.59638900
H	36.10900500	76.86893500	41.42100600
H	36.29985100	75.08741400	39.67203800
H	37.29299700	76.17044500	38.67813400
H	35.59962500	76.57617300	39.00793900
H	37.42687900	78.98646600	41.26729100
H	38.05819300	78.50372100	39.69015900
H	36.32650100	78.85032700	39.88625800
N	37.85928900	77.11992100	43.60746000
C	38.26662000	77.86151600	44.80438200
C	39.13710900	77.09242100	45.80116900
O	39.67609200	77.70762500	46.74512100
H	36.87794800	76.93063100	43.46284500
H	38.84564600	78.74312800	44.51356800
N	39.27171000	75.74974600	45.59518300
C	40.12008500	74.91371300	46.43531800
C	41.31284500	74.26970200	45.72369600
O	41.85759400	73.26949300	46.24147500
H	38.76364800	75.32624500	44.83258200
H	40.49769300	75.55304400	47.24050900
N	41.73954000	74.85134700	44.56342800
C	42.91533600	74.31568900	43.84112900
C	43.88144300	75.47860800	43.56656000
O	44.84000200	75.70699600	44.36167500
C	42.50270500	73.46261500	42.61615900
C	43.54978400	72.40132400	42.19137000
C	42.98037900	71.54054000	41.04604600
C	44.90836700	73.00704200	41.79290900
H	41.23321500	75.63876900	44.17396200
H	43.42326300	73.67344800	44.56488300
H	41.58068900	72.93748700	42.89897100
H	42.24897400	74.09624800	41.75412900
H	43.71198700	71.74467100	43.06108400
H	45.61588000	72.22680800	41.49089300
H	45.37571500	73.54860100	42.62149000
H	44.79646700	73.70774500	40.95701500
H	42.03087000	71.06869800	41.33018400
H	42.80003600	72.15519900	40.15403100
H	43.68480700	70.74739300	40.77048900
N	43.62549800	76.27363700	42.50221000
C	44.53481800	77.35865200	42.11789400
C	44.72246600	78.42862500	43.18389700
O	45.77685000	79.11182300	43.23432400
H	42.89110600	76.05854200	41.82827700
H	45.51899400	76.97058600	41.83695700
H	44.12354900	77.83191100	41.22389300
N	43.70924900	78.61045600	44.06911200
C	43.78665000	79.61584400	45.13341400
C	45.02991500	79.42372400	46.02020400
O	45.52055000	80.41785600	46.61547700
C	42.49407900	79.57806800	45.96343500
H	42.84316500	78.10772900	43.93534300
H	43.90750600	80.60474000	44.68163500
H	42.31524000	78.58520500	46.38968100
H	42.55609700	80.29585400	46.78394000
H	41.63579300	79.85168400	45.34077800
N	45.50021600	78.15206300	46.15453300
C	46.75832800	77.85558600	46.83162800
C	47.93705500	77.43248800	45.93729600
O	49.10655900	77.59745200	46.35727700
H	45.03593600	77.40179200	45.65126200
H	47.07043200	78.75743300	47.36016500
N	47.65253300	76.82753700	44.74784900
C	48.71132600	76.29702600	43.88039400
C	49.39451300	77.35820300	43.00190800
O	50.55904000	77.12673800	42.56142900
C	48.10975000	75.19159100	42.96828000
C	49.17526900	74.33625900	42.24713000
C	48.59097100	73.43118300	41.16895200
O	48.62449800	72.17568300	41.26680700
N	48.04724200	74.05843200	40.08766700
H	46.69161600	76.61314500	44.49971100
H	49.50129800	75.88451300	44.51551400
H	47.48112500	74.54272100	43.59048800
H	47.46484400	75.67218600	42.22351200
H	49.68452000	73.68635600	42.96452000
H	49.92607100	75.00005700	41.80463100
H	47.89399200	75.07584600	39.99998800
H	47.67444800	73.46036800	39.36407800
N	48.67785100	78.44942400	42.64750000
C	49.22611700	79.41593700	41.67791300
C	50.11012600	80.45220400	42.39691900
C	48.12136300	80.07964800	40.83434000
C	47.10083500	79.12315200	40.17873100
C	47.68962800	77.87438700	39.51558900
O	48.57567500	78.03401100	38.59767800
O	47.22612200	76.73051100	39.91424500
H	47.74359300	78.60810600	43.02062400
H	49.86028600	78.83106100	41.00694900
H	47.56242500	80.79160700	41.45588800
H	48.62993700	80.65713800	40.05472600
H	46.38151200	78.81092200	40.93031100
H	46.54972700	79.68763200	39.41254300

C	48.36238200	80.29859300	36.33699500
C	48.69275700	81.54854200	37.13086600
O	47.96448900	82.57926500	37.17120500
C	47.11061600	80.39452300	35.44540000
C	46.74206300	78.97712700	34.94008100
C	47.30895000	81.39403600	34.28817900
C	45.40850000	78.88868300	34.18004800
H	49.23703400	80.03831600	35.72008300
H	46.28223300	80.76085300	36.07114100
H	47.55470500	78.61038500	34.29310400
H	46.71442100	78.29910000	35.80491000
H	47.56273500	82.38235000	34.68250000
H	46.40294200	81.49771000	33.68008300
H	48.12021100	81.05997000	33.62548900
H	45.18346300	77.85114800	33.90429100
H	44.57689900	79.25573500	34.79567100
H	45.42367800	79.47842500	33.25624500
N	49.86570700	81.46484200	37.83496300
C	50.35131300	82.55362100	38.66672300
C	51.43988900	83.39719000	38.01417600
O	51.49698300	84.64928500	38.21077100
H	50.39397000	80.59513800	37.83676600
H	49.52255100	83.22478800	38.89252400
N	52.33057000	82.75652600	37.22052600
C	53.44603200	83.46207300	36.59342300
C	53.07313000	84.47552700	35.50365200
O	53.95343800	85.24308500	35.05431100
H	52.32035000	81.73101800	37.20759700
H	54.01192700	84.03057400	37.33945400
N	51.77139400	84.49331400	35.09684600
C	51.23151300	85.51148400	34.19382200
C	50.44940100	86.61872800	34.93904600
O	49.77211600	87.44998500	34.27646400
C	50.36346800	84.87746600	33.09717400
H	51.14030500	83.79316800	35.46265500
H	52.10222900	86.00097400	33.74171000
H	49.93112500	85.66005200	32.47065400
H	50.96661700	84.20676200	32.47769900
H	49.53889800	84.30025800	33.53434200
N	50.56448900	86.65412600	36.28906000
C	49.90014000	87.66997300	37.11118300
C	48.87682600	87.10164500	38.11634600
O	49.52343000	86.43407600	39.24026800
C	47.81496500	86.20672600	37.47023800
H	51.13003500	85.95966800	36.76958800
H	49.40023700	88.35341500	36.41948200
H	48.38648700	87.96792400	38.57677700
H	50.15879000	85.74202700	38.93293900
H	47.04263100	85.96568100	38.20757100
H	47.34289200	86.71052400	36.61745900
H	48.24112400	85.26147300	37.11955400
H	40.73763600	86.62810000	40.34062200
C	41.69917500	86.98373100	40.72263500
S	42.03915300	86.17300800	42.38571800
H	41.65147700	88.06368100	40.87737300
H	42.48643800	86.72768300	40.01258400
C	53.14293800	79.11728500	38.18545100
O	52.26845100	79.93593100	37.77939600
N	52.91885800	77.97243400	38.84272900
C	51.60208700	77.39989700	39.19524600
C	51.44106800	75.96785300	38.64208300
C	51.45101000	75.88749100	37.12772800
C	50.35922400	76.38973800	36.39249600
C	52.53491500	75.32857000	36.43208600
C	50.36463700	76.33149700	34.99535700
C	52.53925400	75.27068800	35.03219600
C	51.45283100	75.77411600	34.30928000
H	50.81784000	78.04086100	38.79048100
H	52.22793700	75.32156900	39.05776700
H	50.48706700	75.59210600	39.02976800
H	49.52255700	76.82511000	36.93575200
H	53.37643200	74.92393400	36.99026600
H	49.51510300	76.71923100	34.43965200
H	53.38530000	74.83092800	34.51104200
H	51.45128200	75.72990000	33.22377100
H	53.73269000	77.43255200	39.10933500
C	40.05307700	84.06795100	39.35153200
C	44.86098700	83.80039100	39.95948800
C	44.26251400	83.37777400	44.74647400
C	39.46734900	83.80436000	44.16750700
C	41.41360000	83.94889600	39.09117000
C	42.00827600	83.96875700	37.76266700
C	43.37283000	83.90375900	37.93656100
C	43.60476000	83.83741200	39.37433600
C	44.45278600	83.93591200	36.89580200
C	41.23344200	84.07328700	36.48240200
H	40.72213000	85.04170400	36.39170200
C	45.12076000	83.71673000	41.32079100
C	46.45868300	83.66217400	41.89691400
C	46.28796800	83.47817400	43.25502000
C	44.84398200	83.46868400	43.49050700
C	47.72775400	83.75384000	41.11026700
C	47.28212000	83.26479600	44.30567500
C	48.49892200	83.83558800	44.37168400
C	42.90436400	83.46189400	45.01756400
C	42.32698300	83.36893500	46.34746300
C	40.95839900	83.50172000	46.18899300
C	40.71964300	83.65457100	44.75246000
C	43.11284300	83.11397700	47.59821200
C	39.90067000	83.46480300	47.19281300
C	39.99571300	83.83531500	48.48378200
C	39.21010800	83.94585500	42.81476900
C	37.88615900	84.13027500	42.24001800
C	38.04810100	84.21217200	40.87576900
C	39.47204400	84.07675800	40.61284400
C	36.62097700	84.22328900	43.03985900
C	37.00589600	84.41047900	39.81572600
H	37.15023200	85.35575000	39.27533000
N	42.39894000	83.85206800	40.06143000
N	44.15757000	83.62289000	42.30656100
N	41.91471800	83.64405300	44.06618200
N	40.16987800	83.92680800	41.80468300
Fe	42.16303700	83.65498800	42.05189100
H	40.46453300	83.29333600	36.40319200
H	41.89100100	83.97260700	35.61431100
H	46.99667100	82.55903200	45.08637800
H	38.93582400	83.09309400	46.84717300
H	35.99895600	84.43071300	40.24309000
H	37.02803800	83.60722000	39.06711300
H	48.83976000	84.56931700	43.64796500
H	49.18654100	83.58985700	45.17474800
H	40.90271600	84.25396400	48.90571800
H	39.14782700	83.74365800	49.15554900
H	35.74431600	84.32504000	42.39316600
H	36.63245700	85.08942000	43.71479400
H	36.46656800	83.33290800	43.66411000
H	44.05422900	83.70453800	35.90295300
H	44.92048900	84.92801100	36.83260900
H	45.26241100	83.22652200	37.10091600
H	42.47764900	82.66410800	48.36817300
H	43.95178800	82.43267200	47.41495400
H	43.52574200	84.04109100	48.02095600
H	48.55417800	83.29790000	41.66326000
H	48.01549000	84.79070100	40.88514900
H	47.63782000	83.23456500	40.15020500
O	41.53905100	76.78405600	40.58785100
C	42.09106500	77.64112600	39.64285200
C	43.11890500	77.15613300	38.81189800
O	43.47026700	75.83208600	39.00163000
C	44.67869600	75.33713400	38.33216900
C	43.73301800	78.02380300	37.89828300
C	43.32998200	79.36243900	37.82381900
C	42.31419000	79.85856100	38.64541400
C	41.69917800	78.98358400	39.54720200
O	40.68384600	79.34094200	40.44455800
C	40.31836900	80.69252000	40.62818100
H	44.54463100	77.67230700	37.27604000
H	43.83349900	80.03318000	37.13682000
H	42.05124500	80.90726500	38.61337100
H	40.95020000	77.23664900	41.24953000

H	45.56266300	75.88557600	38.67757900
H	44.74806800	74.29288700	38.63439000
H	44.56818600	75.39760000	37.24339200
H	39.45776300	80.72808900	41.28996800
H	41.33520900	81.38536400	41.39139900
H	40.23246100	81.27523600	39.71119300
O	42.07304600	81.87137500	42.07450700
H	54.11803400	82.71831600	36.15645600
H	50.73794400	82.15434600	39.61191400
H	50.65650400	88.23159800	37.67275900
H	37.64660300	79.11913100	34.58929100
H	50.57559300	81.13580300	41.67405000
H	49.52039100	81.04849200	43.10215900
H	50.90639700	79.94313500	42.94844500
H	45.71513300	83.82459400	39.29248000
H	44.92710500	83.23097600	45.58799100
H	38.61198300	83.83517500	44.83253500
H	39.39171100	84.17023900	38.49835500
H	48.26556100	79.47611600	37.06669300
H	37.95204900	75.19176700	41.55071700
H	37.36848400	78.20406900	45.32576100
H	39.54751500	74.10170700	46.89298500
H	54.20839500	79.32211900	38.01313100
H	51.49939200	77.38569200	40.28720600
H	46.60525600	77.06250600	47.57529900

$^{2}IM1_{HA,A}$

C	37.19612000	81.79568300	36.29722000
C	38.02390500	80.98716500	35.27567800
C	37.21511500	81.21685000	37.72238000
C	39.53878500	81.00011200	35.54050000
H	37.56727300	82.83153900	36.31776900
H	37.83238400	81.39015200	34.27079300
H	37.66139000	79.94771600	35.26334000
H	36.56854500	81.79934400	38.39093900
H	36.85159800	80.18063100	37.72677400
H	38.22274500	81.21576600	38.15301200
H	40.08036100	80.45803600	34.75663500
H	39.79419800	80.53124800	36.49699000
H	39.92316400	82.02817000	35.55958700
H	51.44402000	90.31785800	30.07010200
C	51.82888600	89.58150300	29.35365800
C	50.78641200	88.53372300	29.02453500
C	50.14955700	87.81244600	30.05064100
C	50.43792000	88.25213200	27.69302800
C	49.19313500	86.83602900	29.75456200
C	49.48047800	87.27502000	27.39171500
C	48.85539100	86.56389800	28.42216700
H	52.72032600	89.12978700	29.80860500
H	52.15113900	90.12144300	28.45654200
H	50.38746300	87.99870900	31.09316400
H	50.92000000	88.80176100	26.88790500
H	48.72758500	86.30476200	30.57729000
H	49.22509700	87.07206800	26.35508800
H	48.11295100	85.80571000	28.19018600
C	37.47216500	75.72643300	40.74519200
C	38.11153100	76.65211900	41.75544400
O	39.35838000	76.82423000	41.84490800
C	36.93211700	76.45272200	39.47409100
C	38.06378000	77.12885400	38.67917900
C	36.16125500	75.44446100	38.60048200
H	38.22992500	74.99568100	40.44565700
H	36.22470600	77.23045900	39.80453400
H	35.33208800	74.98108100	39.14943300
H	36.82721000	74.64479200	38.25257200
H	35.74689400	75.94104600	37.71619100
H	38.58050100	77.90163800	39.25661900
H	38.81125400	76.38920000	38.36575300
H	37.66216900	77.60183000	37.77576900
N	37.27541300	77.31768300	42.60998100
C	37.76517700	78.38751300	43.48516300
C	38.55445300	77.94455100	44.71926900
O	39.06782200	78.81596800	45.45735900
H	36.27814600	77.18604400	42.51824800
H	38.43666700	79.04361000	42.92099300
N	38.66975400	76.60321200	44.93259600
C	39.50359600	76.03562200	45.98443500
C	40.70085800	75.20781700	45.49815700
O	41.26602000	74.43980200	46.30697400
H	38.17326500	75.97765800	44.31547900
H	39.87833600	76.87176800	46.58353400
N	41.10076200	75.38382500	44.20190700
C	42.25435100	74.64375600	43.63702100
C	43.41042400	75.63972800	43.45269200
O	44.42089600	75.60497400	44.21321700
C	41.83624600	73.85350400	42.37476800
C	42.83647800	72.76292900	41.91254500
C	42.18166600	71.90462300	40.81045300
C	44.18004000	73.33506600	41.41990200
H	40.57441700	75.99497500	43.58427800
H	42.57881800	73.95607300	44.42132900
H	40.88334700	73.36583500	42.62416300
H	41.63188200	74.53631600	41.53800700
H	43.03594400	72.10872400	42.77647900
H	44.83616600	72.54358800	41.04264900
H	44.73323500	73.82933700	42.22370000
H	44.01617100	74.06335500	40.61518100
H	41.24082000	71.45465400	41.15376200
H	41.96456800	72.51522700	39.92398000
H	42.85305400	71.09522100	40.50246700
N	43.24081900	76.61160500	42.52206700
C	44.23568800	77.66629100	42.33463800
C	44.48344800	78.53770000	43.56339800
O	45.53313000	79.22808400	43.63709000
H	42.53875500	76.52459600	41.77931700
H	45.20689100	77.24936800	42.05159100
H	43.90205900	78.30186700	41.51028800
N	43.55849900	78.51307200	44.55388300
C	43.76894200	79.19226100	45.83843200
C	45.07198100	78.74311400	46.52902700
O	45.68802400	79.54517600	47.26997000
C	42.56204000	78.93055300	46.75524100
H	42.70746700	77.98685500	44.41405500
H	43.88470800	80.26608700	45.66949000
H	42.48065100	77.86175200	46.99027600
H	42.68982600	79.47766700	47.69223800
H	41.63069100	79.26336000	46.28401500
N	45.43780600	77.44104800	46.35239600
C	46.67704200	76.91086700	46.91114100
C	47.80478100	76.63086900	45.90758000
O	48.99394200	76.63945700	46.29855000
H	44.88169200	76.83893600	45.75158300
H	47.06113300	77.64255500	47.62319200
N	47.44292800	76.31737900	44.62652100
C	48.44035600	75.93765900	43.61806900
C	49.05064500	77.13175000	42.86095600
O	50.17974600	76.99298000	42.31833000
C	47.77997500	74.94960400	42.61981600
C	48.71057700	74.43748600	41.50059100
C	47.94443000	73.54931300	40.53024300
O	47.51525100	72.41214000	40.86170900
N	47.73495100	74.08498000	39.29755200
H	46.46118100	76.19960000	44.39446100
H	49.27752400	75.45644300	44.13157400
H	47.39551700	74.09290000	43.18506500
H	46.91199700	75.44596100	42.16165400
H	49.51652200	73.84010000	41.94177900
H	49.17591800	75.27222600	40.97419700
H	47.97698900	75.05226600	39.05369900
H	47.23957700	73.51945800	38.62371200
N	48.28133700	78.23992600	42.72305500
C	48.66625200	79.37269700	41.86137600
C	49.00224700	80.60691500	42.71541300
C	47.54739400	79.67340400	40.83910400
C	47.15766200	78.48710700	39.94617000
C	48.22369200	78.01830100	38.94283300
O	49.07390300	78.88218000	38.50863400
O	48.17474600	76.78893300	38.56067900
H	47.42193700	78.34248800	43.25649500
H	49.56022400	79.04693700	41.32296600

H	46.66176700	80.02316700	41.38519200
H	47.88853600	80.49828100	40.20310000
H	46.87750600	77.62015500	40.55284600
H	46.26488100	78.75951100	39.36240000
C	50.08139900	79.92419800	35.25459500
C	49.66792200	81.25468900	35.85398600
O	49.69931200	82.33590200	35.18687400
C	48.90712900	78.91861100	35.09100000
C	49.44787400	77.52576800	34.68511100
C	47.85924500	79.45099300	34.09505400
C	48.43115600	76.39071000	34.89617900
H	50.54057800	80.13409700	34.28289800
H	48.44241600	78.80480600	36.07808300
H	49.78039200	77.55527400	33.63513400
H	50.33594500	77.30325400	35.29235300
H	47.47575000	80.43123300	34.40089000
H	47.00557400	78.76865200	34.01164200
H	48.29825300	79.56648700	33.09483100
H	48.85454700	75.42493600	34.59236800
H	48.16514900	76.32889800	35.95839800
H	47.51571000	76.54625000	34.31125700
N	49.26903900	81.23168900	37.15090000
C	48.75742300	82.43025000	37.79854400
C	49.72445500	83.60716400	37.81380800
O	49.25845100	84.79574600	37.81446000
H	49.21769600	80.33989500	37.67630200
H	47.84676600	82.79803700	37.31263000
N	51.04046500	83.35409700	37.82671300
C	52.04549600	84.41130200	37.76149800
C	52.06607800	85.23227300	36.46506600
O	52.74643200	86.28649000	36.42737600
H	51.39675700	82.39586200	37.84063900
H	51.90324900	85.13326400	38.57126400
N	51.32408600	84.76969500	35.42173100
C	51.15259900	85.53274300	34.18489800
C	49.76785700	86.21391300	34.11362000
O	49.35210100	86.70350000	33.02822200
C	51.38285100	84.64105700	32.95454200
H	50.83173300	83.87563900	35.47642600
H	51.89678000	86.33796500	34.21232700
H	51.14606700	85.19391200	32.04284900
H	52.42736500	84.31523000	32.92112700
H	50.74457800	83.75201400	33.00618900
N	49.06284000	86.27539400	35.27189800
C	47.71998100	86.84698900	35.34863700
C	46.62166300	85.80415900	35.64616300
O	46.65592000	85.35844700	37.04092600
C	46.64922400	84.60784700	34.69045800
H	49.44651000	85.85407100	36.11356100
H	47.52475900	87.32423100	34.38403100
H	45.65864000	86.32182200	35.55592300
H	47.57902100	85.11961500	37.32328100
H	45.80591000	83.94318800	34.90566500
H	46.57987000	84.93942700	33.64741500
H	47.57590400	84.03528400	34.80260400
H	39.98206800	86.50279500	42.12583100
C	40.88540500	87.05484200	42.40164400
S	41.84841000	86.07038400	43.68359000
H	40.60712000	88.01020600	42.85129400
H	41.49699300	87.20902900	41.51130000
C	53.47838800	80.21298600	37.53084000
O	52.86554800	81.27628700	37.80772500
N	53.25128800	79.01507200	38.09525200
C	52.22538400	78.76231600	39.12924100
C	52.53424900	77.45558500	39.88635500
C	52.64000800	76.24995100	38.96665600
C	51.50626800	75.75412800	38.29052700
C	53.87809700	75.61630200	38.75465300
C	51.61832600	74.65117000	37.43764700
C	53.98931300	74.51365300	37.89615600
C	52.85739200	74.02745700	37.23516900
H	51.23052800	78.70957400	38.67220800
H	53.47292800	77.57099300	40.44445500
H	51.73911600	77.30474700	40.62737300
H	50.53444300	76.22221600	38.42855500

H	54.75664600	75.97683400	39.28531700
H	50.73025600	74.27466000	36.93932100
H	54.95417500	74.03498300	37.75305200
H	52.93630200	73.16914900	36.57426700
H	53.76320000	78.20970700	37.75515200
C	39.69542300	84.15475800	40.63598700
C	44.46019300	84.98814200	40.23756500
C	45.09855600	83.70778200	44.87590100
C	40.35476400	82.76571700	45.23428600
C	40.94178700	84.49936800	40.12310400
C	41.17661600	84.93731300	38.75403900
C	42.52560800	85.17880000	38.64130900
C	43.10961900	84.88291400	39.94600400
C	43.29377300	85.67246200	37.45299400
C	40.11105200	85.09252900	37.71039600
H	39.39747400	85.88766400	37.96773700
C	45.05670700	84.69905600	41.46154600
C	46.47629400	84.85049300	41.73328100
C	46.66641900	84.47387200	43.05250000
C	45.35155400	84.10224000	43.56798200
C	47.50427000	85.29691300	40.74198400
C	47.89900500	84.38752200	43.82687500
C	49.04297000	85.06614100	43.61498800
C	43.85895000	83.36789600	45.39556900
C	43.62568900	82.95906900	46.77058800
C	42.27091500	82.69757500	46.87295500
C	41.69758300	82.93256200	45.54889700
C	44.70585500	82.77727800	47.79427500
C	41.48883300	82.24737900	48.02441100
C	41.67327200	82.60021600	49.30933400
C	39.76241400	83.05716400	44.01969300
C	38.34570600	82.87908900	43.73167900
C	38.14651500	83.30110500	42.43799200
C	39.44715000	83.71996800	41.92992700
C	37.35602700	82.32138100	44.71085300
C	36.87184200	83.34639800	41.64925200
H	36.60622900	84.37376500	41.36552400
N	42.12497900	84.48064200	40.83448300
N	44.38962100	84.24136800	42.58612600
N	42.67406700	83.35776000	44.67130300
N	40.41542000	83.57550800	42.90939100
Fe	42.41909500	83.84752600	42.72168300
H	39.53152100	84.16925600	37.57748100
H	40.54562800	85.34949600	36.73998000
H	47.88248600	83.68373000	44.65779200
H	40.67256700	81.56039500	47.79996600
H	36.03266000	82.94221400	42.22390200
H	36.94574300	82.76685500	40.71906300
H	49.15269300	85.80884400	42.83330900
H	49.91064500	84.89864800	44.24493800
H	42.43364600	83.31142100	49.61436600
H	41.04685500	82.19366600	50.09736300
H	36.36804800	82.19576100	44.25558500
H	37.22980900	82.97999900	45.58078900
H	37.68418800	81.34485000	45.09201100
H	42.74192100	85.49566800	36.52382300
H	43.48228100	86.75358600	37.51884000
H	44.27398400	85.19223500	37.36419000
H	44.29790100	82.38429600	48.72733900
H	45.45296900	82.05138800	47.45172200
H	45.21675300	83.72189300	48.02331500
H	48.44538800	84.75497900	40.88515100
H	47.73721900	86.36600500	40.84666500
H	47.18754900	85.14332300	39.70867100
O	41.51898400	76.81239800	40.26482500
C	42.06986000	77.77073500	39.41761900
C	43.18799800	77.42559400	38.63438300
O	43.62842800	76.11974100	38.75131300
C	44.78659100	75.69859000	37.96377500
C	43.78495000	78.39896400	37.82060100
C	43.28118700	79.70682400	37.80148300
C	42.17672300	80.06172600	38.57858700
C	41.58466600	79.08237500	39.38170800
O	40.44429200	79.31730700	40.16961700
C	40.28133100	80.57117600	40.77921200

H	44.64684000	78.14719400	37.21652900
H	43.75983700	80.45265200	37.17695800
H	41.78628500	81.07161100	38.58354400
H	40.64726100	77.07377800	40.65619500
H	45.69012700	76.25440100	38.23402600
H	44.92048800	74.64915300	38.21954500
H	44.58170300	75.80214200	36.89194900
H	39.30584100	80.66867300	41.23500600
H	43.41744700	81.72002200	42.56207300
H	41.16504000	81.12118000	41.10835300
O	42.61435800	82.11174400	42.15703300
H	53.01886500	83.93254200	37.89019300
H	48.50075900	82.16742600	38.82999500
H	47.69138000	87.61398200	36.13192800
H	36.15505900	81.85000500	35.94679700
H	49.31058700	81.44674300	42.08126600
H	48.12783400	80.92153500	43.29759400
H	49.81701900	80.38071400	43.41092300
H	45.10902800	85.32091800	39.43462500
H	45.94027700	83.66028700	45.55536800
H	39.70703300	82.39448900	46.01876800
H	38.84984000	84.21550100	39.96002700
H	50.83828900	79.46845700	35.90468300
H	36.64679200	75.17917500	41.22157900
H	36.91337400	78.98495600	43.81924800
H	38.92049800	75.38851500	46.64631800
H	54.28327700	80.20715500	36.78081800
H	52.22745500	79.61321000	39.81783100
H	46.47179300	75.97921700	47.45444500

$^{2}P_{hy,A}$

C	37.63706600	82.45925900	35.51937400
C	38.81113100	81.45750800	35.54983100
C	36.71788700	82.39249500	36.75157900
C	39.82667600	81.71127400	36.67680400
H	38.03912000	83.47842700	35.42033900
H	39.33308900	81.49718100	34.58372100
H	38.40916500	80.43654800	35.63911200
H	35.87182400	83.08196000	36.65034900
H	36.31091600	81.38075100	36.88189000
H	37.25356700	82.65783600	37.67093700
H	40.65908100	81.00031900	36.63011600
H	39.36436700	81.61374400	37.66676200
H	40.24924300	82.72156800	36.60718800
H	50.89546100	89.69764500	29.38222500
C	51.31266700	88.89247300	28.76449800
C	50.29779200	87.79334600	28.53000000
C	49.67343900	87.15378900	29.61622000
C	49.96156500	87.38333700	27.22903800
C	48.74144200	86.13195000	29.40905000
C	49.02834100	86.36050200	27.01641200
C	48.41579400	85.73182800	28.10624600
H	52.19838100	88.51325800	29.29089400
H	51.65019100	89.33383300	27.82036900
H	49.90309300	87.44076800	30.63758200
H	50.43437300	87.86823400	26.37814300
H	48.28569100	85.66729500	30.27626100
H	48.78237000	86.05753300	26.00215200
H	47.69255400	84.93802500	27.94278900
C	38.42609400	77.18331100	40.69551000
C	38.88345800	77.66741700	42.05696400
O	40.10591800	77.77149900	42.36002300
C	37.28447900	77.99241600	40.02730500
C	37.66303000	79.47616600	39.85761400
C	36.92530300	77.35096600	38.67262400
H	39.31176000	77.18866800	40.05234900
H	36.38511500	77.93314800	40.66512400
H	36.63134800	76.30104700	38.78936000
H	37.78272300	77.38770800	37.98952400
H	36.09506800	77.88563100	38.19837300
H	37.83260900	79.96943800	40.82189800
H	38.58097200	79.57368400	39.26675100
H	36.86593900	80.02183500	39.34079300
N	37.93356600	77.94392700	42.99899200
C	38.29589400	78.55387600	44.28229400
C	39.05127400	77.64593900	45.25389200
O	39.64622400	78.15323100	46.23018200
H	36.95964000	77.91352400	42.73355900
H	38.93516600	79.42741400	44.12096700
N	39.00670200	76.30523200	45.00655100
C	39.68743200	75.32508100	45.84492300
C	40.80033300	74.52635200	45.15751300
O	41.15845700	73.43753400	45.65633600
H	38.46590600	75.98259000	44.21708900
H	40.12121200	75.87386000	46.68761000
N	41.37049600	75.08187400	44.04507000
C	42.48709400	74.41632900	43.33166500
C	43.70827100	75.34294100	43.42704800
O	44.68032200	75.06636200	44.18693400
C	42.07602300	73.99531200	41.90013000
C	42.97290200	72.90848400	41.25383900
C	42.38509300	72.51239200	39.88424600
C	44.44379200	73.34307300	41.10595000
H	41.00507700	75.95226600	43.67189300
H	42.72725900	73.53172500	43.92562500
H	41.05391100	73.59899900	41.97527100
H	42.02836700	74.86825300	41.23362800
H	42.94040800	72.02297600	41.90877900
H	45.03548400	72.58296800	40.58474700
H	44.92525100	73.49584800	42.07655900
H	44.51039800	74.27961300	40.53798200
H	41.34574900	72.17000600	39.97365000
H	42.40391000	73.36889300	39.19739600
H	42.97082000	71.70424600	39.43181000
N	43.63432800	76.51435100	42.74588500
C	44.69577700	77.51516300	42.82824800
C	44.96948100	78.06202200	44.22786300
O	46.05962000	78.64466200	44.46132200
H	42.97241900	76.64196100	41.97403000
H	45.64619700	77.11411100	42.46455500
H	44.42470000	78.34422700	42.16870000
N	44.02579800	77.87609500	45.18439000
C	44.26765100	78.21780800	46.59251500
C	45.51025700	77.51278400	47.17336200
O	46.12213200	78.05119900	48.12333600
C	43.02259300	77.88084500	47.42966700
H	43.14996500	77.43968000	44.93302300
H	44.48756700	79.28569200	46.66891300
H	42.82098400	76.80261600	47.40608200
H	43.19936500	78.16946500	48.46894900
H	42.13852900	78.41375700	47.06600700
N	45.82674100	76.28907100	46.65707200
C	47.03074200	75.57820900	47.07299800
C	48.15521300	75.48123600	46.03181400
O	49.33170300	75.29257800	46.41516600
H	45.25495400	75.88618800	45.91990500
H	47.44177500	76.10133800	47.93793700
N	47.79869700	75.54027200	44.71150600
C	48.77387600	75.32275400	43.63511500
C	49.36892200	76.61484700	43.04464100
O	50.45302600	76.54198200	42.40400400
C	48.08895300	74.48934400	42.51584300
C	49.00213700	74.10213500	41.33283800
C	48.19853000	73.40548900	40.24414100
O	47.75653600	72.23432100	40.38532200
N	47.96218400	74.14908700	39.12913600
H	46.81449400	75.55275800	44.46369800
H	49.61603300	74.76951500	44.05950600
H	47.68542700	73.57682000	42.96936900
H	47.23091700	75.06220700	42.13439300
H	49.77212800	73.40537200	41.68213100
H	49.51296400	74.98244600	40.93977900
H	48.20592400	75.14268000	39.04280400
H	47.43514200	73.71318800	38.38634500
N	48.63532400	77.75102400	43.14790100
C	48.98751000	78.99083400	42.42981800
C	49.33633900	80.10726900	43.42796300
C	47.84175200	79.41494700	41.48140400
C	47.41764000	78.34437400	40.46545900
C	48.39993800	78.08893200	39.31190500

O	49.16014400	79.05982700	38.94642200
O	48.36459800	76.93104000	38.74559300
H	47.83100600	77.79604100	43.76900300
H	49.86836500	78.74660500	41.83023300
H	46.97828200	79.70683200	42.09417100
H	48.17332300	80.30380100	40.93260600
H	47.22988200	77.38480500	40.95748600
H	46.46783100	78.64947500	40.00001100
C	50.14403200	79.80318700	35.40867700
C	49.58632400	81.11517500	35.92624400
O	49.51152600	82.15067200	35.19240900
C	49.08540500	78.66839400	35.31528400
C	49.76793900	77.33988700	34.90888900
C	47.94476600	79.05438200	34.35407500
C	48.88087600	76.10414500	35.13553400
H	50.57492300	80.00205600	34.42179800
H	48.67414800	78.51927600	36.32215400
H	50.08340200	77.39865200	33.85504000
H	50.68199500	77.21935400	35.50636400
H	47.46199700	79.99058500	34.65723600
H	47.17383100	78.27628100	34.31835400
H	48.32841900	79.19749500	33.33497400
H	49.41778100	75.18329300	34.87584500
H	48.59188000	76.04970400	36.19174900
H	47.96934000	76.13494500	34.52554500
N	49.17009300	81.12382700	37.21521700
C	48.51018100	82.28583200	37.79233800
C	49.31762200	83.57558000	37.71861600
O	48.71113300	84.69094900	37.59489000
H	49.22922300	80.27570100	37.80795900
H	47.55744200	82.50029800	37.29557100
N	50.65420500	83.49273700	37.79602000
C	51.52188400	84.66112800	37.68208800
C	51.51464600	85.36905800	36.32052300
O	52.07703400	86.48651900	36.21700500
H	51.12538000	82.59340600	37.92023600
H	51.24563800	85.42295400	38.41749300
N	50.87784000	84.74090100	35.29482500
C	50.68588600	85.37246600	33.98929600
C	49.24334700	85.89058600	33.80237300
O	48.83981500	86.24976100	32.66273000
C	51.06614700	84.40880500	32.85348400
H	50.47923000	83.80698400	35.41047500
H	51.33925700	86.25314100	33.97196500
H	50.81999700	84.85461400	31.88729900
H	52.13869700	84.19304400	32.89173400
H	50.51865300	83.46577600	32.95877200
N	48.47217000	85.96005500	34.91732900
C	47.07599800	86.39173400	34.88192200
C	46.07695200	85.28733100	35.28631300
O	46.09314300	85.03876200	36.72798900
C	46.26120000	83.98528900	34.50167300
H	48.85117300	85.64910000	35.80772200
H	46.87518200	86.71779900	33.85735700
H	45.07173400	85.68548000	35.10069300
H	47.01507800	84.85978800	37.05234100
H	45.48811700	83.26678100	34.79449700
H	46.18270200	84.16485600	33.42246000
H	47.24202500	83.53972800	34.69859900
H	39.41137200	86.52931500	42.89035400
C	40.36186400	87.03980600	42.72843500
S	41.78462200	86.04129800	43.46641800
H	40.36082000	87.99978600	43.25118500
H	40.52168600	87.20127200	41.66138800
C	53.35692200	80.58846900	37.80839000
O	52.63749100	81.57334600	38.11575600
N	53.29143700	79.37323600	38.37875700
C	52.33344400	79.00689900	39.44405400
C	52.75679400	77.68801400	40.12065000
C	52.91321000	76.53645100	39.14111700
C	51.79152300	75.99007600	38.48526600
C	54.18259600	76.00047500	38.85848700
C	51.94429500	74.93005300	37.58558400
C	54.33557900	74.94277200	37.95113200
C	53.21474200	74.40327600	37.31323300

H	51.32334700	78.91959900	39.02824400
H	53.70296300	77.84048800	40.65681100
H	51.99782300	77.43556500	40.87229200
H	50.79771000	76.38697500	38.67592000
H	55.05416700	76.39920000	39.37312900
H	51.06489900	74.50967100	37.10771900
H	55.32463900	74.53886200	37.75358800
H	53.32622100	73.57760600	36.61658000
H	53.87647900	78.63096100	38.01394200
C	39.15831200	84.63616700	40.36830700
C	43.97886200	85.14583300	40.03284300
C	44.53551800	83.28760600	44.48025700
C	39.73319000	82.61991600	44.75740700
C	40.42425000	84.94973900	39.89314000
C	40.69821300	85.56446100	38.60173000
C	42.06373200	85.69918400	38.50637900
C	42.62353600	85.17397000	39.74674700
C	42.87613900	86.28959900	37.39318300
C	39.64990500	85.98259400	37.61385600
H	39.02657400	86.79913700	38.00367500
C	44.54838100	84.68456200	41.21062200
C	45.97794700	84.70067300	41.48447400
C	46.14225600	84.16975000	42.74990900
C	44.80438000	83.82779500	43.23231400
C	47.03103100	85.16925700	40.53090900
C	47.36829500	83.90970100	43.49644700
C	48.53849500	84.56653900	43.38489300
C	43.27907500	82.93745400	44.94853800
C	43.01324700	82.37097600	46.25733500
C	41.65070900	82.13765900	46.31892400
C	41.08952500	82.59521500	45.05171000
C	44.05738900	82.08088500	47.29208600
C	40.87073800	81.49844300	47.37877300
C	41.06426600	81.64072600	48.70248700
C	39.15886400	83.13800500	43.60323500
C	37.72716400	83.15941900	43.33581100
C	37.56067200	83.72920000	42.09308900
C	38.88964500	84.05722300	41.60109700
C	36.67538800	82.67243800	44.28824600
C	36.28733500	84.00329900	41.34873100
H	36.12377400	85.08019100	41.20840100
N	41.61020100	84.71957100	40.57866000
N	43.85231700	84.14757400	42.28240100
N	42.09895600	83.05935700	44.22013300
N	39.85101100	83.69324900	42.53545000
Fe	41.84902600	84.00395000	42.43796300
H	38.97399300	85.15717300	37.35438400
H	40.10484100	86.33775400	36.68457200
H	47.32051300	83.08704400	44.20883800
H	40.08470300	80.82020600	47.04531200
H	35.41710100	83.61053200	41.88362700
H	36.29200400	83.54824000	40.34927700
H	48.67331700	85.41938200	42.72910700
H	49.39975400	84.27096800	43.97539300
H	41.80997100	82.31431600	49.11251800
H	40.46143600	81.09150600	49.41906100
H	35.68043200	82.69559000	43.83289100
H	36.63386100	83.28955400	45.19568200
H	36.86412100	81.64133200	44.61680100
H	42.30994200	86.31071100	36.45614800
H	43.17006300	87.32458400	37.61817900
H	43.80124700	85.72819000	37.21899100
H	43.70760800	81.32427900	48.00071300
H	44.98759700	81.71495900	46.84356800
H	44.31196200	82.97664700	47.87547200
H	47.95966300	84.60617500	40.67095600
H	47.27783900	86.22998100	40.67990500
H	46.72994300	85.05622300	39.48705000
O	42.06093900	77.43343800	40.56101700
C	42.69803100	78.31512800	39.68900500
C	43.71230600	77.84037800	38.83812100
O	43.99549000	76.48858000	38.91710300
C	45.05945600	75.95639500	38.06273600
C	44.37058700	78.74173900	37.98785400
C	44.02968700	80.09908100	38.00074500

C	43.02458500	80.58264600	38.84387700
C	42.36284300	79.67346800	39.67207100
O	41.30439100	79.98924900	40.55169900
C	41.00233100	81.35593800	40.85865900
H	45.16247600	78.39538700	37.33735100
H	44.56373700	80.78998900	37.35822000
H	42.80163200	81.64100300	38.87827600
H	41.33710600	77.85468700	41.09931400
H	46.02653300	76.41778200	38.28908300
H	45.09708300	74.89571400	38.30324800
H	44.80535900	76.09355900	37.00531000
H	40.05795500	81.31508100	41.39768600
H	42.17995700	81.50032100	42.51174600
H	40.92608900	81.97668500	39.96773400
O	42.03896100	81.96338200	41.65680000
H	52.53910700	84.32588600	37.89806600
H	48.29814600	82.05060900	38.84063500
H	46.93583500	87.24682900	35.55452500
H	37.03715700	82.27595600	34.61668800
H	49.58788200	81.03379300	42.89819800
H	48.48448900	80.30918500	44.08885600
H	50.19027900	79.81649000	44.04865700
H	44.64988100	85.51600200	39.26710500
H	45.37308600	83.13201800	45.14781900
H	39.06088400	82.24151400	45.51901500
H	38.31349600	84.87212900	39.73120700
H	50.94920500	79.47119200	36.07668400
H	38.11771600	76.13122900	40.79411800
H	37.37836400	78.89416500	44.77169000
H	38.98067400	74.59536400	46.25057400
H	54.12559400	80.67215700	37.02529900
H	52.31392100	79.82235400	40.17466000
H	46.77739900	74.55601000	47.38373000

$^{4}Re_{B}$

C	36.21195200	79.46342300	38.82859100
C	36.08752100	78.25112200	37.88093800
C	37.54190300	79.52392200	39.59956000
C	37.04936200	78.29703900	36.68193400
H	36.08441500	80.38635400	38.24188800
H	35.05422300	78.19079900	37.50943100
H	36.26184300	77.32927400	38.45643700
H	37.55424200	80.36648100	40.30096800
H	37.68925900	78.60464400	40.18341400
H	38.40710200	79.64758800	38.94031400
H	36.92552400	77.41605400	36.04062700
H	38.09575000	78.33517000	37.00423800
H	36.85857500	79.18713100	36.06749900
H	47.60126400	80.16609000	31.34096900
C	47.07022500	80.86021700	30.67537000
C	47.99439100	81.93780500	30.14833300
C	48.35870900	83.03127100	30.95531200
C	48.52376100	81.86488400	28.84899700
C	49.22886200	84.02267700	30.48675900
C	49.39459000	82.85205500	28.37197800
C	49.74943000	83.93118500	29.18882600
H	46.24294400	81.28918300	31.25296900
H	46.64151700	80.26752300	29.86009000
H	47.95491000	83.10923200	31.96162900
H	48.25108100	81.02975800	28.20859000
H	49.49323900	84.85439400	31.13348900
H	49.79234200	82.77749800	27.36371800
H	50.42444600	84.69742000	28.81964200
C	40.44141200	76.20867700	39.65683700
C	41.02727600	76.56275600	41.00964100
O	41.70456100	75.72903600	41.69044400
C	41.44255100	75.59566600	38.63533500
C	41.86855200	74.16266700	39.00653400
C	40.80693400	75.62717800	37.23116000
H	39.63211900	75.48168200	39.82554700
H	42.33775800	76.23244900	38.61751600
H	40.54095000	76.64872900	36.93634400
H	39.89521800	75.01465500	37.20245200
H	41.50228200	75.22560600	36.48521100
H	42.33857400	74.13166800	39.99118600
H	40.99684000	73.49337000	39.02131500
H	42.57793400	73.77195300	38.26746900
N	40.73412300	77.80100300	41.48759300
C	41.33347200	78.33824600	42.71438900
C	41.08582900	77.56346700	43.99660100
O	41.84445700	77.74795500	44.98628800
H	40.48397300	78.52821700	40.80783000
H	42.41611400	78.42799500	42.59230100
N	40.05102500	76.68641700	44.05241400
C	39.88366800	75.80384800	45.20947900
C	41.07286100	74.85113100	45.44953800
O	41.30842800	74.44353200	46.60738200
H	39.45633300	76.58512400	43.24315800
H	39.78366000	76.38867400	46.12639800
N	41.78457700	74.49340700	44.34331700
C	43.08229600	73.80616500	44.42296900
C	44.24196000	74.80395200	44.27541000
O	45.26617600	74.70864700	45.00161300
C	43.14193300	72.65016300	43.39068800
C	44.48998800	71.87773700	43.31641700
C	44.22712100	70.39721300	42.96837700
C	45.47258000	72.49280800	42.29742400
H	41.50918300	74.85450200	43.43335700
H	43.16918000	73.40257900	45.43400100
H	42.33445300	71.96101100	43.67100100
H	42.88896000	73.04244200	42.39497700
H	44.96035700	71.91505700	44.31023100
H	46.41527800	71.93776200	42.26201700
H	45.73166000	73.52686200	42.53655200
H	45.04168900	72.47545900	41.28727900
H	43.59134200	69.91449800	43.72135800
H	43.72471600	70.31024000	41.99506100
H	45.16827600	69.83885300	42.90747500
N	44.10910200	75.77668900	43.33075800
C	45.19620800	76.69944800	43.02023700
C	45.67800300	77.54266500	44.19635900
O	46.85895000	78.00170000	44.18805700
H	43.28333000	75.80255700	42.73481900
H	46.07587300	76.15568700	42.65992200
H	44.85878100	77.36356700	42.21649000
N	44.82861900	77.75842900	45.22145000
C	45.24712800	78.46822400	46.43781500
C	46.39260600	77.78398900	47.21092200
O	47.02128500	78.47057000	48.05375300
C	44.03800000	78.66847000	47.36590000
H	43.85011100	77.48527400	45.13846800
H	45.66094700	79.44104600	46.14990500
H	43.60655300	77.70523900	47.65883800
H	44.36342000	79.19558200	48.26601000
H	43.25566200	79.24669100	46.86476300
N	46.65080900	76.47187800	46.95560800
C	47.78698900	75.78133300	47.55924000
C	48.92594300	75.44185500	46.58326900
O	49.79594100	74.60162500	46.91253200
H	46.07608500	75.94114200	46.30071900
H	48.18381500	76.42385700	48.35106800
N	48.91258300	76.12049400	45.40451000
C	49.86190700	75.87574500	44.31187700
C	50.56961600	77.16331500	43.85658200
O	51.81512600	77.19521000	43.69535400
C	49.11218300	75.22647000	43.11821800
C	49.94930800	75.06191500	41.83186100
C	49.05937100	74.57811200	40.69387700
O	48.49897200	73.45020200	40.72367800
N	48.88021000	75.46263700	39.67641300
H	48.14428200	76.75924400	45.22696600
H	50.63561400	75.21053400	44.69901200
H	48.72517900	74.25128500	43.43127400
H	48.24358000	75.85917500	42.89284000
H	50.73706200	74.31742100	41.99649000
H	50.43435800	76.00375000	41.55923700
H	49.30276700	76.39992000	39.64330200
H	48.29014900	75.17186100	38.91013900
N	49.75553900	78.20992400	43.54545700
C	50.26372000	79.45239400	42.93974500

C	50.89495200	80.36944700	44.00313600
C	49.10949700	80.14787300	42.19217900
C	48.54531700	79.35424200	40.99295800
C	49.51491600	79.25501100	39.80555200
O	49.87353400	80.36782600	39.23585300
O	49.91171500	78.09862100	39.43065800
H	48.75602700	78.15071800	43.71906500
H	51.04518200	79.16091300	42.22684900
H	48.29913300	80.34168400	42.91096800
H	49.46143200	81.12317400	41.83954500
H	48.26075900	78.34597600	41.30503500
H	47.63740200	79.86264500	40.63796000
C	47.15577800	82.40483800	38.72409200
C	47.98717900	83.36436200	39.56296500
O	47.49825600	84.44603300	40.00997000
C	46.42071000	83.10152500	37.55476800
C	45.46669700	82.10587000	36.85439000
C	47.42593800	83.72627300	36.57003700
C	44.51601500	82.75058100	35.83086200
H	47.78395800	81.58465200	38.35960400
H	45.81368900	83.90815400	37.98805800
H	46.06166400	81.31947300	36.36559700
H	44.86416200	81.59864400	37.61961600
H	48.05625400	84.46997700	37.07303700
H	46.91419500	84.21686300	35.73415900
H	48.08560000	82.95451700	36.15110100
H	43.82031300	82.00438400	35.42832200
H	43.92230700	83.54533800	36.29966200
H	45.05522500	83.18973600	34.98295700
N	49.26188600	82.98075300	39.81774100
C	50.11929600	83.78042400	40.69272700
C	50.80523000	84.97725600	40.02410400
O	50.68543400	86.13249400	40.49399300
H	49.55638700	82.01166000	39.56438800
H	49.53290000	84.19702700	41.51402300
N	51.59633300	84.69811400	38.93783700
C	52.44646200	85.73063600	38.35914400
C	52.27992300	85.95194700	36.86232900
O	53.09947800	86.65352500	36.22750000
H	51.83575800	83.73827900	38.68544200
H	52.22798300	86.66688000	38.88379500
N	51.18726900	85.36460100	36.26690600
C	51.07166200	85.30649400	34.80613100
C	49.97570700	86.21495600	34.23102100
O	49.77134100	86.25796400	32.99176500
C	50.90694900	83.85863300	34.31473200
H	50.67291800	84.69637200	36.83025700
H	52.01394200	85.71762200	34.42163400
H	50.84684000	83.83424600	33.22458000
H	51.75540200	83.25239600	34.64256100
H	49.99472200	83.40392200	34.71638500
N	49.27813600	86.98383500	35.11575800
C	48.37815700	88.05830600	34.68153000
C	46.89560400	87.65462500	34.57298300
O	46.39168600	87.21334200	35.87823400
C	46.59432200	86.61732300	33.49156900
H	49.56025400	86.93318000	36.08602100
H	48.72631700	88.41571400	33.70655000
H	46.32381100	88.56799500	34.37506800
H	46.83984700	86.37991800	36.13262600
H	45.52064500	86.40478900	33.47146400
H	46.90937400	86.97468000	32.50645500
H	47.13536400	85.68411100	33.67820600
H	40.14491700	87.27910400	40.75670700
C	40.71783300	87.36483800	41.68474800
S	40.40064000	85.84089700	42.74065000
H	40.37014900	88.23495300	42.24558900
H	41.77883300	87.45139400	41.44985800
C	53.77067600	81.84787300	37.16216700
O	52.98852000	82.61201700	37.77792000
N	53.77063400	80.50136200	37.22041600
C	52.79601900	79.68751700	37.97018100
C	52.22113000	78.53721200	37.11943000
C	51.54986800	79.00073400	35.84116000
C	50.37520800	79.77661700	35.90041200
C	52.08018000	78.68191700	34.57991100
C	49.76040800	80.21894500	34.72473300
C	51.46632800	79.12936200	33.40265400
C	50.30391800	79.90426500	33.47094500
H	51.98671500	80.33747100	38.30894900
H	53.01700700	77.81688300	36.87881600
H	51.49167700	78.03308100	37.76746900
H	49.96334200	80.02776500	36.87473300
H	52.97945200	78.07254600	34.51980000
H	48.85029300	80.81033000	34.78397600
H	51.89566000	78.87547800	32.43739700
H	49.83320800	80.26742200	32.56195800
H	54.45167200	80.01240500	36.65426300
C	40.56551300	85.37647700	38.61273200
C	44.51800300	84.92303800	41.38170300
C	41.98544400	82.25852400	44.54665400
C	37.96903800	82.93362800	41.90532200
C	41.86896000	85.46764400	39.08337600
C	42.93644900	86.20100000	38.41681200
C	44.03859700	86.12676500	39.23661600
C	43.65038300	85.30599900	40.37843000
C	45.37966600	86.77278500	39.07210300
C	42.81028700	86.91728700	37.10463700
H	42.32167800	87.89601900	37.21564400
C	44.18186700	84.12863700	42.47275000
C	45.12305900	83.71523500	43.49662600
C	44.40464600	82.95629900	44.40807000
C	43.02988700	82.91232300	43.91208300
C	46.58655400	84.03396600	43.49848800
C	44.85892800	82.26236200	45.60724500
C	45.97382500	82.50421300	46.32441800
C	40.67185600	82.20880300	44.10766800
C	39.60153300	81.52821600	44.81148700
C	38.45029900	81.73121800	44.06873100
C	38.83976000	82.52114700	42.90647300
C	39.77595000	80.72577600	46.06556500
C	37.09185200	81.25340400	44.31093800
C	36.51491300	81.03756900	45.50758900
C	38.32028300	83.67748800	40.79846300
C	37.40231900	84.06915100	39.74203300
C	38.14042200	84.74513500	38.80019900
C	39.51228600	84.77887200	39.28156500
C	35.93696800	83.75377900	39.73854300
C	37.68569000	85.34925000	37.50604400
H	37.89844100	86.42541100	37.46263600
N	42.31436600	84.93439300	40.27933200
N	42.91708900	83.63142200	42.73704800
N	40.18913100	82.80801900	42.95036800
N	39.60642900	84.13687400	40.51193300
Fe	41.29402900	83.74012100	41.53632900
H	42.21968600	86.33984600	36.38301100
H	43.79490900	87.08756500	36.65790600
H	44.22242300	81.44710400	45.94695300
H	36.49492200	81.05618100	43.42045900
H	36.60883600	85.22298500	37.36327700
H	38.18667000	84.88611700	36.64568400
H	46.66061100	83.30969500	46.09295800
H	46.22617900	81.88675300	47.18073100
H	37.01682300	81.25041400	46.44521800
H	35.49990400	80.65949000	45.57865900
H	35.43786600	84.18563500	38.86645200
H	35.43653900	84.14899900	40.63201100
H	35.75489000	82.67115700	39.71645100
H	45.50528700	87.19016800	38.06960400
H	45.50665000	87.59675900	39.78895900
H	46.19330900	86.06219200	39.26005300
H	38.94477400	80.02632000	46.19387700
H	40.69915000	80.13628300	46.04391600
H	39.80882100	81.36042900	46.96200500
H	47.16351100	83.18291900	43.87839000
H	46.81690700	84.89137900	44.14626400
H	46.95722900	84.27026600	42.49707100
O	41.15941300	79.95805900	39.74872100
C	42.20876100	79.52000200	38.94751200
C	41.99821000	79.35160900	37.56090500

O	40.73425700	79.69672600	37.08821600
C	40.54390500	79.79343800	35.65046300
C	43.02026100	78.84961500	36.74520100
C	44.25405200	78.49548200	37.30707100
C	44.47470800	78.64265900	38.67622700
C	43.45316900	79.15225200	39.48813600
O	43.65875600	79.25169400	40.87501500
C	44.37328800	80.46446300	41.32964500
H	42.86296600	78.72459900	35.68137300
H	45.04050300	78.10335300	36.67176000
H	45.41882500	78.36127500	39.12864400
H	41.29701200	80.88899900	40.12802100
H	40.62726500	78.81355900	35.16581600
H	39.53182100	80.17551100	35.52576500
H	41.26564900	80.48755000	35.20473300
H	44.43574200	80.37647000	42.41355800
H	45.37954600	80.48735200	40.90163600
H	43.80721000	81.35718600	41.05398400
O	41.64512800	82.32789500	40.73311500
H	53.50411900	85.49382600	38.51564400
H	50.88873600	83.11289900	41.09731100
H	48.46129300	88.87298400	35.40724600
H	35.37888500	79.43475900	39.54653300
H	51.30961700	81.27308900	43.53822100
H	50.14709500	80.67165800	44.74650800
H	51.70338100	79.83998700	44.51495200
H	45.55015300	85.23189100	41.28099400
H	42.20636600	81.75112600	45.47683500
H	36.92693600	82.65691800	42.00472800
H	40.35156800	85.83289500	37.65364200
H	46.40161600	81.96375500	39.39018300
H	39.98143800	77.10266700	39.22159400
H	40.94978200	79.35523700	42.84308000
H	38.96791800	75.22185100	45.06794400
H	54.55282200	82.25639900	36.50460400
H	53.27867300	79.27107800	38.86362900
H	47.46673100	74.83732800	48.00888700

$^{4}TS_{HA,B}$

C	35.67453800	81.21944700	41.70443100
C	35.10538300	80.03950700	40.88791600
C	37.19746600	81.16066800	41.91175700
C	35.55779900	80.02276500	39.41812500
H	35.40736900	82.16228200	41.20204500
H	34.00704600	80.07497400	40.92599600
H	35.39951400	79.09609000	41.37254800
H	37.54640700	81.99734400	42.52873600
H	37.48238800	80.23273300	42.42642000
H	37.75390000	81.20510000	40.96990400
H	35.12223100	79.17214100	38.88008800
H	36.64760400	79.95353100	39.33280700
H	35.24024100	80.94058900	38.90520000
H	46.63821300	80.44670100	28.17653300
C	46.65982700	81.37994700	27.59664300
C	48.00941400	82.05937800	27.69500600
C	48.32537200	82.87685000	28.79442200
C	48.98571300	81.87184300	26.70211600
C	49.58001100	83.48736100	28.90909900
C	50.24308300	82.47824600	26.80852000
C	50.54316700	83.28559200	27.91151800
H	45.85986500	82.01995600	27.98613300
H	46.41414300	81.12499100	26.55983700
H	47.58286400	83.04159100	29.57103800
H	48.75842500	81.24745600	25.84145700
H	49.79123600	84.11164900	29.77241600
H	50.98442500	82.32100800	26.02999900
H	51.51789900	83.75690000	27.99418600
C	39.39207500	77.47854400	41.08835800
C	40.44089300	77.74102200	42.15200100
O	41.14538700	76.81149200	42.65598100
C	39.84404300	76.58348700	39.90131200
C	40.04941400	75.11117100	40.30497800
C	38.80369700	76.69194600	38.76790000
H	38.53691000	76.99152200	41.58330000
H	40.79880200	76.97801200	39.52621300
H	38.67420600	77.73027300	38.44246200
H	37.82691400	76.31322700	39.09865100
H	39.11581000	76.09778300	37.90136300
H	40.80517500	75.01614400	41.08659900
H	39.11068100	74.67982800	40.68026600
H	40.36583500	74.51911700	39.43804400
N	40.53980100	79.02307300	42.59706900
C	41.57442500	79.43640800	43.55024800
C	41.53073900	78.76699500	44.91457300
O	42.55358200	78.78020000	45.64955600
H	40.21417900	79.77809900	41.98187400
H	42.56961700	79.25688600	43.13405700
N	40.37959100	78.16225300	45.30618400
C	40.33242800	77.35874800	46.52876800
C	41.28390300	76.14536000	46.51953800
O	41.72394800	75.70596100	47.60402500
H	39.57685000	78.21321200	44.69608000
H	40.62583600	77.95702200	47.39425000
N	41.56679400	75.61011800	45.29792200
C	42.64285900	74.62879100	45.09855400
C	43.93896100	75.31799500	44.64294700
O	45.05058900	74.95818600	45.11254900
C	42.18583900	73.50165200	44.13592600
C	43.28917800	72.47478700	43.74583700
C	42.68023600	71.06486000	43.60335700
C	44.01930500	72.87361100	42.44609200
H	41.15809400	76.02109200	44.46285100
H	42.87337800	74.20390500	46.07786200
H	41.35677700	72.99020600	44.64234000
H	41.76748800	73.95389500	43.22520500
H	44.03025900	72.44508300	44.55744400
H	44.84590600	72.19591700	42.21263300
H	44.44809200	73.87777900	42.50406100
H	43.32185100	72.85672000	41.59693600
H	42.22091800	70.72968300	44.54192600
H	41.90602200	71.05239500	42.82374000
H	43.44897300	70.33670000	43.32041300
N	43.83299900	76.31463000	43.72087000
C	45.01738700	76.95880600	43.15955900
C	45.95229500	77.57964500	44.19662900
O	47.19047000	77.66121800	43.94794800
H	42.92658400	76.54638000	43.31848200
H	45.62554100	76.24083400	42.59948400
H	44.68229900	77.73167300	42.45837500
N	45.41726700	78.01629000	45.35544400
C	46.24485400	78.55596600	46.44224700
C	47.27218700	77.56488900	47.02658800
O	48.19656900	78.03479900	47.73494300
C	45.33874800	79.08218800	47.56810600
H	44.40418700	78.04543300	45.46703600
H	46.85767200	79.37248800	46.04388900
H	44.71231100	78.27866900	47.97069400
H	45.96281000	79.47833700	48.37258900
H	44.67576000	79.87050300	47.19760200
N	47.11211800	76.23854800	46.76997800
C	48.09734100	75.24942500	47.19808200
C	48.90055200	74.60950100	46.05435600
O	49.54895000	73.55694400	46.26208300
H	46.31911500	75.90040000	46.22469200
H	48.79373900	75.74974300	47.87809800
N	48.85712600	75.27057500	44.86662400
C	49.48399200	74.77734800	43.63485900
C	50.40277900	75.82675200	42.98716000
O	51.55936100	75.52372300	42.60082800
C	48.37893700	74.34196000	42.63496200
C	48.89379700	73.95075700	41.23308900
C	47.72104600	73.72478700	40.28853500
O	46.89975500	72.78472500	40.46164200
N	47.60153400	74.63003600	39.28122700
H	48.26896400	76.09460100	44.79971400
H	50.11689600	73.93223900	43.91134700
H	47.81908800	73.50682700	43.06818800
H	47.67526500	75.17915000	42.53534900
H	49.46941700	73.01997800	41.29884600
H	49.55796100	74.72273500	40.83360300

H	48.24331300	75.42119600	39.13663600
H	46.82949500	74.51095800	38.64104700
N	49.84660300	77.05007600	42.76821400
C	50.53260500	78.10486600	42.00288800
C	51.59509000	78.81303000	42.86308500
C	49.47678500	79.09027200	41.46462500
C	48.48880000	78.48347000	40.44228600
C	49.15045900	78.13234300	39.10206400
O	49.61180600	79.12130400	38.39445400
O	49.22107100	76.90542700	38.74834400
H	48.91747700	77.26371800	43.11994500
H	51.04006600	77.60959300	41.16531100
H	48.91126500	79.49404400	42.31749200
H	49.99558300	79.93419700	40.99700200
H	48.01773900	77.58778800	40.85539500
H	47.69693300	79.21990000	40.24735900
C	47.29744300	81.61965500	38.08174200
C	48.41266600	82.43298000	38.72480000
O	48.22146100	83.62097500	39.12749500
C	46.44629400	82.43190600	37.07954000
C	45.25878500	81.57923100	36.57637700
C	47.31058300	82.95514500	35.91762300
C	44.20588500	82.36181500	35.77401400
H	47.71501700	80.72388700	37.60894700
H	46.04097500	83.29792600	37.62151300
H	45.64591700	80.74617000	35.96910300
H	44.76538400	81.11986000	37.44309200
H	48.10943000	83.60656200	36.29187500
H	46.70714200	83.52106400	35.19810200
H	47.77619700	82.12141100	35.37536300
H	43.34718300	81.72290800	35.53649500
H	43.83579400	83.21859800	36.35143500
H	44.60684100	82.74306000	34.82752300
N	49.60278300	81.80183400	38.86057000
C	50.71397800	82.45843100	39.54353200
C	51.51302600	83.45626400	38.69985700
O	51.81619600	84.58055500	39.16264200
H	49.65965400	80.77813700	38.65484000
H	50.35234000	83.02596100	40.40336700
N	51.93011200	83.02497700	37.46626100
C	52.85919700	83.83000500	36.68399800
C	52.45891700	84.10460400	35.24233200
O	53.27395100	84.63946100	34.45656100
H	51.82692600	82.05143800	37.18280200
H	52.97540700	84.78640900	37.20451200
N	51.18219000	83.76115800	34.86138900
C	50.80765200	83.76678700	33.44252200
C	49.89362500	84.93444300	33.04793100
O	49.55685000	85.10349600	31.84872000
C	50.19547500	82.41984500	33.01932100
H	50.65943100	83.17532000	35.50175600
H	51.73957400	83.93521100	32.88913400
H	49.98164400	82.42595900	31.94794800
H	50.89080000	81.60796600	33.24699100
H	49.25946500	82.22247900	33.55424300
N	49.48114000	85.76654600	34.04699200
C	48.76193100	87.01568300	33.77517000
C	47.22840600	86.90267900	33.85926300
O	46.81666900	86.52341700	35.21501000
C	46.60432800	85.96847500	32.82253200
H	49.86961200	85.60249700	34.96677700
H	49.04217200	87.35250200	32.77141900
H	46.82072400	87.91248000	33.74026600
H	47.13061700	85.61522700	35.40571300
H	45.51540100	85.96647200	32.93486500
H	46.86329500	86.28072100	31.80634000
H	46.97221300	84.94456000	32.94633000
H	42.03997900	88.07626100	41.26223900
C	42.78555200	88.09859700	42.06264200
S	42.38453900	86.74315800	43.30277300
H	42.73279700	89.05628300	42.58397400
H	43.77680300	87.94019900	41.63696400
C	53.00713800	79.86198100	35.29856500
O	52.36927100	80.79908000	35.83925900
N	52.92334100	78.55449100	35.61636700
C	52.00017000	77.98152000	36.61274100
C	51.09303900	76.88548100	36.01555900
C	50.25066700	77.36228800	34.84869500
C	49.25246300	78.33625700	35.04878100
C	50.44078800	76.85386300	33.55337400
C	48.47298200	78.78238400	33.97692100
C	49.66043600	77.30078500	32.47908000
C	48.67343000	78.26939200	32.68756400
H	51.38480400	78.78433000	37.02325000
H	51.70297800	76.02457200	35.70400100
H	50.44473800	76.55966400	36.84092300
H	49.10308400	78.73006300	36.05075500
H	51.20053700	76.09344900	33.38663700
H	47.70139300	79.52899600	34.14711200
H	49.82361900	76.89362300	31.48507000
H	48.06691000	78.61989100	31.85751400
H	53.48220100	77.90961500	35.07273100
C	41.50262300	86.08206400	39.30373500
C	45.80416100	84.83000800	41.16364000
C	43.55481600	82.96114600	45.03317000
C	39.27147000	84.44728600	43.28773000
C	42.86928700	85.88908700	39.47156600
C	43.88195900	86.32379800	38.51916300
C	45.10470100	86.03165100	39.07922900
C	44.82986200	85.37457500	40.35099200
C	46.47708300	86.33944800	38.56553100
C	43.60496300	86.98446700	37.20070500
H	43.33390500	88.04329800	37.31989300
C	45.56639000	84.16644200	42.36402500
C	46.60329200	83.57631800	43.18804800
C	45.96660300	83.04360700	44.29909600
C	44.53982400	83.31121700	44.12220700
C	48.05919500	83.53702400	42.83895400
C	46.52217700	82.31327400	45.43109700
C	47.79959800	82.31135300	45.86004400
C	42.19688400	83.21486200	44.90820800
C	41.20059100	82.82873600	45.88816700
C	39.97823800	83.26382900	45.40089800
C	40.24454700	83.89526600	44.11421900
C	41.48444300	82.05199000	47.13814600
C	38.64525700	83.12060600	45.97978000
C	38.33388000	83.08973600	47.28877200
C	39.50336900	85.03381100	42.06080000
C	38.47221000	85.58258200	41.19355200
C	39.10231900	86.03266400	40.05862600
C	40.52364800	85.76737500	40.23018700
C	37.01231500	85.60995700	41.53136000
C	38.50535800	86.67685600	38.84419400
H	38.91019200	87.68376700	38.67721800
N	43.45903500	85.32302100	40.58777000
N	44.31756200	83.98768000	42.93657700
N	41.59709500	83.86623300	43.83703700
N	40.75483000	85.16623900	41.46092100
Fe	42.52268600	84.46975600	42.14566800
H	42.77730300	86.49642800	36.67113500
H	44.48221900	86.94009000	36.54808500
H	45.81580200	81.69409900	45.98087800
H	37.82707500	83.03209800	45.26514200
H	37.41977600	86.77229100	38.93562800
H	38.70780200	86.09325600	37.93653900
H	48.57706500	82.91251600	45.40368400
H	48.09914900	81.69618500	46.70251300
H	39.07588500	83.21755800	48.06951600
H	37.30715200	82.95942400	47.61607600
H	36.42860500	86.09176000	40.74192100
H	36.82192300	86.15995100	42.46218300
H	36.60978800	84.59695800	41.66484000
H	46.45287400	86.69892000	37.53357900
H	46.95228700	87.12309400	39.17311100
H	47.13001200	85.45979900	38.61448300
H	40.57527700	81.56552300	47.50346300
H	42.22892100	81.26726000	46.96464200
H	41.85902200	82.69281500	47.94832700
H	48.50300400	82.58408400	43.14949300
H	48.62340800	84.33321400	43.34456200

H	48.22545700	83.64909600	41.76387000
O	40.65029900	81.06477500	40.65977700
C	41.25264000	80.39460300	39.60208800
C	40.53437200	80.19692300	38.40328900
O	39.25030200	80.73581300	38.36208200
C	38.57002500	80.78452500	37.07826900
C	41.10889300	79.48078800	37.34566200
C	42.39361200	78.93802100	37.47938600
C	43.11295000	79.11274600	38.66212800
C	42.53825000	79.83672000	39.71252300
O	43.24902400	79.96381900	40.92177300
C	44.05529700	81.17217500	41.07798500
H	40.56281700	79.33394900	36.42255800
H	42.82761300	78.37759300	36.65878800
H	44.10155800	78.68917000	38.79466600
H	41.20933900	81.85481600	40.98871900
H	38.32995700	79.77996500	36.71069100
H	37.64796100	81.33196900	37.26822900
H	39.17502200	81.31416200	36.33338500
H	44.65682400	81.03207400	41.97543200
H	44.66644100	81.36157600	40.18940000
H	43.30107700	82.10403200	41.24023600
O	42.31231000	82.92243100	41.38458400
H	53.84640000	83.35630400	36.63880900
H	51.39575600	81.67487300	39.89447300
H	49.09512700	87.75971000	34.50504100
H	35.17646700	81.24227700	42.68482100
H	52.13313600	79.56531400	42.27201600
H	51.12908500	79.31240800	43.72143300
H	52.31784600	78.08077600	43.23340200
H	46.82620700	84.88891800	40.81193700
H	43.87107000	82.45301400	45.93501500
H	38.24692300	84.42110200	43.63735600
H	41.18041000	86.53999100	38.37607900
H	46.64257800	81.26965100	38.89398900
H	39.02781700	78.43696300	40.70200800
H	41.48117300	80.51814500	43.68612600
H	39.30310900	77.01820100	46.67683600
H	53.71831400	80.06710200	34.48443900
H	52.58215100	77.55882800	37.44119200
H	47.61375900	74.43260200	47.74131900

^{4}TS1$_{HP,B}$

C	36.05184500	78.93873200	39.18096300
C	35.97361700	77.70573400	38.25577800
C	37.40517100	79.10774600	39.89285700
C	36.89205700	77.78834000	37.02507100
H	35.83229900	79.84160600	38.58963400
H	34.93467600	77.57415600	37.92024000
H	36.22340600	76.80689400	38.84022800
H	37.38167100	79.95382900	40.58976500
H	37.65166100	78.20985900	40.47618200
H	38.22955700	79.29677300	39.19722900
H	36.80405900	76.88868000	36.40396600
H	37.94323200	77.89910500	37.31297400
H	36.62688800	78.65207900	36.40088100
H	49.70626700	82.09141600	29.61067400
C	49.56705700	82.88761300	28.86651100
C	50.62277800	83.96369800	29.00780900
C	50.55051800	84.90671100	30.04864000
C	51.70701800	84.03385800	28.11681800
C	51.53658700	85.88711200	30.20037100
C	52.69562600	85.01474500	28.26356000
C	52.61371700	85.94406900	29.30688700
H	48.55938500	83.29318900	29.01523100
H	49.60098000	82.42041600	27.87629600
H	49.72333400	84.89117600	30.75157700
H	51.77668000	83.31519500	27.30382700
H	51.44455700	86.59316100	31.01821400
H	53.52592200	85.05199300	27.56374900
H	53.38021100	86.70472000	29.42238000
C	40.56190700	76.07782100	39.75068300
C	41.03835400	76.40624200	41.15084500
O	41.64704300	75.53290400	41.85001200
C	39.97129800	74.65397100	39.62436200
C	38.69279600	74.48993300	40.46826200
C	39.70652500	74.31842200	38.14503600
H	39.83668700	76.82713900	39.41216100
H	40.72637300	73.95855500	40.01374500
H	40.62345000	74.39933700	37.54936200
H	38.96350500	75.00222900	37.71313900
H	39.32323600	73.29668900	38.03926900
H	38.89432000	74.66087100	41.53266500
H	37.91468100	75.19492900	40.14538700
H	38.28784000	73.47576000	40.36967300
N	40.78110900	77.65187200	41.61961100
C	41.43322900	78.17465000	42.82234500
C	41.11837000	77.47209500	44.12852900
O	41.88143300	77.63131400	45.12015900
H	40.56742400	78.40560200	40.94191700
H	42.51946200	78.15029000	42.69784200
N	40.02181800	76.67502000	44.19792200
C	39.78614600	75.83381000	45.37210900
C	40.90818900	74.81154000	45.64413100
O	41.06388400	74.36409500	46.80112500
H	39.43174900	76.59598700	43.38195600
H	39.71480000	76.44343200	46.27577900
N	41.65266200	74.44616100	44.56364300
C	42.89418200	73.66721400	44.67983800
C	44.11972400	74.57162100	44.45867400
O	45.12541400	74.49164100	45.21355700
C	42.84631100	72.44419700	43.72757100
C	44.02252200	71.43746200	43.83857100
C	43.55097800	70.04208400	43.37595900
C	45.27172900	71.86403600	43.03918100
H	41.42572900	74.82793400	43.64790000
H	42.95679000	73.32539300	45.71544200
H	41.90916400	71.92553100	43.96951300
H	42.75252300	72.79426300	42.68891200
H	44.30585800	71.36449700	44.89972000
H	46.04605200	71.09033800	43.09823000
H	45.71201700	72.78802300	43.42090400
H	45.02497200	72.00557000	41.97853100
H	42.70895300	69.68282700	43.98053300
H	43.22675100	70.06934600	42.32660000
H	44.36330700	69.31018400	43.45391600
N	44.06291600	75.44075300	43.41144200
C	45.20215200	76.26459900	43.01938000
C	45.70663700	77.23285200	44.08471200
O	46.89067300	77.67318100	43.99656600
H	43.23983000	75.46832300	42.81001500
H	46.06434100	75.64121200	42.75795800
H	44.91575200	76.83371000	42.12752500
N	44.88507000	77.57107800	45.10017800
C	45.35354800	78.38172600	46.23094500
C	46.50302100	77.73913200	47.03274900
O	47.15892600	78.47759700	47.80740200
C	44.18196000	78.70073300	47.17407000
H	43.90332200	77.29948800	45.08601800
H	45.78326000	79.31123000	45.84197600
H	43.74306300	77.78141700	47.57611100
H	44.55075600	79.30624900	48.00535300
H	43.39164000	79.23978900	46.64423000
N	46.73249800	76.40506400	46.87999900
C	47.86741400	75.75256500	47.52674200
C	49.11078500	75.55385500	46.64176600
O	50.07712300	74.88828400	47.08276000
H	46.12912100	75.83200300	46.28977400
H	48.16940100	76.36366100	48.38211300
N	49.06935000	76.14356900	45.41751100
C	50.16404900	76.07968800	44.44194400
C	50.70610800	77.47587100	44.08570800
O	51.94064600	77.70626100	44.06982900
C	49.68432200	75.35010800	43.15882000
C	50.70732200	75.36534500	42.00278000
C	50.11037300	74.72983000	40.75351100
O	49.79040200	73.51327500	40.71589400
N	49.91836900	75.57854800	39.70816900
H	48.22304100	76.63156000	45.14361900
H	50.98468200	75.53792700	44.91595100

H	49.43708700	74.31346900	43.41172800
H	48.75543400	75.83251800	42.82408900
H	51.59413900	74.78869500	42.29181500
H	51.03377200	76.38625700	41.78581700
H	50.15643200	76.57828800	39.73473700
H	49.51922000	75.19391000	38.86418000
N	49.77820600	78.39313400	43.69291300
C	50.15195100	79.71090100	43.15333000
C	50.46869900	80.70369600	44.28708100
C	49.01675200	80.21831800	42.24275600
C	48.76039900	79.35323600	40.98905800
C	49.89369000	79.42180600	39.95412400
O	50.14589100	80.58156300	39.42318700
O	50.52088000	78.34926100	39.65465700
H	48.78749700	78.18140900	43.76003600
H	51.06308000	79.56050800	42.56130700
H	48.09303800	80.27307200	42.83885000
H	49.25397000	81.24043100	41.92657600
H	48.60041800	78.31019100	41.27296800
H	47.84213400	79.71027300	40.50159500
C	47.24119600	82.25505000	38.57391700
C	47.92257600	83.34779000	39.38747100
O	47.32110700	84.42343900	39.69424600
C	46.32951200	82.79785900	37.45223900
C	45.51354100	81.64692900	36.81933300
C	47.14912500	83.57177700	36.40223800
C	44.42341700	82.10927600	35.83798900
H	47.99371700	81.56491400	38.17580600
H	45.62335700	83.49912200	37.91848100
H	46.20056400	80.95283600	36.31087400
H	45.03763800	81.07031000	37.62389700
H	47.69661100	84.39830000	36.87151200
H	46.50273500	83.98445600	35.61816400
H	47.87995100	82.91171900	35.91604500
H	43.81780100	81.25738000	35.50851500
H	43.75192200	82.83281500	36.31784000
H	44.84607900	82.58345500	34.94439000
N	49.18465700	83.08309900	39.79566600
C	49.88648700	84.00142900	40.68904300
C	50.46616100	85.25659300	40.02986600
O	50.29704600	86.38405900	40.54917200
H	49.58433700	82.12979600	39.63224900
H	49.21753300	84.36734000	41.47090200
N	51.22352700	85.06384400	38.90287000
C	51.96106000	86.17489400	38.31585700
C	51.79411700	86.36407700	36.81568100
O	52.52313600	87.17342700	36.19866400
H	51.50171700	84.12878900	38.60685600
H	51.63490200	87.08622900	38.82784800
N	50.81389700	85.62858800	36.19039300
C	50.77393700	85.54618100	34.72550300
C	49.67628200	86.40775100	34.08885500
O	49.58755300	86.50926600	32.83860000
C	50.66597200	84.08494000	34.25534500
H	50.39927300	84.87727800	36.72900800
H	51.71773600	85.97704000	34.37033000
H	50.72028600	84.03979300	33.16520000
H	51.47984200	83.49533500	34.68450200
H	49.71786400	83.63561600	34.57253000
N	48.81777200	87.04303600	34.93733400
C	47.83943000	88.02741400	34.46494500
C	46.43267800	87.45385500	34.21494500
O	45.87454900	86.91432500	35.46054000
C	46.36319700	86.41411200	33.09684300
H	49.01875700	86.97076300	35.92647300
H	48.22182900	88.46135700	33.53498200
H	45.77163900	88.29542800	33.98106500
H	46.39359000	86.12947500	35.73416900
H	45.33204400	86.06608600	32.97854500
H	46.71377300	86.83369200	32.14900700
H	47.00190400	85.55366800	33.32177900
H	39.81228900	86.91851500	40.59915700
C	40.45958600	87.04918400	41.47067300
S	40.33406100	85.53416200	42.58104700
H	40.11262600	87.90377600	42.05574600
H	41.48897100	87.19784900	41.14443300
C	53.70111100	82.56848000	37.08095400
O	52.66587200	83.19885300	37.41183900
N	54.00722600	81.30340900	37.42976000
C	53.13280400	80.39873100	38.19900400
C	52.80555400	79.10233900	37.42993000
C	52.10584200	79.34128500	36.10617000
C	50.81327500	79.90157300	36.08112800
C	52.71906600	79.01742900	34.88478600
C	50.16127200	80.12551200	34.86453700
C	52.06705000	79.24289500	33.66545100
C	50.78423200	79.79971000	33.65149500
H	52.20860100	80.92601100	38.44291800
H	53.72614800	78.52373500	37.26319500
H	52.15616300	78.52275900	38.10036900
H	50.33638500	80.15692400	37.02365500
H	53.71300400	78.57559500	34.88959000
H	49.16023200	80.54915100	34.86127800
H	52.55959500	78.98398600	32.73227800
H	50.27430500	79.97576100	32.70867700
H	54.86877200	80.91875900	37.06431300
C	40.30778200	85.07134800	38.41547100
C	44.34608900	84.75714900	41.07768200
C	41.97253700	82.07686000	44.34653900
C	37.86236400	82.63494200	41.82349800
C	41.61988300	85.20581200	38.85042200
C	42.64928700	85.95078300	38.14189300
C	43.77478700	85.91982500	38.93415400
C	43.43786100	85.11144500	40.09951100
C	45.09263300	86.59744600	38.72136900
C	42.47247200	86.63266900	36.81730200
H	41.98614500	87.61318000	36.92192200
C	44.06559700	83.95740500	42.18122700
C	45.04829400	83.56897800	43.17438800
C	44.37822900	82.79181500	44.10824700
C	42.98873000	82.72355000	43.66073700
C	46.50052400	83.92892300	43.13877300
C	44.88651600	82.10026900	45.28547500
C	46.04778500	82.31643900	45.93542000
C	40.64318100	82.01348700	43.96003900
C	39.60252900	81.35248000	44.72250400
C	38.42630400	81.51747800	44.00993800
C	38.76901100	82.26713000	42.80898700
C	39.82131200	80.60583400	46.00352700
C	37.08146400	81.03460700	44.31336700
C	36.54163300	80.87631700	45.53564100
C	38.16638600	83.35656900	40.68830000
C	37.21052400	83.70569600	39.65066700
C	37.90588400	84.38166000	38.67722300
C	39.28852900	84.45709400	39.12048800
C	35.75402700	83.35436400	39.69263200
C	37.40212300	84.95441300	37.38699100
H	37.56490800	86.03879800	37.33200900
N	42.10831800	84.70965300	40.04517500
N	42.82277200	83.43160400	42.48651900
N	40.11710800	82.57001700	42.79902300
N	39.43226800	83.83778500	40.35709500
Fe	41.15361000	83.50895700	41.34119500
H	41.85581400	86.03568900	36.13451100
H	43.43860200	86.79330700	36.32864200
H	44.25082600	81.30505200	45.66941900
H	36.46497000	80.78008400	43.45142600
H	36.32983000	84.77870100	37.26448700
H	37.90840200	84.50770900	36.52120100
H	46.74532000	83.09755800	45.65868000
H	46.32983000	81.69856200	46.78229700
H	37.06650200	81.14661200	46.44551000
H	35.53503500	80.48863300	45.65621900
H	35.22774400	83.73059900	38.81094300
H	35.25991500	83.78010200	40.57568000
H	35.60086100	82.26776400	39.72653500
H	45.18173500	86.99070100	37.70544300
H	45.21065800	87.44451500	39.41244700
H	45.93063100	85.91583900	38.90827900
H	38.99992500	79.90710900	46.18656500

H	40.74762000	80.02149300	45.98289300
H	39.87441500	81.28023400	46.86927200
H	47.11639100	83.06975100	43.43035800
H	46.73420800	84.74257500	43.83938500
H	46.82244400	84.24709000	42.14433000
O	41.12911600	79.81827200	40.01371900
C	41.93195000	79.31620200	39.02839200
C	41.49789500	79.28905300	37.67751600
O	40.25210900	79.87122500	37.43750200
C	39.85356600	80.08683700	36.05933100
C	42.27622700	78.69157800	36.68039400
C	43.50740800	78.10597400	37.00954800
C	43.96649600	78.13072700	38.32737000
C	43.18660500	78.73556700	39.31960800
O	43.66083200	78.71402200	40.64746200
C	44.26842300	79.97682300	41.10960600
H	41.93368300	78.66808000	35.65300200
H	44.10559000	77.64028100	36.23385600
H	44.91694400	77.69015600	38.60598700
H	41.26274500	80.99483500	40.25966500
H	39.68270100	79.13922400	35.53425800
H	38.91848400	80.64312400	36.11760500
H	40.60528500	80.67432200	35.51897100
H	44.54656700	79.80864100	42.15017900
H	45.16509200	80.19521300	40.51980900
H	43.55228700	80.79785300	41.03132300
O	41.52593800	82.08366400	40.51668900
H	53.03825300	86.06855400	38.48877900
H	50.70408800	83.43935100	41.15523600
H	47.76434100	88.81596800	35.21989800
H	35.25317300	78.86188500	39.93378000
H	50.78645700	81.67148000	43.87788400
H	49.58648600	80.86404400	44.91930800
H	51.27592300	80.30796700	44.90981600
H	45.36652500	85.09153700	40.94208800
H	42.23060000	81.59163700	45.27874000
H	36.82940400	82.34085500	41.96024500
H	40.05761200	85.50665700	37.45547700
H	46.62326400	81.67236100	39.27381400
H	41.43643400	76.16339800	39.09004500
H	41.15044900	79.22788300	42.90799600
H	38.83661000	75.30776900	45.23531300
H	54.46689900	83.04447900	36.45053000
H	53.62418800	80.14492700	39.14624400
H	47.56927900	74.76700100	47.89438200

^{4}IM1$_{HA,B}$

C	36.14169400	79.06549300	41.46645100
C	35.10213000	78.04858200	40.95075000
C	37.60512500	78.68430200	41.18258400
C	35.09234800	77.87719700	39.42276500
H	35.93415900	80.05030600	41.02125700
H	34.10313100	78.36566800	41.28242700
H	35.28812100	77.07340100	41.42580800
H	38.28772500	79.38183800	41.68064900
H	37.81478800	77.66812300	41.54899400
H	37.84698500	78.71355300	40.11484100
H	34.29447700	77.19522600	39.10562100
H	36.04245100	77.46613000	39.06215300
H	34.93217600	78.83985000	38.92044000
H	51.91270500	86.16339100	27.25577000
C	51.22625100	87.02182900	27.25692500
C	51.12711500	87.64814400	28.63203200
C	50.19655400	87.17546500	29.57387800
C	51.97819100	88.70181600	29.00675900
C	50.11592400	87.72968200	30.85743800
C	51.90523200	89.26184700	30.28749700
C	50.97583900	88.77688700	31.21491900
H	50.25210800	86.65717500	26.91233600
H	51.60044700	87.73743300	26.51628300
H	49.52416200	86.36653300	29.29888700
H	52.69967400	89.08587700	28.28962000
H	49.39019400	87.34681900	31.57012000
H	52.57018300	90.07729500	30.55781900
H	50.91575500	89.21273500	32.20754600
C	41.31737400	76.58228200	39.46358000
C	41.73322300	77.12589000	40.81171600
O	42.32440000	76.38771900	41.66680100
C	42.52625700	76.29171300	38.51800500
C	43.39365600	75.11619500	39.00505600
C	42.00821700	76.02981400	37.08978700
H	40.77710400	75.64150700	39.63275800
H	43.14987800	77.19877600	38.48983800
H	41.46192600	76.89448600	36.69742500
H	41.33715200	75.16025400	37.07571700
H	42.84189200	75.81777700	36.41019200
H	43.76279400	75.27674100	40.02034600
H	42.81141800	74.18496000	39.00537500
H	44.25266200	74.97436600	38.33857900
N	41.46165100	78.42702100	41.07175100
C	41.99338300	79.10373100	42.25955700
C	41.56325800	78.53489400	43.60512100
O	42.27806200	78.72367800	44.62439700
H	41.09350800	79.02892600	40.31772400
H	43.08930600	79.06305200	42.26700000
N	40.40909300	77.82084400	43.66883800
C	40.01562500	77.15001400	44.90841200
C	41.01670300	76.08137100	45.38991100
O	41.09864400	75.81982400	46.60991700
H	39.83523900	77.74396300	42.84111400
H	39.93166500	77.86797500	45.72747500
N	41.74411100	75.45614300	44.41977300
C	42.89494400	74.59293800	44.72641200
C	44.20762300	75.39322000	44.68610700
O	45.11292100	75.19793600	45.53929500
C	42.90720500	73.36710900	43.77576300
C	44.14300100	72.43035500	43.90604200
C	43.72792000	70.96460600	43.66289000
C	45.28004200	72.82708000	42.94085600
H	41.62620900	75.73733800	43.45000300
H	42.78522000	74.26202900	45.76129600
H	41.98831500	72.80754800	43.99491800
H	42.82210500	73.72089800	42.73802500
H	44.52630700	72.50901500	44.93381400
H	46.16297500	72.19214400	43.06331600
H	45.61101600	73.85751800	43.09642900
H	44.94517400	72.73505500	41.89824400
H	42.97050100	70.63737400	44.38637600
H	43.30886100	70.84075900	42.65469600
H	44.59249000	70.29632000	43.74795300
N	44.33558900	76.30122200	43.68022100
C	45.56496700	77.04917400	43.47335200
C	45.98643700	77.93932000	44.63840100
O	47.20078800	78.29013600	44.73674900
H	43.59943000	76.39921800	42.98193700
H	46.40826600	76.37185100	43.29914900
H	45.44630400	77.66147000	42.57377600
N	45.06005900	78.28682600	45.54694400
C	45.40910400	78.99339800	46.78647400
C	46.46524300	78.26763600	47.64569500
O	47.08542100	78.94122500	48.50390200
C	44.13356700	79.23368000	47.61130700
H	44.07368600	78.14240100	45.33411000
H	45.87720100	79.95167700	46.53514600
H	43.65138600	78.28422800	47.86937700
H	44.39476900	79.75771300	48.53382700
H	43.41628700	79.83492200	47.04341500
N	46.65117400	76.93074200	47.45512500
C	47.69177900	76.19711900	48.17120700
C	48.84579500	75.68592100	47.29408600
O	49.57072000	74.74915400	47.70555500
H	46.07400400	76.40937900	46.79465100
H	48.09753300	76.86921400	48.93422000
N	49.01635000	76.31739500	46.09917900
C	49.97917600	75.85066700	45.09145400
C	50.70222700	76.99184300	44.35943900
O	51.93687200	76.92087400	44.13094800
C	49.24528600	74.94927500	44.05626400
C	50.11013500	74.48437100	42.86264800

C	49.22977200	73.82565500	41.80994300
O	48.69892900	72.69949700	41.99494800
N	49.01416200	74.56162400	40.68458600
H	48.35412100	77.04353300	45.84354700
H	50.74382700	75.27619900	45.61702300
H	48.84152800	74.07651900	44.57984100
H	48.38746900	75.51798000	43.67371200
H	50.84758900	73.75070600	43.20615500
H	50.65803900	75.32601200	42.42920600
H	49.41220400	75.49056400	40.51129700
H	48.41104500	74.16203800	39.98006500
N	49.91706900	77.98477800	43.86031900
C	50.42740900	79.01061700	42.93485100
C	51.23887500	80.08560500	43.68004300
C	49.22867700	79.62064200	42.17714100
C	48.46011200	78.62329400	41.27198900
C	49.23040900	78.29893400	39.99005600
O	49.24365000	79.22271100	39.07276200
O	49.85355400	77.18858200	39.89661100
H	48.93318800	78.04700800	44.11413700
H	51.09238900	78.49911800	42.22703800
H	48.53500600	80.04475800	42.91655900
H	49.59359900	80.45102400	41.55849000
H	48.26119000	77.69833600	41.81845400
H	47.49749800	79.07095000	40.99009200
C	47.20272900	80.07804700	36.53174800
C	47.12727400	81.36952700	37.33079100
O	46.44838000	82.38590800	36.94050300
C	47.15601800	80.23910000	34.99815700
C	46.90249700	78.86091300	34.33975400
C	48.44311100	80.90311600	34.47311000
C	46.69236300	78.91161200	32.81759200
H	48.08686300	79.51125500	36.84770000
H	46.30353600	80.88829000	34.75202400
H	47.74399900	78.19195300	34.57504900
H	46.01314700	78.41137600	34.80532100
H	48.63310500	81.85242700	34.98458200
H	48.38164300	81.10573800	33.39821500
H	49.30778200	80.24911500	34.64724600
H	46.43998400	77.91926600	32.42566300
H	45.87255300	79.59335300	32.55692200
H	47.59121800	79.25337200	32.29218600
N	47.74010800	81.35124600	38.52832800
C	47.51388900	82.38620800	39.52787300
C	48.26181400	83.70741300	39.35936900
O	47.82173900	84.73419400	39.94507000
H	48.31630300	80.51035000	38.80442100
H	46.45374500	82.65043400	39.57412000
N	49.40889700	83.69719900	38.63985600
C	50.20726900	84.91144000	38.47341300
C	49.95551700	85.66682500	37.16363500
O	49.88187400	86.91566500	37.14062900
H	49.73603700	82.84927100	38.18257900
H	49.97092900	85.59817700	39.28612900
N	49.86479800	84.88963400	36.03618500
C	49.75781600	85.49781900	34.71084100
C	48.31245800	85.65543200	34.21344500
O	48.08737700	86.23901000	33.12069300
C	50.59973800	84.72402400	33.68121300
H	50.13561700	83.90547700	36.10600600
H	50.13613200	86.52380100	34.79613300
H	50.50378500	85.19022800	32.69819900
H	51.65417000	84.73300100	33.97510100
H	50.26580200	83.68179900	33.60425400
N	47.31637100	85.16132800	34.99780600
C	45.91039300	85.41740700	34.68296700
C	45.15430700	84.24941600	34.02313400
O	44.81358000	83.21078400	34.99759100
C	45.87545700	83.65363600	32.81463800
H	47.56382200	84.69725400	35.86301000
H	45.89546500	86.27195400	33.99950200
H	44.17874300	84.64040200	33.71085800
H	45.59499400	82.86324000	35.51441000
H	45.25629600	82.87796600	32.35356800
H	46.09694500	84.42964200	32.07494100

H	46.82585300	83.20310900	33.11826600
H	42.52198300	87.59477900	41.90880700
C	42.19172000	87.37143500	42.92601500
S	40.65939400	86.26082000	42.88875300
H	41.92100000	88.29905400	43.43580900
H	42.99913600	86.87727100	43.47139200
C	51.69167900	81.47689100	36.05464900
O	50.94090100	82.29437300	36.66466200
N	52.26655100	80.40229200	36.60239000
C	52.07629700	79.97639000	38.00556100
C	53.12109200	78.90647100	38.38797100
C	53.12728300	77.71477000	37.44551500
C	52.09821200	76.75389600	37.50373500
C	54.13993100	77.57096300	36.47912700
C	52.09513600	75.67845400	36.60948700
C	54.12974200	76.49360500	35.58193500
C	53.10455700	75.54499000	35.64557800
H	51.05896500	79.58810000	38.14656100
H	54.12083800	79.35923300	38.41901100
H	52.88048900	78.57589600	39.40445100
H	51.31196600	76.84974100	38.25265000
H	54.95655600	78.28996100	36.44861900
H	51.30392600	74.93731400	36.67110400
H	54.92406700	76.39294200	34.84781400
H	53.09562100	74.70451700	34.95784400
H	52.78620600	79.77038800	36.00299600
C	40.51221200	85.30624000	38.65394000
C	44.58918400	85.07787500	41.29926200
C	42.16201400	82.67588100	44.75848700
C	38.06699600	83.02379800	42.17277900
C	41.82168400	85.47499300	39.08578600
C	42.84841800	86.18768600	38.33892500
C	43.99938300	86.14960800	39.10047900
C	43.67541700	85.39566400	40.30430200
C	45.32589200	86.79291800	38.82456500
C	42.62210600	86.88251200	37.02852200
H	41.95791300	87.74984700	37.14316200
C	44.29521300	84.37624900	42.46311800
C	45.26390500	84.04648600	43.49169800
C	44.57331600	83.36916700	44.48568000
C	43.18388500	83.28153300	44.04070700
C	46.72735000	84.36170700	43.42891600
C	45.06447000	82.78345400	45.72690400
C	46.18851800	83.09752100	46.40028700
C	40.83623400	82.57716700	44.36758500
C	39.80132700	81.92964600	45.15622200
C	38.62549400	82.04312600	44.43510400
C	38.96468100	82.73805600	43.19345000
C	40.03693900	81.22533800	46.45736000
C	37.28773100	81.55816600	44.75670600
C	36.75601300	81.39768900	45.98346900
C	38.38751200	83.65095700	40.98076900
C	37.43203900	83.92998700	39.91592100
C	38.12630000	84.56524200	38.91524100
C	39.50560600	84.68273800	39.37069400
C	35.97745900	83.57159900	39.96620800
C	37.62249500	85.07293400	37.59765200
H	37.76592000	86.15714600	37.50021500
N	42.33850400	85.00755500	40.28318000
N	43.03601000	83.89649600	42.81053100
N	40.30350300	83.06261500	43.18185100
N	39.64450100	84.11117800	40.62623600
Fe	41.29955900	84.11550700	41.77539200
H	42.15967300	86.22083500	36.28474900
H	43.56230300	87.24873000	36.60609300
H	44.44149200	81.99576300	46.14679600
H	36.65897800	81.31088900	43.90156300
H	36.55391900	84.87329900	37.47453400
H	38.14334900	84.60073400	36.75394900
H	46.86148400	83.88848700	46.09028500
H	46.45877200	82.56333800	47.30570800
H	37.28564200	81.66719200	46.89056800
H	35.74852300	81.01337000	46.10959500
H	35.45661600	83.88483900	39.05655000
H	35.47253400	84.05138500	40.81484100

H	35.82932600	82.48840500	40.07261500
H	45.41873000	87.09009700	37.77505900
H	45.45455800	87.70292300	39.42684300
H	46.16812700	86.13483000	39.06466000
H	39.23457200	80.51013100	46.66333400
H	40.97995100	80.66797500	46.44039500
H	40.07713100	81.92209900	47.30614200
H	47.31377600	83.55651300	43.88663900
H	46.96989500	85.28277600	43.97794400
H	47.07859900	84.49505100	42.40125900
O	41.00577900	80.34731900	39.09772500
C	41.11730400	80.41856300	37.80223300
C	40.28190000	79.64443300	36.90734000
O	39.36391500	78.82133400	37.55668300
C	38.41343100	78.08644000	36.74072200
C	40.41307400	79.73152900	35.53258400
C	41.38373600	80.58134900	34.94403200
C	42.21109500	81.35692100	35.73697900
C	42.05092000	81.26404800	37.12927900
O	42.83011400	82.03732900	38.02001000
C	43.66645200	82.98536500	37.75193200
H	39.77554500	79.14343800	34.88328000
H	41.48048100	80.61283300	33.86505300
H	42.97956400	81.98669100	35.29606400
H	41.58781900	81.49390600	40.15083000
H	38.91979400	77.35667000	36.09793500
H	37.76833800	77.56911300	37.44903600
H	37.81625800	78.76862900	36.12459800
H	44.12758800	83.43598900	38.61939000
H	43.90010900	83.28024600	36.72750000
H	42.92653500	82.02789800	40.91559300
O	41.95416000	82.00227700	40.93861700
H	51.26783500	84.64004100	38.52435100
H	47.79761900	81.97112300	40.50148300
H	45.38867900	85.69476000	35.60499700
H	36.00636700	79.18328500	42.55110200
H	51.64495300	80.82305600	42.97574800
H	50.60832000	80.60992800	44.40815600
H	52.07093400	79.61472900	44.21031200
H	45.61437500	85.39147100	41.14777700
H	42.41671000	82.25113500	45.72025800
H	37.03220600	82.74012000	42.31886700
H	40.25132400	85.72068400	37.68717400
H	46.33263900	79.48052000	36.84893800
H	40.63664700	77.28526900	38.97310200
H	41.71707800	80.16258500	42.19023400
H	39.03540200	76.68916900	44.75236200
H	51.92301200	81.62251000	34.99178400
H	52.18447800	80.85913100	38.64520600
H	47.27245500	75.32107400	48.67384400

^{4}IM1$_{HP,B}$

C	36.20894300	78.45830600	40.32690700
C	35.89961200	77.37164800	39.27510500
C	37.09778600	77.97573300	41.48700800
C	37.12081500	76.92046000	38.45535200
H	36.69680300	79.30965300	39.82882000
H	35.12883300	77.75463400	38.59025300
H	35.45272400	76.50172400	39.78169600
H	37.25487000	78.77418200	42.22260300
H	36.61039600	77.13473700	42.00600100
H	38.08182600	77.64718200	41.13470000
H	36.83151500	76.17913200	37.70019600
H	37.89981000	76.47175100	39.08091200
H	37.56943200	77.77308500	37.92908300
H	52.99038400	87.85721200	27.39481000
C	52.69646200	88.89933800	27.58297700
C	53.01173100	89.31382800	29.00505500
C	52.15848700	88.95608700	30.06406200
C	54.17308200	90.04616600	29.30269200
C	52.45237000	89.31006500	31.38614500
C	54.47619300	90.40437700	30.62187200
C	53.61822900	90.03678700	31.66444500
H	51.62320200	88.97375900	27.37241900
H	53.22727800	89.52461900	26.85653200
H	51.25158000	88.39503200	29.85268500
H	54.84190100	90.33742500	28.49617700
H	51.77530100	89.02476900	32.18702200
H	55.37878100	90.97153900	30.83237500
H	53.85084200	90.31547700	32.68777300
C	39.23457600	73.55297700	41.91604700
C	39.90767100	74.51805600	42.86439100
O	41.10181600	74.35940100	43.25446400
C	39.67976300	72.08538900	42.08728100
C	39.23154200	71.50482300	43.44232900
C	39.15258800	71.23463500	40.91677000
H	38.14210600	73.63059700	42.01067200
H	40.77721000	72.07562900	42.05748200
H	39.49983300	71.63054800	39.95528300
H	38.05433300	71.22353500	40.89984200
H	39.49607300	70.19715800	41.00244200
H	39.65327800	72.07352600	44.27964600
H	38.13644300	71.51847500	43.53165900
H	39.56253000	70.46545700	43.55153300
N	39.16953100	75.58423700	43.29654600
C	39.81401200	76.76012700	43.89575300
C	40.48816400	76.49285700	45.23305600
O	41.41568200	77.24142300	45.63769500
H	38.29666400	75.75835400	42.81559100
H	40.56261400	77.17362200	43.21147400
N	40.04211500	75.44129200	45.97089500
C	40.70227100	75.06445100	47.22072900
C	42.15284200	74.57753300	47.04509000
O	42.97095600	74.72591100	47.98047100
H	39.29147500	74.87894500	45.59598800
H	40.75398200	75.92053200	47.89699200
N	42.44532400	73.97712700	45.85774300
C	43.82293600	73.64373200	45.46403700
C	44.48981300	74.84507500	44.77307900
O	45.68310400	75.15578200	45.03193200
C	43.82986800	72.35105200	44.61088900
C	45.22556400	71.91431000	44.08187900
C	45.32263800	70.37506100	44.04676500
C	45.52834200	72.49924900	42.68791000
H	41.74418800	73.95245600	45.12288700
H	44.39608100	73.48643700	46.38034600
H	43.41035500	71.56501900	45.25284300
H	43.13842400	72.47474100	43.76512500
H	45.98822100	72.28601100	44.78218300
H	46.52835200	72.22758800	42.33778300
H	45.48006400	73.59105500	42.67509700
H	44.80851300	72.12487900	41.94764600
H	45.18566400	69.94089300	45.04531300
H	44.55430200	69.95175100	43.38510900
H	46.29995000	70.05636200	43.66673400
N	43.72357100	75.55776500	43.90495300
C	44.23526600	76.73391300	43.20626800
C	44.84032500	77.79107700	44.13211900
O	45.79795300	78.51193900	43.72482800
H	42.78879400	75.23382300	43.66740200
H	45.01455200	76.46466000	42.48799800
H	43.40222900	77.17318700	42.64950300
N	44.31830600	77.89407000	45.37225800
C	44.86330300	78.77444000	46.41351300
C	46.38751400	78.65605600	46.60535500
O	47.02137400	79.66819100	46.99515000
C	44.14370600	78.47528900	47.74293400
H	43.45046700	77.40425700	45.57891300
H	44.70280400	79.82225500	46.13689400
H	44.27520500	77.42639400	48.02998900
H	44.55256200	79.11361100	48.53067500
H	43.07053300	78.67129300	47.65057600
N	46.95750300	77.43376200	46.41854500
C	48.39343500	77.22329800	46.58337300
C	49.16391600	76.94004500	45.28607900
O	50.31963200	76.45868600	45.34176500
H	46.40204800	76.64538200	46.08577300
H	48.80656500	78.12709400	47.04174100
N	48.51624000	77.26219300	44.13357000

C	49.05270700	76.98390800	42.79986400
C	49.29696200	78.25371200	41.97039300
O	50.27134200	78.30551800	41.17127000
C	48.06656700	76.04377500	42.05481600
C	48.48507200	75.64021200	40.62352500
C	47.34178700	74.89987300	39.94334500
O	47.18934400	73.65572100	40.06469500
N	46.46405200	75.68013200	39.25559900
H	47.56107900	77.59959000	44.19787700
H	50.02435100	76.50446900	42.93558600
H	47.92547300	75.14631500	42.66686100
H	47.09909400	76.55910500	42.00816500
H	49.35002700	74.97146800	40.66056000
H	48.76810600	76.52652900	40.04882400
H	46.56801700	76.68868900	39.08950400
H	45.67138400	75.21736200	38.83493000
N	48.36709900	79.23796000	42.07065100
C	48.43336800	80.47195800	41.26599000
C	49.31553100	81.52745800	41.95656600
C	47.01309600	80.99262700	40.98615800
C	46.10628600	80.01348200	40.22008600
C	46.60580500	79.63294500	38.81393900
O	47.09119600	80.56511400	38.07294200
O	46.48456900	78.40374600	38.44467600
H	47.59368300	79.14227600	42.72052500
H	48.90027600	80.20048400	40.31555400
H	46.53119600	81.26508100	41.93575500
H	47.11320100	81.91462600	40.40465300
H	45.95741200	79.09710900	40.79806200
H	45.11996600	80.49036000	40.10432400
C	47.37355100	82.40542800	35.05992900
C	47.53100400	83.50944500	36.09041900
O	47.77969900	84.72132000	35.75795000
C	48.18812600	82.61601600	33.76546300
C	47.58539000	81.77262800	32.61593700
C	49.67975900	82.30449500	33.99524500
C	48.23375600	82.01800800	31.24349600
H	47.59308800	81.43624700	35.52409800
H	48.09292000	83.67711000	33.49305100
H	47.66459500	80.70682400	32.87456500
H	46.51041400	81.99811900	32.54559300
H	50.07148500	82.85559400	34.85578300
H	50.28497400	82.57137300	33.12115800
H	49.82257200	81.23445400	34.19437400
H	47.71136500	81.45856800	30.45765800
H	48.19895900	83.08231500	30.97629900
H	49.28323500	81.70384500	31.22919000
N	47.32538200	83.16510600	37.37677600
C	47.18073000	84.18262500	38.40846900
C	48.45660000	84.89694100	38.84312900
O	48.36830900	85.96644800	39.51102400
H	47.20120300	82.16856000	37.64924700
H	46.49774000	84.96702700	38.06914200
N	49.63897900	84.34169400	38.48892400
C	50.90290500	85.00519400	38.76683500
C	51.41118600	85.93196600	37.65925700
O	52.46364400	86.58856400	37.84065000
H	49.68926000	83.43467500	38.02584400
H	50.80436700	85.61142600	39.67087500
N	50.68026700	85.99330500	36.50312800
C	51.15205000	86.78134500	35.36049300
C	50.08221900	87.72851400	34.80167100
O	50.28338200	88.34844900	33.72398600
C	51.71851700	85.88690400	34.24178300
H	49.88827200	85.36770300	36.37264000
H	51.96232100	87.40640300	35.75553000
H	52.05104600	86.50233100	33.40246300
H	52.56559000	85.31091100	34.62616600
H	50.95648700	85.18736500	33.88071500
N	48.94062800	87.86836100	35.52350900
C	47.82190800	88.69560300	35.07626600
C	46.67687400	87.87099600	34.45344600
O	46.12115300	86.95732800	35.44539200
C	47.08069700	87.11962600	33.18288900
H	48.83594600	87.33279500	36.37403000
H	48.21328800	89.40980000	34.34633200
H	45.86013600	88.56785600	34.22871900
H	46.69402800	86.15183400	35.54053200
H	46.21284800	86.59567900	32.76893900
H	47.48021100	87.80428200	32.42750800
H	47.85248000	86.37740900	33.40981400
H	43.39554100	87.32788000	42.63421900
C	42.64102300	87.02616500	43.36718300
S	41.24124200	86.14149100	42.47643600
H	42.22445700	87.91460600	43.84633600
H	43.09820400	86.36279100	44.10204800
C	51.64405400	81.20198200	36.99928500
O	50.83556900	82.11709700	37.29283800
N	51.45774400	79.88634500	37.21698100
C	50.26663400	79.29947200	37.86102200
C	49.96651400	77.88572200	37.32075800
C	49.57349900	77.82023700	35.85779200
C	48.23578900	78.04432500	35.47803100
C	50.51765000	77.52502300	34.85967600
C	47.86390300	77.97409700	34.13063600
C	50.14625200	77.46087600	33.51098400
C	48.81525800	77.68488600	33.14227500
H	49.41824200	79.96559500	37.68419900
H	50.83645500	77.23831000	37.50651200
H	49.14548100	77.49518100	37.93026600
H	47.49899500	78.25907200	36.24924800
H	51.54945100	77.32483900	35.14105100
H	46.82682900	78.13874600	33.85180700
H	50.89056700	77.22870600	32.75438600
H	48.52085400	77.62884500	32.09821000
H	52.20624100	79.25763700	36.95762500
C	41.06813200	85.32223500	38.51956400
C	45.09786200	85.73732600	41.18265000
C	43.28221700	82.46478400	44.27071500
C	38.96713900	82.78596300	42.07740800
C	42.32738000	85.70422800	38.95115400
C	43.22021000	86.60521100	38.23430900
C	44.32391900	86.79672800	39.03342100
C	44.13598800	85.96363900	40.21377500
C	45.49924200	87.68817800	38.76633700
C	42.97130400	87.20307200	36.88157800
H	42.60997300	88.23894000	36.95379000
C	44.98565900	84.82083300	42.23068300
C	46.06771400	84.46055800	43.13072500
C	45.57028400	83.47246700	43.96616900
C	44.17213800	83.27518400	43.58567200
C	47.45420800	85.02769600	43.08350100
C	46.25057400	82.67110000	44.98053300
C	47.28757300	83.03612100	45.75732400
C	41.92600700	82.30957100	43.99585600
C	41.00285800	81.53536600	44.80413800
C	39.75963000	81.64292600	44.19417800
C	39.95253000	82.48558900	43.01651300
C	41.34851800	80.80658300	46.06565100
C	38.47678800	81.08462800	44.59502300
C	38.24622100	80.05272900	45.43499600
C	39.16575900	83.48200800	40.89963000
C	38.15979200	83.65957400	39.85943700
C	38.77178500	84.33405600	38.83137900
C	40.14436600	84.59124500	39.25569300
C	36.74933200	83.15958200	39.94812200
C	38.19802800	84.76281800	37.51458500
H	38.22334900	85.85426900	37.39620700
N	42.89746700	85.32069600	40.16011900
N	43.84419900	84.09520700	42.51829500
N	41.26598100	82.87745300	42.91921500
N	40.36322200	84.06288900	40.51108300
Fe	42.10842400	84.02395300	41.48961200
H	42.22354800	86.63672900	36.31639700
H	43.89625400	87.20931000	36.29225800
H	45.88797700	81.65105700	45.09748600
H	37.60324900	81.57355500	44.16474300
H	37.15576600	84.44703600	37.41068800
H	38.75551400	84.33386000	36.67164400
H	47.71304700	84.03458900	45.74055700

H	47.71657000	82.31961300	46.44992500
H	39.04165000	79.48633900	45.90528100
H	37.23067500	79.75477200	45.67765200
H	36.17029100	83.44108500	39.06376900
H	36.22803500	83.56817000	40.82392600
H	36.70978700	82.06474500	40.02948900
H	45.33006400	88.69676100	39.17189100
H	46.42431400	87.30352300	39.20793500
H	45.67010100	87.77992400	37.68875900
H	41.47257300	79.72864500	45.89402600
H	42.27433300	81.18165500	46.51150600
H	40.55367800	80.92530500	46.81208900
H	48.18266300	84.28791800	43.43141000
H	47.55708000	85.90842500	43.73358900
H	47.74408300	85.33562700	42.07350400
O	40.07135200	76.18245900	40.46427100
C	41.11842800	76.50360700	39.79148300
C	41.56905400	75.71340500	38.65254000
O	40.79303700	74.60332200	38.40332000
C	41.15764700	73.73488600	37.29308300
C	42.68841300	76.07788700	37.91768000
C	43.41247400	77.23259900	38.27927800
C	43.02701000	78.03018700	39.36674400
C	41.91482500	77.67925100	40.12268600
O	41.48964600	78.35322800	41.23348100
C	42.11888000	79.64394700	41.58193600
H	43.02207900	75.48875200	37.07255600
H	44.31174000	77.51191800	37.73951100
H	43.62127500	78.89731300	39.61466600
H	43.55471200	82.71264100	40.18158900
H	42.15600800	73.30930800	37.44097800
H	40.40992700	72.94330700	37.29560800
H	41.12317700	74.27966400	36.34346100
H	41.57347600	79.98068800	42.45888700
H	43.17520300	79.49720100	41.82725100
H	42.02129400	80.37978000	40.78041900
O	42.66736100	82.54328400	40.56447000
H	51.66992000	84.24554500	38.93521100
H	46.73265000	83.71438400	39.28918900
H	47.42817100	89.24524000	35.93753900
H	35.26089300	78.83910700	40.73422700
H	49.39712200	82.42627400	41.33344400
H	48.89364300	81.81043200	42.92832700
H	50.32137100	81.12713500	42.11674900
H	46.04457200	86.25031900	41.06646300
H	43.67581500	81.93079100	45.12656100
H	37.97361800	82.38954600	42.24980800
H	40.75031700	85.67949600	37.54738000
H	46.30075300	82.38621000	34.80789100
H	39.49099500	73.91294000	40.90899400
H	39.04952100	77.53173100	44.03800100
H	40.11002700	74.27979000	47.70130600
H	52.60453400	81.43402400	36.51521200
H	50.43304700	79.22661100	38.94307500
H	48.58441900	76.38117300	47.25491300

$^{4}IM2_{B}$

C	36.14169400	79.06549300	41.46645100
C	35.10213000	78.04858200	40.95075000
C	37.60512500	78.68430200	41.18258400
C	35.09234800	77.87719700	39.42276500
H	35.93415900	80.05030600	41.02125700
H	34.10313100	78.36566800	41.28242700
H	35.28812100	77.07340100	41.42580800
H	38.28772500	79.38183800	41.68064900
H	37.81478800	77.66812300	41.54899400
H	37.84698500	78.71355300	40.11484100
H	34.29447700	77.19522600	39.10562100
H	36.04245100	77.46613000	39.06215300
H	34.93217600	78.83985000	38.92044000
H	51.91270500	86.16339100	27.25577000
C	51.22625100	87.02182900	27.25692500
C	51.12711500	87.64814400	28.63203200
C	50.19655400	87.17546500	29.57387800
C	51.97819100	88.70181600	29.00675900
C	50.11592400	87.72968200	30.85743800
C	51.90523200	89.26184700	30.28749700
C	50.97583900	88.77688700	31.21491900
H	50.25210800	86.65717500	26.91233600
H	51.60044700	87.73743300	26.51628300
H	49.52416200	86.36653300	29.29888700
H	52.69967400	89.08587700	28.28962000
H	49.39019400	87.34681900	31.57012000
H	52.57018300	90.07729500	30.55781900
H	50.91575500	89.21273500	32.20754600
C	41.31737400	76.58228200	39.46358000
C	41.73322300	77.12589000	40.81171600
O	42.32440000	76.38771900	41.66680100
C	42.52625700	76.29171300	38.51800500
C	43.39365600	75.11619500	39.00505600
C	42.00821700	76.02981400	37.08978700
H	40.77710400	75.64150700	39.63275800
H	43.14987800	77.19877600	38.48983800
H	41.46192600	76.89448600	36.69742500
H	41.33715200	75.16025400	37.07571700
H	42.84189200	75.81777700	36.41019200
H	43.76279400	75.27674100	40.02034600
H	42.81141800	74.18496000	39.00537500
H	44.25266200	74.97436600	38.33857900
N	41.46165100	78.42702100	41.07175100
C	41.99338300	79.10373100	42.25955700
C	41.56325800	78.53489400	43.60512100
O	42.27806200	78.72367800	44.62439700
H	41.09350800	79.02892600	40.31772400
H	43.08930600	79.06305200	42.26700000
N	40.40909300	77.82084400	43.66883800
C	40.01562500	77.15001400	44.90841200
C	41.01670300	76.08137100	45.38991100
O	41.09864400	75.81982400	46.60991700
H	39.83523900	77.74396300	42.84111400
H	39.93166500	77.86797500	45.72747500
N	41.74411100	75.45614300	44.41977300
C	42.89494400	74.59293800	44.72641200
C	44.20762300	75.39322000	44.68610700
O	45.11292100	75.19793600	45.53929500
C	42.90720500	73.36710900	43.77576300
C	44.14300100	72.43035500	43.90604200
C	43.72792000	70.96460600	43.66289000
C	45.28004200	72.82708000	42.94085600
H	41.62620900	75.73733800	43.45000300
H	42.78522000	74.26202900	45.76129600
H	41.98831500	72.80754800	43.99491800
H	42.82210500	73.72089800	42.73802500
H	44.52630700	72.50901500	44.93381400
H	46.16297500	72.19214400	43.06331600
H	45.61101600	73.85751800	43.09642900
H	44.94517400	72.73505500	41.89824400
H	42.97050100	70.63737400	44.38637600
H	43.30886100	70.84075900	42.65469600
H	44.59249000	70.29632000	43.74795300
N	44.33558900	76.30122200	43.68022100
C	45.56496700	77.04917400	43.47335200
C	45.98643700	77.93932000	44.63840100
O	47.20078800	78.29013600	44.73674900
H	43.59943000	76.39921800	42.98193700
H	46.40826600	76.37185100	43.29914900
H	45.44630400	77.66147000	42.57377600
N	45.06005900	78.28682600	45.54694400
C	45.40910400	78.99339800	46.78647400
C	46.46524300	78.26763600	47.64569500
O	47.08542100	78.94122500	48.50390200
C	44.13356700	79.23368000	47.61130700
H	44.07368600	78.14240100	45.33411000
H	45.87720100	79.95167700	46.53514600
H	43.65138600	78.28422800	47.86937700
H	44.39476900	79.75771300	48.53382700
H	43.41628700	79.83492200	47.04341500
N	46.65117400	76.93074200	47.45512500
C	47.69177900	76.19711900	48.17120700

C	48.84579500	75.68592100	47.29408600
O	49.57072000	74.74915400	47.70555500
H	46.07400400	76.40937900	46.79465100
H	48.09753300	76.86921400	48.93422000
N	49.01635000	76.31739500	46.09917900
C	49.97917600	75.85066700	45.09145400
C	50.70222700	76.99184300	44.35943900
O	51.93687200	76.92087400	44.13094800
C	49.24528600	74.94927500	44.05626400
C	50.11013500	74.48437100	42.86264800
C	49.22977200	73.82565500	41.80994300
O	48.69892900	72.69949700	41.99494800
N	49.01416200	74.56162400	40.68458600
H	48.35412100	77.04353300	45.84354700
H	50.74382700	75.27619900	45.61702300
H	48.84152800	74.07651900	44.57984100
H	48.38746900	75.51798000	43.67371200
H	50.84758900	73.75070600	43.20615500
H	50.65803900	75.32601200	42.42920600
H	49.41220400	75.49056400	40.51129700
H	48.41104500	74.16203800	39.98006500
N	49.91706900	77.98477800	43.86031900
C	50.42740900	79.01061700	42.93485100
C	51.23887500	80.08560500	43.68004300
C	49.22867700	79.62064200	42.17714100
C	48.46011200	78.62329400	41.27198900
C	49.23040900	78.29893400	39.99005600
O	49.24365000	79.22271100	39.07276200
O	49.85355400	77.18858200	39.89661100
H	48.93318800	78.04700800	44.11413700
H	51.09238900	78.49911800	42.22703800
H	48.53500600	80.04475800	42.91655900
H	49.59359900	80.45102400	41.55849000
H	48.26119000	77.69833600	41.81845400
H	47.49749800	79.07095000	40.99009200
C	47.20272900	80.07804700	36.53174800
C	47.12727400	81.36952700	37.33079100
O	46.44838000	82.38590800	36.94050300
C	47.15601800	80.23910000	34.99815700
C	46.90249700	78.86091300	34.33975400
C	48.44311100	80.90311600	34.47311000
C	46.69236300	78.91161200	32.81759200
H	48.08686300	79.51125500	36.84770000
H	46.30353600	80.88829000	34.75202400
H	47.74399900	78.19195300	34.57504900
H	46.01314700	78.41137600	34.80532100
H	48.63310500	81.85242700	34.98458200
H	48.38164300	81.10573800	33.39821500
H	49.30778200	80.24911500	34.64724600
H	46.43998400	77.91926600	32.42566300
H	45.87255300	79.59335300	32.55692200
H	47.59121800	79.25337200	32.29218600
N	47.74010800	81.35124600	38.52832800
C	47.51388900	82.38620800	39.52787300
C	48.26181400	83.70741300	39.35936900
O	47.82173900	84.73419400	39.94507000
H	48.31630300	80.51035000	38.80442100
H	46.45374500	82.65043400	39.57412000
N	49.40889700	83.69719900	38.63985600
C	50.20726900	84.91144000	38.47341300
C	49.95551700	85.66682500	37.16363500
O	49.88187400	86.91566500	37.14062900
H	49.73603700	82.84927100	38.18257900
H	49.97092900	85.59817700	39.28612900
N	49.86479800	84.88963400	36.03618500
C	49.75781600	85.49781900	34.71084100
C	48.31245800	85.65543200	34.21344500
O	48.08737700	86.23901000	33.12069300
C	50.59973800	84.72402400	33.68121300
H	50.13561700	83.90547700	36.10600600
H	50.13613200	86.52380100	34.79613300
H	50.50378500	85.19022800	32.69819900
H	51.65417000	84.73300100	33.97510100
H	50.26580200	83.68179900	33.60425400
N	47.31637100	85.16132800	34.99780600
C	45.91039300	85.41740700	34.68296700
C	45.15430700	84.24941600	34.02313400
O	44.81358000	83.21078400	34.99759100
C	45.87545700	83.65363600	32.81463800
H	47.56382200	84.69725400	35.86301000
H	45.89546500	86.27195400	33.99950200
H	44.17874300	84.64040200	33.71085800
H	45.59499400	82.86324000	35.51441000
H	45.25629600	82.87796600	32.35356800
H	46.09694500	84.42964200	32.07494100
H	46.82585300	83.20310900	33.11826600
H	42.52198300	87.59477900	41.90880700
C	42.19172000	87.37143500	42.92601500
S	40.65939400	86.26082000	42.88875300
H	41.92100000	88.29905400	43.43580900
H	42.99913600	86.87727100	43.47139200
C	51.69167900	81.47689100	36.05464900
O	50.94090100	82.29437300	36.66466200
N	52.26655100	80.40229200	36.60239000
C	52.07629700	79.97639000	38.00556100
C	53.12109200	78.90647100	38.38797100
C	53.12728300	77.71477000	37.44551500
C	52.09821200	76.75389600	37.50373500
C	54.13993100	77.57096300	36.47912700
C	52.09513600	75.67845400	36.60948700
C	54.12974200	76.49360500	35.58193500
C	53.10455700	75.54499000	35.64557800
H	51.05896500	79.58810000	38.14656100
H	54.12083800	79.35923300	38.41901100
H	52.88048900	78.57589600	39.40445100
H	51.31196600	76.84974100	38.25265000
H	54.95655600	78.28996100	36.44861900
H	51.30392600	74.93731400	36.67110400
H	54.92406700	76.39294200	34.84781400
H	53.09562100	74.70451700	34.95784400
H	52.78620600	79.77038800	36.00299600
C	40.51221200	85.30624000	38.65394000
C	44.58918400	85.07787500	41.29926200
C	42.16201400	82.67588100	44.75848700
C	38.06699600	83.02379800	42.17277900
C	41.82168400	85.47499300	39.08578600
C	42.84841800	86.18768600	38.33892500
C	43.99938300	86.14960800	39.10047900
C	43.67541700	85.39566400	40.30430200
C	45.32589200	86.79291800	38.82456500
C	42.62210600	86.88251200	37.02852200
H	41.95791300	87.74984700	37.14316200
C	44.29521300	84.37624900	42.46311800
C	45.26390500	84.04648600	43.49169800
C	44.57331600	83.36916700	44.48568000
C	43.18388500	83.28153300	44.04070700
C	46.72735000	84.36170700	43.42891600
C	45.06447000	82.78345400	45.72690400
C	46.18851800	83.09752100	46.40028700
C	40.83623400	82.57716700	44.36758500
C	39.80132700	81.92964600	45.15622200
C	38.62549400	82.04312600	44.43510400
C	38.96468100	82.73805600	43.19345000
C	40.03693900	81.22533800	46.45736000
C	37.28773100	81.55816600	44.75670600
C	36.75601300	81.39768900	45.98346900
C	38.38751200	83.65095700	40.98076900
C	37.43203900	83.92998700	39.91592100
C	38.12630000	84.56524200	38.91524100
C	39.50560600	84.68273800	39.37069400
C	35.97745900	83.57159900	39.96620800
C	37.62249500	85.07293400	37.59765200
H	37.76592000	86.15714600	37.50021500
N	42.33850400	85.00755500	40.28318000
N	43.03601000	83.89649600	42.81053100
N	40.30350300	83.06261500	43.18185100
N	39.64450100	84.11117800	40.62623600
Fe	41.29955900	84.11550700	41.77539200
H	42.15967300	86.22083500	36.28474900
H	43.56230300	87.24873000	36.60609300

H	44.44149200	81.99576300	46.14679600
H	36.65897800	81.31088900	43.90156300
H	36.55391900	84.87329900	37.47453400
H	38.14334900	84.60073400	36.75394900
H	46.86148400	83.88848700	46.09028500
H	46.45877200	82.56333800	47.30570800
H	37.28564200	81.66719200	46.89056800
H	35.74852300	81.01337000	46.10959500
H	35.45661600	83.88483900	39.05655000
H	35.47253400	84.05138500	40.81484100
H	35.82932600	82.48840500	40.07261500
H	45.41873000	87.09009700	37.77505900
H	45.45455800	87.70292300	39.42684300
H	46.16812700	86.13483000	39.06466000
H	39.23457200	80.51013100	46.66333400
H	40.97995100	80.66797500	46.44039500
H	40.07713100	81.92209900	47.30614200
H	47.31377600	83.55651300	43.88663900
H	46.96989500	85.28277600	43.97794400
H	47.07859900	84.49505100	42.40125900
O	41.00577900	80.34731900	39.09772500
C	41.11730400	80.41856300	37.80223300
C	40.28190000	79.64443300	36.90734000
O	39.36391500	78.82133400	37.55668300
C	38.41343100	78.08644000	36.74072200
C	40.41307400	79.73152900	35.53258400
C	41.38373600	80.58134900	34.94403200
C	42.21109500	81.35692100	35.73697900
C	42.05092000	81.26404800	37.12927900
O	42.83011400	82.03732900	38.02001000
C	43.66645200	82.98536500	37.75193200
H	39.77554500	79.14343800	34.88328000
H	41.48048100	80.61283300	33.86505300
H	42.97956400	81.98669100	35.29606400
H	41.58781900	81.49390600	40.15083000
H	38.91979400	77.35667000	36.09793500
H	37.76833800	77.56911300	37.44903600
H	37.81625800	78.76862900	36.12459800
H	44.12758800	83.43598900	38.61939000
H	43.90010900	83.28024600	36.72750000
H	42.92653500	82.02789800	40.91559300
O	41.95416000	82.00227700	40.93861700
H	51.26783500	84.64004100	38.52435100
H	47.79761900	81.97112300	40.50148300
H	45.38867900	85.69476000	35.60499700
H	36.00636700	79.18328500	42.55110200
H	51.64495300	80.82305600	42.97574800
H	50.60832000	80.60992800	44.40815600
H	52.07093400	79.61472900	44.21031200
H	45.61437500	85.39147100	41.14777700
H	42.41671000	82.25113500	45.72025800
H	37.03220600	82.74012000	42.31886700
H	40.25132400	85.72068400	37.68717400
H	46.33263900	79.48052000	36.84893800
H	40.63664700	77.28526900	38.97310200
H	41.71707800	80.16258500	42.19023400
H	39.03540200	76.68916900	44.75236200
H	51.92301200	81.62251000	34.99178400
H	52.18447800	80.85913100	38.64520600
H	47.27245500	75.32107400	48.67384400

$^{4}Pr_{rc,B}$

C	36.69251900	77.64133100	45.39916200
C	35.18986500	77.72215200	45.05550100
C	37.59455700	78.50271700	44.49889600
C	34.55641100	79.10016500	45.31336700
H	36.83711300	77.93229100	46.45028900
H	34.65079400	76.96628000	45.64460100
H	35.04888800	77.44667200	43.99900900
H	38.65304100	78.35319800	44.74021500
H	37.45376500	78.24231100	43.44122100
H	37.37836500	79.57166200	44.60599400
H	33.48195900	79.08827200	45.09289500
H	35.01094900	79.88286600	44.69562500
H	34.67664700	79.39289200	46.36476400
H	50.20109100	84.38760000	25.81233000
C	49.66101800	85.34036800	25.72104700
C	49.96152500	86.25979300	26.88627400
C	49.33247700	86.07293400	28.13013800
C	50.88898200	87.30721100	26.75897900
C	49.61984800	86.90072700	29.22199400
C	51.18343500	88.14027500	27.84534300
C	50.55125000	87.93843500	29.07722200
H	48.59279800	85.10022700	25.66504700
H	49.95554500	85.79283700	24.76776400
H	48.60667800	85.27207100	28.24741600
H	51.38109500	87.47098000	25.80324100
H	49.12016100	86.73120600	30.17232700
H	51.90242600	88.94622800	27.72693600
H	50.77700700	88.58514800	29.91973500
C	41.53363800	76.42346000	38.12366600
C	41.68359700	76.65541100	39.61188300
O	42.37827100	75.87794000	40.34074100
C	42.83195400	76.73443000	37.31304200
C	43.94970400	75.71070500	37.58440600
C	42.49500200	76.79653200	35.81053800
H	41.26868700	75.37015900	37.96718600
H	43.18439800	77.73070200	37.62080800
H	41.76352500	77.58391900	35.59643000
H	42.08411700	75.83865000	35.46469800
H	43.39581100	77.00626200	35.22269100
H	44.17534100	75.62822200	38.65037000
H	43.65205700	74.71521000	37.22876900
H	44.86557400	75.99604600	37.05390500
N	41.04399000	77.73564100	40.13028100
C	41.25152400	78.18073400	41.50999600
C	40.81808500	77.20138400	42.58684500
O	41.28661100	77.29904700	43.75172400
H	40.58699100	78.40224000	39.49721100
H	42.31267600	78.38131200	41.69551100
N	39.92884300	76.22633200	42.26298100
C	39.56935400	75.18818400	43.23030800
C	40.77330200	74.39078100	43.76456800
O	40.70911200	73.86662900	44.89657800
H	39.55950900	76.19977500	41.32425500
H	39.08944600	75.62374900	44.11010700
N	41.84696600	74.28956400	42.92799900
C	43.15807200	73.81031800	43.38971700
C	44.13409100	74.98298500	43.57878300
O	44.88483000	75.05119300	44.58667200
C	43.70571200	72.71555200	42.43680000
C	45.13401000	72.19422200	42.76966100
C	45.22794900	70.67687800	42.50923500
C	46.22302500	72.93842300	41.96936700
H	41.79591400	74.71038900	42.00497300
H	43.00407300	73.38815000	44.38484100
H	42.98218600	71.89129300	42.48406700
H	43.68306300	73.09489500	41.40432400
H	45.32127000	72.36614600	43.83960800
H	47.23136400	72.61536400	42.24763000
H	46.17900400	74.02062900	42.12820000
H	46.09267200	72.75440100	40.89326300
H	44.51250000	70.11976000	43.12712200
H	45.01497800	70.44853600	41.45572000
H	46.23477900	70.30710800	42.73357600
N	44.16705200	75.92431100	42.59148800
C	45.18744000	76.96087500	42.57337100
C	45.15871500	77.94078700	43.74044800
O	46.20754400	78.60290600	44.00779700
H	43.54410900	75.86461200	41.78804000
H	46.18845400	76.51581800	42.58716200
H	45.09140400	77.51911700	41.63640100
N	44.03187500	78.06027500	44.46543100
C	43.98041700	78.90409300	45.66785800
C	45.03071800	78.51721100	46.72375000
O	45.38215900	79.38168700	47.56944900
C	42.56782300	78.85293700	46.27589300
H	43.16967200	77.58996600	44.18322700
H	44.22694900	79.93519100	45.39341700

H	42.30316000	77.82851300	46.55785800
H	42.53074300	79.48809300	47.16295400
H	41.82896800	79.20568500	45.55172700
N	45.49847300	77.23995800	46.72525800
C	46.51778300	76.79563400	47.67321300
C	47.90250400	76.52815700	47.06555300
O	48.72529200	75.82248300	47.69477700
H	45.17763300	76.56864500	46.02829700
H	46.61563400	77.57235100	48.43784100
N	48.15437600	77.11117500	45.86136700
C	49.38994200	76.85358700	45.10734300
C	49.97040900	78.11449500	44.45087900
O	51.20474800	78.34545100	44.49972800
C	49.12863100	75.76323300	44.03123900
C	50.32640300	75.47877500	43.09765100
C	49.91130100	74.50821400	42.00077600
O	49.66892200	73.29591800	42.24236700
N	49.77040300	75.05590900	40.76377700
H	47.41343100	77.64471900	45.41844800
H	50.13414400	76.50167800	45.82385700
H	48.83182400	74.83933300	44.53867800
H	48.27104100	76.08626400	43.42513000
H	51.13970400	75.02334800	43.67327200
H	50.70526000	76.40650600	42.65957300
H	49.94261400	76.04796300	40.55934700
H	49.46509100	74.44767300	40.01776700
N	49.10859000	78.87481700	43.72050600
C	49.57342200	79.97526700	42.85843100
C	49.86600900	81.24392900	43.67990600
C	48.51495700	80.23071700	41.76612600
C	48.27310700	79.03946500	40.80783800
C	49.46297200	78.79472100	39.87152300
O	49.67645500	79.69278600	38.96059600
O	50.19344000	77.76172000	40.04853400
H	48.10574600	78.70710800	43.75066000
H	50.50950200	79.63859200	42.39624600
H	47.56801000	80.50030400	42.25638800
H	48.83459000	81.09735200	41.17402400
H	48.07142200	78.12916600	41.37817200
H	47.39288200	79.26122500	40.18804000
C	47.88375300	80.03803000	36.06361300
C	47.82699100	81.47806800	36.55563400
O	47.30611300	82.42213300	35.87459000
C	47.68626600	79.84449800	34.54655000
C	47.36820700	78.35821900	34.24978300
C	48.91062000	80.34199200	33.75451700
C	47.01290900	78.06445600	32.78308500
H	48.81970000	79.58050800	36.40931400
H	46.81521000	80.44034000	34.24042900
H	48.22567900	77.73823500	34.55391500
H	46.52551600	78.04904000	34.88678100
H	49.16177100	81.36932800	34.03391800
H	48.72677200	80.31889100	32.67476100
H	49.78516500	79.71000600	33.96309800
H	46.72244300	77.01486700	32.65351800
H	46.17407200	78.68925800	32.45090700
H	47.85821400	78.25660200	32.11276800
N	48.29307300	81.68583900	37.80465000
C	47.98769100	82.90533500	38.54033900
C	48.89003600	84.11654200	38.31965200
O	48.59189000	85.20625400	38.88159000
H	48.78367500	80.90954100	38.31001300
H	46.97099600	83.23186700	38.29694200
N	49.97864900	83.95556900	37.53239600
C	50.82314300	85.08623000	37.15839500
C	50.48865600	85.68742200	35.78663100
O	50.50676100	86.92602200	35.60663300
H	50.19430200	83.04883700	37.12484100
H	50.69120800	85.87982500	37.89379300
N	50.21639200	84.78676600	34.78897700
C	49.93636800	85.23086800	33.42535100
C	48.43978400	85.31113800	33.07878200
O	48.08996500	85.86142600	31.99876500
C	50.67201100	84.35077100	32.39761300
H	50.40972000	83.79979700	34.95662500
H	50.29745800	86.26229900	33.34962300
H	50.45182400	84.69813600	31.38536200
H	51.75276600	84.40295500	32.56389900
H	50.35716100	83.30313300	32.48120000
N	47.54824600	84.77452100	33.95020000
C	46.10480500	84.89460000	33.72841100
C	45.41603000	83.63678700	33.15814100
O	45.23067400	82.60900000	34.17655400
C	46.11927000	83.06236600	31.92644200
H	47.87369900	84.30701100	34.78983900
H	45.96515400	85.72278000	33.02746600
H	44.39574800	83.93575900	32.88544500
H	46.05563900	82.47705100	34.72079400
H	45.54784900	82.21459000	31.53547100
H	46.22661400	83.82213000	31.14496300
H	47.12147100	82.70893100	32.19109200
H	40.84246300	87.66954500	41.69593200
C	41.50870900	87.79335000	42.55183400
S	41.05668800	86.56108900	43.91639300
H	41.38932600	88.79591300	42.96888900
H	42.54471700	87.64726100	42.24070900
C	52.04982900	81.26386000	35.39398900
O	51.24285900	82.19153300	35.69081700
N	52.66042600	80.46222700	36.27440100
C	52.45668500	80.51613100	37.73737900
C	53.63390200	79.83696800	38.46718800
C	53.88317200	78.40783100	38.01241200
C	52.98714000	77.37756000	38.36097700
C	55.00093400	78.09632100	37.21650100
C	53.22211900	76.06867100	37.92688700
C	55.22846700	76.78450200	36.77775600
C	54.33860600	75.76652700	37.13478200
H	51.50653600	80.03638200	38.00936700
H	54.54677900	80.43135500	38.32918000
H	53.39634600	79.85117000	39.53720700
H	52.10811900	77.59247500	38.96865200
H	55.71026600	78.88086500	36.96044800
H	52.53133100	75.28353900	38.21900700
H	56.10112100	76.56079300	36.17062000
H	54.51524400	74.74681300	36.80542300
H	53.23527400	79.70319200	35.92584600
C	42.32123300	85.38003500	39.82307400
C	44.61342700	83.94236900	43.85225800
C	40.34436700	82.90770500	45.91143500
C	38.06066800	84.17428600	41.81723800
C	43.30733100	85.11303000	40.75608400
C	44.72718400	85.31998300	40.52441900
C	45.37955800	84.90195700	41.66389900
C	44.35487300	84.44519200	42.59111400
C	46.85106100	84.91652800	41.95286900
C	45.30263000	85.88588700	39.26142300
H	45.03205200	86.94365300	39.13824500
C	43.65266100	83.52444900	44.76034500
C	43.94361800	83.05522900	46.10302900
C	42.72156400	82.80694100	46.70531700
C	41.69886800	83.09551300	45.70856200
C	45.30404900	82.91992900	46.71412400
C	42.43646400	82.41353400	48.08324600
C	43.20762400	81.64180700	48.87338500
C	39.35222700	83.12657200	44.96944800
C	37.94168800	82.92831200	45.20720500
C	37.28368600	83.28731800	44.03936400
C	38.30839300	83.70182700	43.09560700
C	37.34915200	82.40166300	46.47833100
C	35.85933100	83.24539800	43.72792200
C	34.83247900	83.33983300	44.59437100
C	39.02565000	84.60889800	40.92728800
C	38.74125300	85.07565400	39.58124800
C	39.94778600	85.41529800	39.01366900
C	40.96835900	85.15836300	40.01470600
C	37.36914900	85.15260500	38.98100800
C	40.22562400	85.94445300	37.63876300
H	40.71965800	86.92389800	37.67442700
N	43.08623900	84.57657700	42.02317300
N	42.27956100	83.53550600	44.52640100

N	39.57219400	83.58349900	43.66827700
N	40.39434100	84.66197800	41.18053200
Fe	41.31777000	84.32489200	42.93891800
H	44.92499900	85.34832100	38.38209700
H	46.39334000	85.81637200	39.24015500
H	41.51000300	82.81005700	48.49918400
H	35.61411200	83.12320700	42.67344500
H	39.30404500	86.06523900	37.06236300
H	40.88248100	85.27226500	37.07184900
H	44.11094500	81.14959100	48.52649600
H	42.91095500	81.44194700	49.89927400
H	34.97209600	83.50780900	45.65619700
H	33.80553700	83.27739600	44.24878100
H	37.40389700	85.53105200	37.95554400
H	36.71351000	85.82084600	39.55446800
H	36.88074500	84.16946700	38.95150100
H	47.43546400	85.11221000	41.04926100
H	47.10508700	85.69297300	42.68749400
H	47.19154800	83.95986500	42.36971600
H	36.43839800	81.82863600	46.27520600
H	38.04466100	81.74326600	47.00772500
H	37.07556300	83.21107600	47.16962700
H	45.65659900	81.88042900	46.70012200
H	45.27729200	83.22143900	47.76788900
H	46.04902200	83.53895200	46.20616300
O	42.34580900	81.14837000	39.74657300
C	42.06274200	81.06817300	38.36660700
C	40.97352000	80.48265600	37.73765300
O	39.98570500	79.77691500	38.45227400
C	38.80225300	80.55886200	38.88936500
C	40.91761100	80.55670900	36.33291700
C	41.93201800	81.19446800	35.61011700
C	43.04195200	81.78538700	36.24779300
C	43.06635800	81.69759500	37.62844100
O	44.04101500	82.22001500	38.50434000
C	43.73116800	81.68889500	39.83129600
H	40.07919000	80.08969300	35.82805000
H	41.88087400	81.22548900	34.52771000
H	43.83557100	82.25419400	35.66885300
H	41.05339800	81.39368800	41.10867200
H	38.46059200	81.19066800	38.06477100
H	38.04269900	79.81551600	39.13459400
H	39.05735000	81.14261900	39.77653900
H	44.40962500	80.86445400	40.06740700
H	43.74950900	82.48833000	40.56430600
H	40.11728700	81.84109500	42.35076400
O	40.29474100	81.14963100	41.68180300
H	51.87069700	84.76381000	37.15825400
H	48.01656900	82.67093400	39.60936300
H	45.62532800	85.14980400	34.67958100
H	37.01310400	76.59174500	45.32907700
H	50.25131200	82.04369100	43.03494100
H	48.95505900	81.60270600	44.17540800
H	50.61576000	81.02211900	44.44421700
H	45.65015800	83.88276800	44.15743000
H	40.03592400	82.54896100	46.88457400
H	37.02959700	84.22325100	41.49062900
H	42.63661000	85.77936700	38.86736200
H	47.08323400	79.50331500	36.59886400
H	40.71504700	77.04043100	37.73670000
H	40.72260900	79.13451100	41.65450700
H	38.85888200	74.50640800	42.75259700
H	52.30588500	81.06352500	34.34603100
H	52.38765400	81.56964300	38.02809900
H	46.20609900	75.86841900	48.16133500

$^{2}Re_{B}$

C	36.19600800	79.30843500	39.18237400
C	36.03522400	78.08856600	38.24999300
C	37.56199400	79.38758800	39.88532300
C	36.95655300	78.11894900	37.01902300
H	36.03062800	80.22723900	38.59838300
H	34.98958700	78.02979300	37.91473500
H	36.22538900	77.17101800	38.82726200
H	37.60496500	80.24258500	40.57019700
H	37.74488900	78.48014200	40.47742200
H	38.39194200	79.50369500	39.18059100
H	36.79553700	77.24097200	36.38205800
H	38.01319500	78.13587600	37.30759300
H	36.76199700	79.01307900	36.41175000
H	49.30095900	81.61220300	27.64340900
C	49.30035200	82.63496400	27.24108100
C	50.24846400	83.53087300	28.01014700
C	49.82870100	84.18839800	29.17969800
C	51.57657400	83.70697400	27.58627900
C	50.70589600	84.99511900	29.91470500
C	52.46089400	84.51126000	28.31466600
C	52.02828400	85.15535300	29.47946500
H	48.26944000	83.00270100	27.29546500
H	49.58018400	82.56841200	26.18375400
H	48.80281300	84.06893000	29.51911100
H	51.91772800	83.21118500	26.68066300
H	50.35791500	85.49226600	30.81619500
H	53.48393200	84.63579200	27.97082100
H	52.71280000	85.78060700	30.04462800
C	40.38570700	76.11005300	39.97828300
C	40.99923100	76.47469300	41.31638500
O	41.68493000	75.64576800	41.99358200
C	41.35279700	75.43644800	38.96402000
C	41.71884300	73.99396500	39.36107300
C	40.71220900	75.46698000	37.56173800
H	39.55084000	75.41858900	40.17190000
H	42.27510100	76.03301600	38.92942000
H	40.49634400	76.49328500	37.24338000
H	39.77128000	74.89988100	37.55079100
H	41.38335600	75.01529600	36.82231300
H	42.18942400	73.96234500	40.34548900
H	40.81991000	73.36203200	39.38998100
H	42.40957700	73.55981200	38.62863700
N	40.72459400	77.72166000	41.78456700
C	41.36507400	78.27144600	42.98394600
C	41.12309100	77.53673700	44.29023700
O	41.89493300	77.73993700	45.26587200
H	40.47479800	78.44681100	41.10297500
H	42.44722100	78.32439600	42.83664200
N	40.08016500	76.67198300	44.37870200
C	39.92196900	75.81177700	45.55305700
C	41.10865000	74.85506000	45.79109200
O	41.35042300	74.45420600	46.95000700
H	39.47391400	76.56159700	43.57921100
H	39.83932900	76.41215000	46.46168800
N	41.81180900	74.49005800	44.68202300
C	43.10869100	73.80151500	44.75501100
C	44.26705700	74.79391800	44.56462900
O	45.30121600	74.71580600	45.27896300
C	43.15063100	72.61906300	43.75206200
C	44.50004500	71.84846100	43.67691800
C	44.23952600	70.35708200	43.37941200
C	45.45946700	72.43930900	42.62236100
H	41.52474200	74.83962700	43.77120800
H	43.21194900	73.42293000	45.77421500
H	42.34837300	71.93700700	44.06289200
H	42.88154300	72.98569400	42.75081000
H	44.98848000	71.91924400	44.66002600
H	46.41087100	71.89938500	42.59261800
H	45.70392000	73.48587500	42.81968800
H	45.01479000	72.37937700	41.61976500
H	43.62090200	69.89423300	44.15871100
H	43.71965400	70.23754500	42.41883300
H	45.18278200	69.80228600	43.31803400
N	44.12596100	75.73846200	43.59297800
C	45.21422500	76.64482000	43.23961600
C	45.72038700	77.51629900	44.38404800
O	46.90634100	77.96025400	44.34776300
H	43.29524400	75.74683200	43.00374000
H	46.08412300	76.08449800	42.88151300
H	44.87018700	77.28885200	42.42215100
N	44.88780000	77.77856900	45.41247900
C	45.33598000	78.51900600	46.59864400
C	46.48763900	77.84484000	47.37317000

O	47.12988000	78.54768100	48.19185300
C	44.14655500	78.76368200	47.54164600
H	43.90680500	77.50697500	45.35887200
H	45.75857400	79.47672500	46.27488100
H	43.71011200	77.81524500	47.87291200
H	44.49458000	79.31551900	48.41802000
H	43.36104300	79.33427000	47.03664700
N	46.73358600	76.52465900	47.14890300
C	47.87372700	75.84328800	47.75676600
C	48.99405400	75.46245900	46.77457000
O	49.86171300	74.62456600	47.11558300
H	46.14932400	75.98169800	46.51249300
H	48.28871100	76.51087800	48.51820900
N	48.96439400	76.10173900	45.57423600
C	49.89072400	75.81577100	44.47176900
C	50.59644900	77.08161500	43.95665400
O	51.83617500	77.09353900	43.75236700
C	49.11107500	75.13222300	43.31641100
C	49.91457100	74.92424200	42.01466700
C	48.99193400	74.41614000	40.91408100
O	48.44601000	73.28231200	40.97681400
N	48.76432300	75.28677600	39.89450100
H	48.19555000	76.73793600	45.39024700
H	50.66769900	75.15805000	44.86519200
H	48.72800700	74.16931100	43.66976000
H	48.23972100	75.76165700	43.09363200
H	50.70087000	74.17859700	42.17991200
H	50.39887200	75.85429400	41.70317200
H	49.17385700	76.22860700	39.83365200
H	48.14976600	74.97977800	39.15413000
N	49.78379100	78.12711000	43.64027200
C	50.28190000	79.34453500	42.97801400
C	50.98864400	80.27313200	43.98260000
C	49.10125500	80.04590800	42.27892800
C	48.45648800	79.23754400	41.13079800
C	49.36336900	79.09683900	39.89955700
O	49.69276500	80.19123100	39.28020000
O	49.74142300	77.92903200	39.54088300
H	48.78896700	78.08290000	43.84258500
H	51.01800700	79.02185100	42.23082800
H	48.33529900	80.27236300	43.03539900
H	49.45233600	81.00449900	41.88221900
H	48.16992700	78.24251100	41.48028700
H	47.54177800	79.75820300	40.81320900
C	46.99664700	82.28196700	38.63192800
C	47.86774900	83.25101700	39.41901400
O	47.43547700	84.38659600	39.78531700
C	46.13565900	82.97042800	37.54860800
C	45.15929900	81.94965600	36.91972900
C	47.02014200	83.65500700	36.48988100
C	44.10957300	82.56815200	35.98209100
H	47.61746900	81.48940200	38.19943900
H	45.54143600	83.74606400	38.05051600
H	45.73661500	81.18510700	36.37753100
H	44.63866100	81.42015800	37.72857700
H	47.67676500	84.39779900	36.95872500
H	46.41165400	84.15974200	35.72983100
H	47.65097400	82.91820900	35.97465000
H	43.40877600	81.80129400	35.63034900
H	43.52863900	83.33973700	36.50303100
H	44.56466500	83.02832200	35.09719000
N	49.11188600	82.81547200	39.72906300
C	49.99037000	83.62799200	40.56584500
C	50.71796700	84.77110800	39.85238700
O	50.78429100	85.90686300	40.37694500
H	49.37080700	81.82232100	39.53320000
H	49.42362600	84.09944800	41.37138100
N	51.34344500	84.46197500	38.67076300
C	52.22889200	85.43105300	38.03809900
C	51.99875700	85.67963100	36.55554500
O	52.82579800	86.34980500	35.89645500
H	51.43090600	83.49644000	38.35613600
H	52.10894100	86.37663200	38.57736600
N	50.85363800	85.16173400	35.99527600
C	50.68890300	85.14131200	34.53711200
C	49.67230100	86.16494100	34.01599700
O	49.48883300	86.31116600	32.78097900
C	50.35235100	83.72712600	34.03137700
H	50.34341000	84.48623600	36.55206500
H	51.65390600	85.45743200	34.12278300
H	50.30683900	83.72216300	32.93979800
H	51.11630000	83.02123800	34.36666800
H	49.38480500	83.38619700	34.41722100
N	48.99338700	86.89443900	34.94717200
C	48.12628000	88.01816000	34.57882000
C	46.63629400	87.65353200	34.44391800
O	46.11364100	87.16372900	35.72423800
C	46.32499500	86.66630100	33.31923100
H	49.26901100	86.76963000	35.91275200
H	48.48551800	88.42224200	33.62648600
H	46.08477400	88.58594100	34.28142200
H	46.54787000	86.31396900	35.94693600
H	45.24710600	86.47924300	33.27928500
H	46.66086500	87.05396400	32.35265600
H	46.84149200	85.71421300	33.47902500
H	40.07549100	87.31580900	40.81774400
C	40.66773800	87.38969500	41.73458000
S	40.37115600	85.85375900	42.77892700
H	40.33319300	88.25339100	42.31310700
H	41.72376700	87.47723500	41.47852300
C	53.21873000	81.63380200	36.60650300
O	52.34008100	82.40603300	37.06366600
N	53.35743800	80.32468800	36.89712700
C	52.44531000	79.54311800	37.75197400
C	51.82413500	78.34078500	37.01048500
C	51.02620600	78.72892900	35.78100100
C	49.84844600	79.49052800	35.91449900
C	51.43437900	78.34640900	34.49269000
C	49.10705300	79.85255400	34.78549000
C	50.69297800	78.71053900	33.36101200
C	49.52538500	79.46697500	33.50403300
H	51.65281100	80.20091700	38.11457700
H	52.61223100	77.62647300	36.72990400
H	51.17025200	77.85287900	37.74639400
H	49.53298700	79.79162100	36.91010300
H	52.33657500	77.75009400	34.37575200
H	48.19606500	80.43377500	34.90434200
H	51.02682800	78.40414100	32.37350900
H	48.94755600	79.75185900	32.62948500
H	54.10062800	79.82355300	36.42786800
C	40.45232600	85.46415800	38.65085400
C	44.48199100	84.90263100	41.28672000
C	41.98789100	82.29876000	44.53180300
C	37.93541200	82.93279600	41.93578300
C	41.76819400	85.53434400	39.08800700
C	42.82789400	86.25511200	38.39538100
C	43.95718100	86.14098500	39.17265000
C	43.58991800	85.31423000	40.31677600
C	45.30738400	86.75669200	38.97035500
C	42.67153800	86.99053800	37.09699800
H	42.20760200	87.97750000	37.23670400
C	44.16190400	84.11667500	42.38824100
C	45.11912500	83.70225000	43.39639700
C	44.41000100	82.96716000	44.33405600
C	43.02456100	82.93573800	43.86880600
C	46.58588200	84.00332000	43.36523700
C	44.88031400	82.28463300	45.53357200
C	46.01247100	82.52368200	46.22403600
C	40.66742200	82.24290200	44.11331400
C	39.61033200	81.55768900	44.83259100
C	38.45127800	81.74062800	44.09665000
C	38.82205600	82.52792200	42.92619100
C	39.80152500	80.77316000	46.09561300
C	37.10094900	81.24719200	44.35260900
C	36.53619100	81.03071200	45.55492300
C	38.26082600	83.69523400	40.83317400
C	37.32194700	84.09179000	39.79615100
C	38.03533300	84.80329100	38.86161100
C	39.41323300	84.84770200	39.32599300
C	35.86347900	83.74579000	39.80069400

C	37.55366700	85.43076000	37.58842900
H	37.73892600	86.51279500	37.57299100
N	42.24275700	84.97716100	40.26271100
N	42.89594800	83.63952400	42.68515600
N	40.16871200	82.82944100	42.95741500
N	39.53357500	84.18296700	40.53971500
Fe	41.24985600	83.77237900	41.52571300
H	42.04268500	86.43429700	36.39096200
H	43.64255200	87.14582600	36.61655400
H	44.24001100	81.48322800	45.89846600
H	36.49973700	81.03683800	43.46807300
H	36.47901600	85.28143100	37.45139000
H	38.05821900	85.00462000	36.71121500
H	46.70515500	83.31585900	45.96569000
H	46.27314400	81.91977300	47.08745100
H	37.04220800	81.25551100	46.48748800
H	35.52663400	80.64025600	45.63589400
H	35.34934700	84.17297100	38.93507600
H	35.36018000	84.12331400	40.70025800
H	35.70550900	82.65947300	39.77217600
H	45.40891300	87.18507600	37.96981600
H	45.47753500	87.56813600	39.69268800
H	46.10944200	86.02416500	39.11962800
H	38.98941600	80.05211900	46.22833300
H	40.74072100	80.20916100	46.08769000
H	39.81151900	81.41856100	46.98486600
H	47.16120900	83.14800900	43.73799100
H	46.84020900	84.86174200	44.00257600
H	46.93814700	84.22979500	42.35509300
O	41.19820200	79.85784900	40.08266300
C	42.17802700	79.39987000	39.20598200
C	41.89193400	79.27288300	37.82920100
O	40.62676300	79.69694200	37.42999200
C	40.35457900	79.79827300	36.00604800
C	42.84293700	78.73031000	36.95533200
C	44.07962900	78.29621400	37.45090000
C	44.37736300	78.40973300	38.80891600
C	43.42783200	78.96210000	39.67778100
O	43.72961600	79.03422800	41.04882000
C	44.22905900	80.34543800	41.51615800
H	42.62914600	78.63545200	35.89819700
H	44.81113600	77.87461900	36.77028900
H	45.32804400	78.07950200	39.21088100
H	41.23490100	80.85576200	40.26275500
H	40.36396700	78.81351200	35.52426900
H	39.35592500	80.22751400	35.93955600
H	41.07913800	80.45677500	35.51342600
H	44.38736900	80.23244200	42.58799400
H	45.17916700	80.57582900	41.02453900
H	43.49281100	81.12760600	41.31668900
O	41.54477600	82.37195400	40.67891800
H	53.27774700	85.12785600	38.13446100
H	50.74120600	82.95901400	41.00247400
H	48.22718900	88.78660000	35.35121300
H	35.39960600	79.27769000	39.94069500
H	51.39722900	81.15517900	43.47285100
H	50.28870600	80.61127200	44.75650300
H	51.81175800	79.73789100	44.46389300
H	45.51668700	85.19136200	41.15526400
H	42.22360000	81.80371000	45.46501900
H	36.89869200	82.63976900	42.04471800
H	40.21855000	85.94145300	37.70668200
H	46.32118800	81.79583000	39.35136300
H	39.95315600	77.01019600	39.52772000
H	41.01277400	79.30178500	43.09318100
H	39.00045700	75.23371300	45.43490300
H	53.97625000	82.00528500	35.90024600
H	52.99766600	79.18201100	38.62824400
H	47.55439100	74.91907200	48.24638000

^{2}TS1$_{HA,B}$

C	36.36314800	80.24277500	43.65744300
C	35.36182600	79.22669400	43.06916600
C	37.77003000	80.16524800	43.03814200
C	35.01062400	79.48260900	41.59448400
H	35.96612700	81.25948900	43.51047500
H	34.43874500	79.24444700	43.66641600
H	35.77681600	78.21302000	43.17716300
H	38.47904000	80.78360100	43.59999500
H	38.13497200	79.12604500	43.04278000
H	37.79109200	80.51890700	42.00215300
H	34.31015500	78.72877200	41.21534700
H	35.90272200	79.46319000	40.95846900
H	34.53687600	80.46651000	41.47608100
H	47.59139000	80.58277800	26.58034500
C	48.06484300	81.36977200	25.98124700
C	49.09807300	82.13072200	26.78530600
C	48.79229500	82.61592500	28.06970500
C	50.37930800	82.37938000	26.26696800
C	49.73339900	83.32962300	28.81973000
C	51.32894600	83.09096500	27.01113300
C	51.00868700	83.56599500	28.28733800
H	47.26323300	82.03192100	25.62646000
H	48.51365500	80.89557600	25.10182300
H	47.80616000	82.43044200	28.48847700
H	50.63483600	82.01013100	25.27678700
H	49.47170400	83.70124200	29.80677400
H	52.31607000	83.26988300	26.59402200
H	51.74365400	84.11702900	28.86612200
C	40.58356300	77.86793400	40.19619600
C	41.45587100	78.22949700	41.37635300
O	42.21845400	77.37081300	41.92870900
C	41.37543900	77.79779500	38.85185800
C	42.36567400	76.61895000	38.82035000
C	40.38041000	77.71346400	37.67794400
H	40.13233600	76.88768700	40.39228800
H	41.94083000	78.73575900	38.75403800
H	39.71530900	78.58426200	37.65166500
H	39.76174200	76.80913200	37.75609100
H	40.91672200	77.66930600	36.72300500
H	43.06574300	76.65163700	39.65912400
H	41.82956700	75.66189600	38.87247800
H	42.94211300	76.63031500	37.88780300
N	41.39940500	79.50886300	41.81549900
C	42.34089700	80.02389000	42.80953800
C	42.24664800	79.37035600	44.18433700
O	43.23559700	79.37835100	44.96464900
H	40.82506400	80.19796300	41.30508900
H	43.37466900	79.87761000	42.47879800
N	41.06890600	78.78090600	44.52331900
C	40.93814400	78.01346000	45.76028600
C	41.80033600	76.73753000	45.80117000
O	42.20612800	76.30235900	46.90290400
H	40.28940800	78.85479400	43.88421400
H	41.24795700	78.61114500	46.62045500
N	42.04525700	76.13589000	44.60343300
C	43.00359300	75.02838200	44.46253800
C	44.42795600	75.56806500	44.26108600
O	45.40969400	75.02634500	44.83383300
C	42.54462100	74.06793800	43.33569800
C	43.54685100	72.92714800	42.99490600
C	42.78807500	71.62981400	42.64931800
C	44.49060800	73.31699700	41.83797200
H	41.74827800	76.58931900	43.74379000
H	43.03317600	74.49701400	45.41625200
H	41.58825400	73.64352400	43.66930000
H	42.33495400	74.65511800	42.43059000
H	44.16263800	72.73281800	43.88485600
H	45.25647900	72.55681000	41.65505500
H	45.01928000	74.25410600	42.03873600
H	43.91563200	73.45174600	40.91065900
H	42.16900200	71.29116300	43.48990800
H	42.12916400	71.78271000	41.78328200
H	43.48968400	70.82700400	42.39605800
N	44.56716200	76.65871900	43.45892500
C	45.87809300	77.23954000	43.19828600
C	46.62619800	77.66903900	44.45980000
O	47.89151200	77.68520600	44.46644100
H	43.76573500	77.01987200	42.94337200
H	46.53175100	76.52745600	42.68437800

H	45.74555000	78.10525100	42.54198800
N	45.88633100	77.99581600	45.53732800
C	46.46493400	78.30825600	46.85000000
C	47.39198800	77.21634200	47.42058800
O	48.20170800	77.55210500	48.31924600
C	45.32235400	78.59058300	47.84314200
H	44.88319300	78.11646900	45.42139200
H	47.11027900	79.18910300	46.75974300
H	44.65537400	77.72452100	47.92342200
H	45.74541400	78.80578600	48.82733300
H	44.72560900	79.44805800	47.51393800
N	47.26192000	75.94203800	46.95985200
C	48.15613000	74.87878600	47.40927700
C	49.05435900	74.28673700	46.31391700
O	49.56622900	73.15370400	46.47886500
H	46.55577800	75.70080300	46.26449900
H	48.78593600	75.29832000	48.20042300
N	49.25102200	75.06326900	45.21229300
C	49.94734300	74.56163500	44.01935200
C	50.87965800	75.59645500	43.37204700
O	52.02562900	75.25973100	42.97832200
C	48.90894200	74.06537300	42.97276200
C	49.52432200	73.60540000	41.63244400
C	48.42893100	73.30895200	40.61864000
O	47.65758700	72.32024100	40.74305500
N	48.31763100	74.20667300	39.60372900
H	48.76218900	75.95163900	45.15887900
H	50.57696100	73.73146300	44.34410100
H	48.33381700	73.24542600	43.41517200
H	48.20243900	74.88502600	42.78532300
H	50.10312100	72.68852600	41.79185300
H	50.20762500	74.36367700	41.24008100
H	48.91638600	75.03457000	39.50162000
H	47.59147000	74.04916500	38.91989500
N	50.34916500	76.82513400	43.12214000
C	51.03129900	77.81345400	42.26614100
C	52.16042900	78.52842700	43.02989400
C	49.98111400	78.80791600	41.72940500
C	48.90797000	78.18139600	40.80387100
C	49.46249900	77.82134300	39.42063800
O	49.60002400	78.79691900	38.56885800
O	49.79310400	76.61006700	39.18313300
H	49.44923200	77.09419200	43.51348300
H	51.47138000	77.25546200	41.43061800
H	49.48363600	79.28284600	42.58717800
H	50.50322100	79.60291900	41.18141100
H	48.49815600	77.28046900	41.26856000
H	48.09294600	78.90441700	40.67307900
C	46.56577800	80.35754900	37.83225900
C	47.27146900	81.49980400	38.54527300
O	46.64312300	82.55892100	38.85011900
C	45.89593500	80.82369100	36.51453100
C	45.02474600	79.68452100	35.93538000
C	46.95233800	81.33370800	35.51546600
C	44.26684300	80.04884200	34.64776700
H	47.26750000	79.53872600	37.63839800
H	45.23386000	81.66517700	36.76811700
H	45.65805400	78.80402000	35.74949400
H	44.29921700	79.38914700	36.70673100
H	47.52893500	82.15918500	35.95094500
H	46.49082500	81.70023100	34.59159400
H	47.65794900	80.53640800	35.25102100
H	43.56673800	79.25288300	34.36771000
H	43.68845200	80.97325900	34.77787500
H	44.94578000	80.20161400	33.80148900
N	48.58529100	81.30312100	38.81889200
C	49.36126500	82.29277300	39.55674100
C	50.08349300	83.34885900	38.71571000
O	49.95081100	84.56876300	38.98134500
H	49.00049100	80.35056400	38.68382400
H	48.70876500	82.85068100	40.22849800
N	50.92188900	82.89506000	37.73231100
C	51.83424000	83.81013700	37.05659800
C	51.61919400	83.99231800	35.56089300
O	52.42348100	84.67274800	34.88476700

H	51.09384700	81.90254900	37.57987500
H	51.73008500	84.79028500	37.53250900
N	50.49919400	83.40985100	35.01941100
C	50.28857200	83.38730700	33.56950000
C	49.22263200	84.38895800	33.10083000
O	48.92066100	84.48348200	31.88444600
C	49.98132900	81.96781000	33.06474900
H	50.01622600	82.73096400	35.59580900
H	51.22820700	83.73757300	33.12364300
H	49.86652800	81.97658600	31.97845000
H	50.79543200	81.29108800	33.33771900
H	49.05518100	81.58086700	33.50435700
N	48.65520900	85.17469600	34.05983700
C	47.77541900	86.29602500	33.72340500
C	46.27452400	85.98709200	33.85179000
O	45.91830900	85.74451200	35.25475700
C	45.79173500	84.84348200	32.96090600
H	49.00564700	85.07408300	35.00330800
H	47.99544000	86.58723100	32.69119300
H	45.73031500	86.90581600	33.60791100
H	46.25888000	84.87149000	35.54017700
H	44.71146100	84.70948900	33.07447000
H	46.02471900	85.04054400	31.91027900
H	46.29002400	83.90526800	33.23060900
H	42.18407500	88.37475500	41.70652000
C	42.89031800	88.21489700	42.52637400
S	42.25839000	86.82754400	43.62949600
H	42.94938700	89.11776000	43.13756000
H	43.86739300	87.95563300	42.11901900
C	53.01928600	80.04499900	35.86920700
O	52.15475800	80.81948500	36.34880400
N	53.21550900	78.75797900	36.21947500
C	52.38075100	78.00767200	37.17541500
C	51.87231100	76.67296100	36.59174000
C	51.07303700	76.81811700	35.31209000
C	49.83923200	77.49733100	35.31547000
C	51.53998500	76.28020900	34.10170400
C	49.10185100	77.62728000	34.13405000
C	50.80284900	76.41265200	32.91793900
C	49.57902600	77.08904100	32.93054000
H	51.53526100	78.63735100	37.46324000
H	52.72191500	75.99629400	36.41725900
H	51.24668300	76.23310000	37.37946200
H	49.47459900	77.91642300	36.24978700
H	52.48516200	75.74199800	34.08810600
H	48.14605700	78.14316800	34.15054200
H	51.18184200	75.98725100	31.99273900
H	49.00152200	77.19344400	32.01644800
H	53.94293000	78.25015000	35.73308800
C	41.48897400	86.61177500	39.57275100
C	45.56052100	84.74587700	41.42230500
C	43.00282400	82.97311500	45.14990300
C	38.92779600	84.81814600	43.28381100
C	42.81308800	86.21698300	39.72433800
C	43.88082900	86.51401400	38.77519300
C	45.04004300	86.00163900	39.31060500
C	44.67259200	85.38422500	40.57807800
C	46.43089100	86.03331400	38.75575900
C	43.70763000	87.24388800	37.47540700
H	43.78227600	88.33337600	37.60420600
C	45.22364400	84.12560600	42.62040300
C	46.16970000	83.45020900	43.48747800
C	45.44020700	82.94304700	44.54672500
C	44.04980900	83.31248000	44.30602800
C	47.63193400	83.29784500	43.21050900
C	45.89066300	82.13810100	45.68058100
C	47.06049300	82.27833900	46.32958700
C	41.66250800	83.28192600	44.96184900
C	40.59065700	82.88492200	45.85504900
C	39.42180400	83.40634300	45.32340600
C	39.80338200	84.11122900	44.10211900
C	40.76214200	82.03194200	47.07470400
C	38.04930000	83.28977100	45.80419900
C	37.64662900	83.10240300	47.07583500
C	39.27887400	85.52595400	42.15217100

C	38.34570400	86.25414500	41.30871500
C	39.07032100	86.76290000	40.25642600
C	40.45118300	86.34785600	40.45452100
C	36.87987300	86.38876600	41.59047400
C	38.59200800	87.58023800	39.09448200
H	39.05085200	88.57791300	39.08143300
N	43.30763600	85.51648800	40.81089700
N	43.94155300	84.04082700	43.13563400
N	41.16363400	84.01915900	43.89836400
N	40.56834300	85.61823100	41.62924100
Fe	42.24309700	84.72701900	42.32764500
H	42.73007400	87.03820900	37.02341200
H	44.46943400	86.93824300	36.75120600
H	45.20837500	81.35072000	45.99858400
H	37.27668700	83.35964900	45.03882500
H	37.50771900	87.72112400	39.12432200
H	38.83423700	87.10146900	38.13670000
H	47.77508600	83.05573100	46.07861300
H	47.33097300	81.62281600	47.15089600
H	38.33769300	83.06322100	47.91053500
H	36.59289800	83.00691200	47.31807200
H	36.35690200	86.87074400	40.75921400
H	36.69343800	86.99295800	42.48869500
H	36.40497200	85.41226600	41.75257100
H	46.43493100	86.39013700	37.72252500
H	47.08372600	86.69741500	39.33838600
H	46.88204300	85.03458300	38.77614000
H	39.85873300	81.44210200	47.26411800
H	41.60126200	81.33666300	46.96516900
H	40.95017800	82.63149900	47.97648900
H	48.04050300	82.44300900	43.75751900
H	48.20761400	84.18386100	43.51071300
H	47.81485400	83.13662200	42.14432800
O	40.12517400	81.66539700	40.57652600
C	39.99712600	81.75276400	39.20474300
C	38.71229400	81.85761400	38.62217300
O	37.64250700	81.88477900	39.51099500
C	36.30824300	82.06447200	38.96969200
C	38.56916400	81.92502500	37.23116100
C	39.69913400	81.88228200	36.40307700
C	40.97296700	81.76366800	36.95647700
C	41.10658300	81.68975100	38.34603800
O	42.39091200	81.46808600	38.89781300
C	43.14922300	82.61185700	39.28675700
H	37.58660000	82.01065200	36.78475600
H	39.57714400	81.93346900	35.32660000
H	41.85982900	81.70251400	36.33761600
H	40.80372300	82.37071900	40.97937800
H	36.03092800	81.23874400	38.30322800
H	35.64957200	82.06750000	39.83677600
H	36.22199000	83.01539700	38.43030700
H	44.18705300	82.33168000	39.45748100
H	43.00570300	83.47223900	38.62919600
H	42.64181400	82.99471900	40.45487800
O	41.91034400	83.20321400	41.39802300
H	52.86696000	83.47291600	37.18417600
H	50.11054200	81.75584600	40.15077500
H	48.01521000	87.13202900	34.38771600
H	36.43248800	80.08654300	44.74295400
H	52.68829600	79.23083100	42.37219700
H	51.75696500	79.08664400	43.88368700
H	52.87752900	77.79046700	43.39926200
H	46.59249100	84.69751600	41.10334900
H	43.25781900	82.40405900	46.03451200
H	37.88157500	84.82742800	43.56313100
H	41.24348300	87.17270800	38.67861200
H	45.78697400	79.97354500	38.50575600
H	39.77604700	78.60200300	40.09588400
H	42.18074100	81.10351600	42.89145300
H	39.88481400	77.74554100	45.88875600
H	53.71646500	80.39627000	35.09371900
H	52.96356000	77.80152500	38.08227300
H	47.58976200	74.04301100	47.83056000

^{2}TS1$_{HP,B}$

C	36.05184500	78.93873200	39.18096300
C	35.97361700	77.70573400	38.25577800
C	37.40517100	79.10774600	39.89285700
C	36.89205700	77.78834000	37.02507100
H	35.83229900	79.84160600	38.58963400
H	34.93467600	77.57415600	37.92024000
H	36.22340600	76.80689400	38.84022800
H	37.38167100	79.95382900	40.58976500
H	37.65166100	78.20985900	40.47618200
H	38.22955700	79.29677300	39.19722900
H	36.80405900	76.88868000	36.40396600
H	37.94323200	77.89910500	37.31297400
H	36.62688800	78.65207900	36.40088100
H	49.70626700	82.09141600	29.61067400
C	49.56705700	82.88761300	28.86651100
C	50.62277800	83.96369800	29.00780900
C	50.55051800	84.90671100	30.04864000
C	51.70701800	84.03385800	28.11681800
C	51.53658700	85.88711200	30.20037100
C	52.69562600	85.01474500	28.26356000
C	52.61371700	85.94406900	29.30688700
H	48.55938500	83.29318900	29.01523100
H	49.60098000	82.42041600	27.87629600
H	49.72333400	84.89117600	30.75157700
H	51.77668000	83.31519500	27.30382700
H	51.44455700	86.59316100	31.01821400
H	53.52592200	85.05199300	27.56374900
H	53.38021100	86.70472000	29.42238000
C	40.56190700	76.07782100	39.75068300
C	41.03835400	76.40624200	41.15084500
O	41.64704300	75.53290400	41.85001200
C	39.97129800	74.65397100	39.62436200
C	38.69279600	74.48993300	40.46826200
C	39.70652500	74.31842200	38.14503600
H	39.83668700	76.82713900	39.41216100
H	40.72637300	73.95855500	40.01374500
H	40.62345000	74.39933700	37.54936200
H	38.96350500	75.00222900	37.71313900
H	39.32323600	73.29668900	38.03926900
H	38.89432000	74.66087100	41.53266500
H	37.91468100	75.19492900	40.14538700
H	38.28784000	73.47576000	40.36967300
N	40.78110900	77.65187200	41.61961100
C	41.43322900	78.17465000	42.82234500
C	41.11837000	77.47209500	44.12852900
O	41.88143300	77.63131400	45.12015900
H	40.56742400	78.40560200	40.94191700
H	42.51946200	78.15029000	42.69784200
N	40.02181800	76.67502000	44.19792200
C	39.78614600	75.83381000	45.37210900
C	40.90818900	74.81154000	45.64413100
O	41.06388400	74.36409500	46.80112500
H	39.43174900	76.59598700	43.38195600
H	39.71480000	76.44343200	46.27577900
N	41.65266200	74.44616100	44.56364300
C	42.89418200	73.66721400	44.67983800
C	44.11972400	74.57162100	44.45867400
O	45.12541400	74.49164100	45.21355700
C	42.84631100	72.44419700	43.72757100
C	44.02252200	71.43746200	43.83857100
C	43.55097800	70.04208400	43.37595900
C	45.27172900	71.86403600	43.03918100
H	41.42572900	74.82793400	43.64790000
H	42.95679000	73.32539300	45.71544200
H	41.90916400	71.92553100	43.96951300
H	42.75252300	72.79426300	42.68891200
H	44.30585800	71.36449700	44.89972000
H	46.04605200	71.09033800	43.09823000
H	45.71201700	72.78802300	43.42090400
H	45.02497200	72.00557000	41.97853100
H	42.70895300	69.68282700	43.98053300
H	43.22675100	70.06934600	42.32660000
H	44.36330700	69.31018400	43.45391600
N	44.06291600	75.44075300	43.41144200
C	45.20215200	76.26459900	43.01938000

C	45.70663700	77.23285200	44.08471200
O	46.89067300	77.67318100	43.99656600
H	43.23983000	75.46832300	42.81001500
H	46.06434100	75.64121200	42.75795800
H	44.91575200	76.83371000	42.12752500
N	44.88507000	77.57107800	45.10017800
C	45.35354800	78.38172600	46.23094500
C	46.50302100	77.73913200	47.03274900
O	47.15892600	78.47759700	47.80740200
C	44.18196000	78.70073300	47.17407000
H	43.90332200	77.29948800	45.08601800
H	45.78326000	79.31123000	45.84197600
H	43.74306300	77.78141700	47.57611100
H	44.55075600	79.30624900	48.00535300
H	43.39164000	79.23978900	46.64423000
N	46.73249800	76.40506400	46.87999900
C	47.86741400	75.75256500	47.52674200
C	49.11078500	75.55385500	46.64176600
O	50.07712300	74.88828400	47.08276000
H	46.12912100	75.83200300	46.28977400
H	48.16940100	76.36366100	48.38211300
N	49.06935000	76.14356900	45.41751100
C	50.16404900	76.07968800	44.44194400
C	50.70610800	77.47587100	44.08570800
O	51.94064600	77.70626100	44.06982900
C	49.68432200	75.35010800	43.15882000
C	50.70732200	75.36534500	42.00278000
C	50.11037300	74.72983000	40.75351100
O	49.79040200	73.51327500	40.71589400
N	49.91836900	75.57854800	39.70816900
H	48.22304100	76.63156000	45.14361900
H	50.98468200	75.53792700	44.91595100
H	49.43708700	74.31346900	43.41172800
H	48.75543400	75.83251800	42.82408900
H	51.59413900	74.78869500	42.29181500
H	51.03377200	76.38625700	41.78581700
H	50.15643200	76.57828800	39.73473700
H	49.51922000	75.19391000	38.86418000
N	49.77820600	78.39313400	43.69291300
C	50.15195100	79.71090100	43.15333000
C	50.46869900	80.70369600	44.28708100
C	49.01675200	80.21831800	42.24275600
C	48.76039900	79.35323600	40.98905800
C	49.89369000	79.42180600	39.95412400
O	50.14589100	80.58156300	39.42318700
O	50.52088000	78.34926100	39.65465700
H	48.78749700	78.18140900	43.76003600
H	51.06308000	79.56050800	42.56130700
H	48.09303800	80.27307200	42.83885000
H	49.25397000	81.24043100	41.92657600
H	48.60041800	78.31019100	41.27296800
H	47.84213400	79.71027300	40.50159500
C	47.24119600	82.25505000	38.57391700
C	47.92257600	83.34779000	39.38747100
O	47.32110700	84.42343900	39.69424600
C	46.32951200	82.79785900	37.45223900
C	45.51354100	81.64692900	36.81933300
C	47.14912500	83.57177700	36.40223800
C	44.42341700	82.10927600	35.83798900
H	47.99371700	81.56491400	38.17580600
H	45.62335700	83.49912200	37.91848100
H	46.20056400	80.95283600	36.31087400
H	45.03763800	81.07031000	37.62389700
H	47.69661100	84.39830000	36.87151200
H	46.50273500	83.98445600	35.61816400
H	47.87995100	82.91171900	35.91604500
H	43.81780100	81.25738000	35.50851500
H	43.75192200	82.83281500	36.31784000
H	44.84607900	82.58345500	34.94439000
N	49.18465700	83.08309900	39.79566600
C	49.88648700	84.00142900	40.68904300
C	50.46616100	85.25659300	40.02986600
O	50.29704600	86.38405900	40.54917200
H	49.58433700	82.12979600	39.63224900
H	49.21753300	84.36734000	41.47090200
N	51.22352700	85.06384400	38.90287000
C	51.96106000	86.17489400	38.31585700
C	51.79411700	86.36407700	36.81568100
O	52.52313600	87.17342700	36.19866400
H	51.50171700	84.12878900	38.60685600
H	51.63490200	87.08622900	38.82784800
N	50.81389700	85.62858800	36.19039300
C	50.77393700	85.54618100	34.72550300
C	49.67628200	86.40775100	34.08885500
O	49.58755300	86.50926600	32.83860000
C	50.66597200	84.08494000	34.25534500
H	50.39927300	84.87727800	36.72900800
H	51.71773600	85.97704000	34.37033000
H	50.72028600	84.03979300	33.16520000
H	51.47984200	83.49533500	34.68450200
H	49.71786400	83.63561600	34.57253000
N	48.81777200	87.04303600	34.93733400
C	47.83943000	88.02741400	34.46494500
C	46.43267800	87.45385500	34.21494500
O	45.87454900	86.91432500	35.46054000
C	46.36319700	86.41411200	33.09684300
H	49.01875700	86.97076300	35.92647300
H	48.22182900	88.46135700	33.53498200
H	45.77163900	88.29542800	33.98106500
H	46.39359000	86.12947500	35.73416900
H	45.33204400	86.06608600	32.97854500
H	46.71377300	86.83369200	32.14900700
H	47.00190400	85.55366800	33.32177900
H	39.81228900	86.91851500	40.59915700
C	40.45958600	87.04918400	41.47067300
S	40.33406100	85.53416200	42.58104700
H	40.11262600	87.90377600	42.05574600
H	41.48897100	87.19784900	41.14443300
C	53.70111100	82.56848000	37.08095400
O	52.66587200	83.19885300	37.41183900
N	54.00722600	81.30340900	37.42976000
C	53.13280400	80.39873100	38.19900400
C	52.80555400	79.10233900	37.42993000
C	52.10584200	79.34128500	36.10617000
C	50.81327500	79.90157300	36.08112800
C	52.71906600	79.01742900	34.88478600
C	50.16127200	80.12551200	34.86453700
C	52.06705000	79.24289500	33.66545100
C	50.78423200	79.79971000	33.65149500
H	52.20860100	80.92601100	38.44291800
H	53.72614800	78.52373500	37.26319500
H	52.15616300	78.52275900	38.10036900
H	50.33638500	80.15692400	37.02365500
H	53.71300400	78.57559500	34.88959000
H	49.16023200	80.54915100	34.86127800
H	52.55959500	78.98398600	32.73227800
H	50.27430500	79.97576100	32.70867700
H	54.86877200	80.91875900	37.06431300
C	40.30778200	85.07134800	38.41547100
C	44.34608900	84.75714900	41.07768200
C	41.97253700	82.07686000	44.34653900
C	37.86236400	82.63494200	41.82349800
C	41.61988300	85.20581200	38.85042200
C	42.64928700	85.95078300	38.14189300
C	43.77478700	85.91982500	38.93415400
C	43.43786100	85.11144500	40.09951100
C	45.09263300	86.59744600	38.72136900
C	42.47247200	86.63266900	36.81730200
H	41.98614500	87.61318000	36.92192200
C	44.06559700	83.95740500	42.18122700
C	45.04829400	83.56897800	43.17438800
C	44.37822900	82.79181500	44.10824700
C	42.98873000	82.72355000	43.66073700
C	46.50052400	83.92892300	43.13877300
C	44.88651600	82.10026900	45.28547500
C	46.04778500	82.31643900	45.93542000
C	40.64318100	82.01348700	43.96003900
C	39.60252900	81.35248000	44.72250400
C	38.42630400	81.51747800	44.00993800
C	38.76901100	82.26713000	42.80898700

C	39.82131200	80.60583400	46.00352700
C	37.08146400	81.03460700	44.31336700
C	36.54163300	80.87631700	45.53564100
C	38.16638600	83.35656900	40.68830000
C	37.21052400	83.70569600	39.65066700
C	37.90588400	84.38166000	38.67722300
C	39.28852900	84.45709400	39.12048800
C	35.75402700	83.35436400	39.69263200
C	37.40212300	84.95441300	37.38699100
H	37.56490800	86.03879800	37.33200900
N	42.10831800	84.70965300	40.04517500
N	42.82277200	83.43160400	42.48651900
N	40.11710800	82.57001700	42.79902300
N	39.43226800	83.83778500	40.35709500
Fe	41.15361000	83.50895700	41.34119500
H	41.85581400	86.03568900	36.13451100
H	43.43860200	86.79330700	36.32864200
H	44.25082600	81.30505200	45.66941900
H	36.46497000	80.78008400	43.45142600
H	36.32983000	84.77870100	37.26448700
H	37.90840200	84.50770900	36.52120100
H	46.74532000	83.09755800	45.65868000
H	46.32983000	81.69856200	46.78229700
H	37.06650200	81.14661200	46.44551000
H	35.53503500	80.48863300	45.65621900
H	35.22774400	83.73059900	38.81094300
H	35.25991500	83.78010200	40.57568000
H	35.60086100	82.26776400	39.72653500
H	45.18173500	86.99070100	37.70544300
H	45.21065800	87.44451500	39.41244700
H	45.93063100	85.91583900	38.90827900
H	38.99992500	79.90710900	46.18656500
H	40.74762000	80.02149300	45.98289300
H	39.87441500	81.28023400	46.86927200
H	47.11639100	83.06975100	43.43035800
H	46.73420800	84.74257500	43.83938500
H	46.82244400	84.24709000	42.14433000
O	41.12911600	79.81827200	40.01371900
C	41.93195000	79.31620200	39.02839200
C	41.49789500	79.28905300	37.67751600
O	40.25210900	79.87122500	37.43750200
C	39.85356600	80.08683700	36.05933100
C	42.27622700	78.69157800	36.68039400
C	43.50740800	78.10597400	37.00954800
C	43.96649600	78.13072700	38.32737000
C	43.18660500	78.73556700	39.31960800
O	43.66083200	78.71402200	40.64746200
C	44.26842300	79.97682300	41.10960600
H	41.93368300	78.66808000	35.65300200
H	44.10559000	77.64028100	36.23385600
H	44.91694400	77.69015600	38.60598700
H	41.26274500	80.99483500	40.25966500
H	39.68270100	79.13922400	35.53425800
H	38.91848400	80.64312400	36.11760500
H	40.60528500	80.67432200	35.51897100
H	44.54656700	79.80864100	42.15017900
H	45.16509200	80.19521300	40.51980900
H	43.55228700	80.79785300	41.03132300
O	41.52593800	82.08366400	40.51668900
H	53.03825300	86.06855400	38.48877900
H	50.70408800	83.43935100	41.15523600
H	47.76434100	88.81596800	35.21989800
H	35.25317300	78.86188500	39.93378000
H	50.78645700	81.67148000	43.87788400
H	49.58648600	80.86404400	44.91930800
H	51.27592300	80.30796700	44.90981600
H	45.36652500	85.09153700	40.94208800
H	42.23060000	81.59163700	45.27874000
H	36.82940400	82.34085500	41.96024500
H	40.05761200	85.50665700	37.45547700
H	46.62326400	81.67236100	39.27381400
H	41.43643400	76.16339800	39.09004500
H	41.15044900	79.22788300	42.90799600
H	38.83661000	75.30776900	45.23531300
H	54.46689900	83.04447900	36.45053000
H	53.62418800	80.14492700	39.14624400
H	47.56927900	74.76700100	47.89438200

^{2}IM2$_{HA,B}$

C	36.02287700	78.75339100	41.70022200
C	35.07823000	77.63750800	41.20697500
C	37.50254100	78.54840800	41.33107200
C	34.99936500	77.50770000	39.67683300
H	35.68068200	79.71836900	41.29608200
H	34.07044500	77.82863100	41.60221000
H	35.40021500	76.67779100	41.63855000
H	38.12908600	79.31152600	41.80673200
H	37.84699100	77.55660400	41.66016900
H	37.67979200	78.62160800	40.25291500
H	34.26836900	76.74586200	39.38126200
H	35.96698700	77.21985300	39.24931300
H	34.69895600	78.45812000	39.21718300
H	51.95215500	86.27458000	27.24037100
C	51.25748100	87.12629100	27.25309000
C	51.14993500	87.73081600	28.63727700
C	50.22118400	87.23627600	29.56961200
C	51.99147100	88.78559400	29.03005300
C	50.13316800	87.77007400	30.86128800
C	51.91104700	89.32533800	30.31903100
C	50.98364700	88.81868800	31.23679400
H	50.28750400	86.75755400	26.90118600
H	51.62613500	87.85646700	26.52397300
H	49.55605400	86.42616800	29.28074300
H	52.71141800	89.18656600	28.32065000
H	49.40895600	87.37023500	31.56615600
H	52.56870500	90.14194300	30.60335900
H	50.91792200	89.23869900	32.23587100
C	41.28224700	76.75681600	39.48646800
C	41.70374500	77.28604200	40.83874800
O	42.30947500	76.54135700	41.67770000
C	42.48422900	76.49670500	38.52360800
C	43.37858600	75.33270300	38.98921200
C	41.95322300	76.23730100	37.09968600
H	40.75803000	75.80570000	39.64819000
H	43.09109100	77.41511500	38.49582000
H	41.38639600	77.09465600	36.72046600
H	41.29866400	75.35523500	37.08629900
H	42.78236600	76.04664900	36.40826800
H	43.75764400	75.49023000	40.00128500
H	42.81313200	74.39118600	38.98826500
H	44.23145300	75.21227200	38.31073600
N	41.41867600	78.57949000	41.12136500
C	41.94767000	79.24088300	42.31922500
C	41.55021900	78.62430800	43.65491100
O	42.28458100	78.78525600	44.66456900
H	41.04401200	79.19217100	40.37830500
H	43.04394600	79.23598000	42.31290000
N	40.39944200	77.90511100	43.71958100
C	40.02317200	77.20980100	44.95076700
C	41.02903600	76.13078700	45.39658800
O	41.12576400	75.84264500	46.60956900
H	39.80796000	77.85272300	42.90244000
H	39.95147600	77.91157200	45.78487300
N	41.74367400	75.52645400	44.40418700
C	42.89557100	74.65413400	44.67783300
C	44.20870400	75.45418100	44.65248000
O	45.11980800	75.23202000	45.49298600
C	42.89896900	73.45604200	43.69234300
C	44.13827800	72.51908400	43.78175800
C	43.72379400	71.05874000	43.50757900
C	45.25943900	72.94441800	42.81039000
H	41.61676300	75.83244000	43.44319600
H	42.79308500	74.29373300	45.70357300
H	41.98346500	72.88862800	43.90539000
H	42.80184900	73.83987000	42.66636200
H	44.53643700	72.57296400	44.80538300
H	46.14919000	72.31447300	42.90665400
H	45.58318200	73.97457100	42.98373300
H	44.90955900	72.87288300	41.77092700

H	42.97833600	70.71086800	44.23388700
H	43.28967000	70.95933100	42.50302200
H	44.59152200	70.39143200	43.56241900
N	44.33088800	76.39398100	43.67573200
C	45.56014800	77.14677000	43.48657500
C	45.98804600	77.99790700	44.67820500
O	47.20515300	78.33498800	44.78891500
H	43.59035300	76.51639200	42.98616400
H	46.40143700	76.47416500	43.28662500
H	45.43731500	77.78819700	42.60815700
N	45.06220800	78.32729300	45.59427500
C	45.41318000	78.99804700	46.85307500
C	46.45770700	78.23940800	47.69814700
O	47.07546700	78.88245200	48.58113500
C	44.13659100	79.23182400	47.67824000
H	44.07555600	78.19475000	45.37511500
H	45.89249900	79.95755900	46.62837600
H	43.64037100	78.28203500	47.90694300
H	44.40031000	79.72605000	48.61632700
H	43.43049900	79.85917700	47.12466900
N	46.63731700	76.90787100	47.46866000
C	47.66924700	76.14757600	48.16901600
C	48.81863000	75.64534900	47.28119900
O	49.53112000	74.68937000	47.66978900
H	46.06248700	76.40914000	46.78900000
H	48.08010900	76.79786000	48.94805700
N	49.00094700	76.30496000	46.10316000
C	49.95850900	75.84517000	45.08709700
C	50.68544100	76.98849400	44.36277700
O	51.91759400	76.90700200	44.12361300
C	49.21850400	74.95552500	44.04580900
C	50.08318700	74.48890700	42.85244000
C	49.20140500	73.83950000	41.79543500
O	48.68030800	72.70644400	41.96598000
N	48.97261000	74.59170700	40.68351900
H	48.34904900	77.04589600	45.86395600
H	50.72225500	75.26164800	45.60393500
H	48.80758500	74.08359100	44.56521800
H	48.36559900	75.53240600	43.66445000
H	50.81473200	73.74922600	43.19524400
H	50.63722600	75.32868100	42.42318000
H	49.36680100	75.52419500	40.52113600
H	48.36627400	74.19996700	39.97739400
N	49.90747300	77.99460000	43.87908100
C	50.42337800	79.02132600	42.95724000
C	51.25337000	80.08081900	43.70419600
C	49.22736700	79.65211600	42.21245400
C	48.43732000	78.67083200	41.30802100
C	49.19227200	78.33854600	40.01923500
O	49.19942600	79.25768200	39.09737000
O	49.81132000	77.22585800	39.92486100
H	48.92702100	78.06840900	44.14345200
H	51.07677000	78.50664600	42.24112400
H	48.54517400	80.08138300	42.95948500
H	49.59845700	80.48132800	41.59585400
H	48.22872400	77.74682800	41.85241300
H	47.47969900	79.13445800	41.03533500
C	47.12601300	80.10073900	36.58151600
C	47.08073800	81.40569300	37.36135400
O	46.41076900	82.42573600	36.96356800
C	47.07265000	80.23631500	35.04550000
C	46.77802200	78.85339800	34.41433700
C	48.37312400	80.85633000	34.50021200
C	46.55761800	78.88178800	32.89308300
H	48.00113800	79.52202400	36.90086400
H	46.23583700	80.90287100	34.79260200
H	47.60333000	78.16675900	34.65601200
H	45.88064100	78.43614300	34.89436200
H	48.59217400	81.80904700	34.99335100
H	48.30965200	81.04128800	33.42226000
H	49.22149900	80.18304100	34.68077400
H	46.27620900	77.88944600	32.52138300
H	45.75402100	79.58006100	32.62576700
H	47.46106400	79.18981700	32.35494500
N	47.71049200	81.39494500	38.55015200
C	47.51991400	82.44467300	39.54187000
C	48.31848400	83.73593600	39.37166700
O	47.93977500	84.76833200	39.98920000
H	48.27967300	80.54941600	38.82729300
H	46.47038300	82.74888300	39.58185000
N	49.43711700	83.69332700	38.60976800
C	50.27200300	84.88039800	38.42682200
C	50.01459400	85.64373300	37.12271500
O	49.96479400	86.89382800	37.10248700
H	49.72119400	82.83736300	38.13919900
H	50.07552900	85.57310300	39.24501100
N	49.89044000	84.87015000	35.99572600
C	49.77750400	85.48165800	34.67249100
C	48.33027900	85.64776700	34.18404400
O	48.10261300	86.23038400	33.09115000
C	50.60830400	84.70552400	33.63542200
H	50.14317300	83.88079000	36.06022100
H	50.16271900	86.50502600	34.75823000
H	50.50767100	85.17389700	32.65391100
H	51.66471800	84.70854600	33.92207500
H	50.26857600	83.66506800	33.55919700
N	47.33495700	85.16231900	34.97483900
C	45.92930600	85.42557800	34.66356300
C	45.16416200	84.26246400	34.00454000
O	44.80085200	83.23413100	34.98253500
C	45.88813900	83.64972800	32.80641000
H	47.58311300	84.70088200	35.84127800
H	45.91783000	86.27993900	33.97977900
H	44.19647800	84.66376600	33.68112100
H	45.57214000	82.88914300	35.51567000
H	45.26271000	82.87984900	32.34416900
H	46.12694900	84.41852800	32.06458000
H	46.82925900	83.18809300	33.12192400
H	42.57745700	87.36054800	41.86516000
C	42.16013400	87.09321100	42.83774500
S	40.68559500	85.93070200	42.62807900
H	41.77934200	87.98877900	43.33524100
H	42.92885300	86.62679300	43.45561600
C	51.65468600	81.42210300	36.01379400
O	50.91268800	82.25626300	36.61196500
N	52.22150800	80.35224100	36.57864100
C	52.03101700	79.95308600	37.98951200
C	53.06998000	78.88408500	38.39015600
C	53.07309300	77.67834200	37.46573300
C	52.03920700	76.72341600	37.53544800
C	54.08795300	77.51483200	36.50485600
C	52.03384500	75.63410800	36.65819100
C	54.07541300	76.42371000	35.62447100
C	53.04563000	75.48094200	35.69968900
H	51.01131300	79.57472000	38.13959100
H	54.07175700	79.33259300	38.41676500
H	52.82532200	78.56973100	39.41077800
H	51.25151800	76.83536500	38.28054500
H	54.90804500	78.22942700	36.46574900
H	51.23921900	74.89746200	36.72906100
H	54.87151900	76.30791900	34.89451700
H	53.03493300	74.62979400	35.02524100
H	52.73440300	79.70537000	35.98944100
C	40.69516900	85.19412000	38.56314400
C	44.70230300	85.06932400	41.31752800
C	42.19258600	82.66491100	44.71383200
C	38.18998700	82.82555000	41.97560000
C	41.98556800	85.40627900	39.03730300
C	43.00708300	86.17007000	38.32698200
C	44.13871100	86.15887000	39.11608900
C	43.80831000	85.37559500	40.29993900
C	45.45183100	86.84584400	38.88184400
C	42.78658200	86.88486600	37.02621500
H	42.08503300	87.72217900	37.14249000
C	44.38234800	84.36132100	42.46952200
C	45.32047100	84.05644500	43.53557100
C	44.60422300	83.38506900	44.51234600
C	43.22961600	83.27315600	44.01865900
C	46.78206100	84.38772600	43.51387500
C	45.05935100	82.82177000	45.77757200

C	46.14578700	83.17437300	46.49222300
C	40.88328100	82.51980500	44.28039700
C	39.83401100	81.87383200	45.05734800
C	38.68512000	81.92672100	44.28966400
C	39.05572500	82.58553900	43.03441500
C	40.03817100	81.23137600	46.39524000
C	37.34723200	81.42174300	44.57711100
C	36.77040700	81.28152600	45.78625100
C	38.53794400	83.45420400	40.79108500
C	37.61328600	83.70731900	39.68976700
C	38.32453500	84.37042600	38.72231200
C	39.68343200	84.52954400	39.23524200
C	36.17081300	83.29926100	39.68298500
C	37.85871900	84.86890400	37.38739400
H	37.97324500	85.95742500	37.29865200
N	42.48678300	84.94333900	40.24204800
N	43.11773700	83.86666300	42.77616400
N	40.38570300	82.94228800	43.05856900
N	39.79180700	83.95101400	40.48819400
Fe	41.42502400	83.97776800	41.66980100
H	42.36983400	86.22397800	36.25511100
H	43.72184100	87.29814400	36.63739200
H	44.44263300	82.01898600	46.17824900
H	36.75574600	81.13870400	43.70640200
H	36.80222500	84.63799700	37.22108800
H	38.42705800	84.41626800	36.56371100
H	46.80627000	83.98300500	46.20103500
H	46.39595400	82.65654700	47.41272500
H	37.25674500	81.58600300	46.70639600
H	35.76681700	80.87856700	45.88239800
H	35.67812900	83.58768900	38.74949800
H	35.61360700	83.76767900	40.50516400
H	36.05543100	82.21242900	39.79285700
H	45.56330300	87.15550100	37.83760100
H	45.53885500	87.75321900	39.49560100
H	46.30622500	86.20899600	39.13580000
H	39.24469000	80.50744700	46.60487900
H	40.99302000	80.69569700	46.43495600
H	40.03359800	81.96466400	47.21375700
H	47.36195800	83.60188500	44.01167500
H	46.99752300	85.32619000	44.04460100
H	47.16504000	84.49821600	42.49453200
O	40.96219100	80.53413700	39.18419500
C	41.05573500	80.57155700	37.88066600
C	40.17900100	79.80760400	37.02205200
O	39.24734900	79.02868000	37.70599800
C	38.25743900	78.30976300	36.92378400
C	40.28595300	79.85944800	35.64179000
C	41.27177300	80.66118600	35.01624000
C	42.14158700	81.42370500	35.77764900
C	42.00501100	81.36476000	37.17317300
O	42.83552900	82.12705600	38.03393000
C	43.66057000	83.07161700	37.72278400
H	39.61576700	79.27911900	35.01903800
H	41.34961900	80.66578900	33.93531000
H	42.92357900	82.01589600	35.30958700
H	41.56453800	81.63719000	40.15952500
H	38.72575600	77.54709500	36.29024500
H	37.60660400	77.83257000	37.65479100
H	37.67385100	78.99793900	36.30124000
H	44.17973700	83.51278900	38.56111200
H	43.85211200	83.35731100	36.68704600
H	42.99097300	82.16208000	40.78576000
O	42.02592000	82.16687500	40.89659400
H	51.32408900	84.57466100	38.45395000
H	47.78238600	82.02597200	40.51986200
H	45.41140200	85.70630500	35.58652500
H	35.93406800	78.82909100	42.79318100
H	51.66345600	80.81826600	43.00223000
H	50.63460700	80.60781000	44.44046800
H	52.08329400	79.59589900	44.22505700
H	45.72506400	85.40405000	41.19842400
H	42.42212100	82.27253400	45.69573600
H	37.15817900	82.51557600	42.08672000
H	40.45284900	85.61026400	37.59186100
H	46.24624500	79.52593100	36.91381000
H	40.58496600	77.45617600	39.01441200
H	41.63671100	80.28985200	42.28727800
H	39.04030900	76.75305100	44.79940400
H	51.88432700	81.54754600	34.94799500
H	52.14539000	80.84669200	38.61275800
H	47.24101400	75.26407700	48.65076400

^{2}IM1$_{HP,B}$

C	36.22212500	78.45856400	40.29343500
C	35.91635000	77.38045300	39.23180500
C	37.11217700	77.96846800	41.44937300
C	37.13920600	76.94037600	38.40846700
H	36.70741000	79.31584800	39.80304500
H	35.14461300	77.76722100	38.55015700
H	35.47197900	76.50465200	39.73041100
H	37.26709200	78.76099800	42.19177900
H	36.62704500	77.12178800	41.96119400
H	38.09712700	77.64547600	41.09449300
H	36.85258300	76.20455300	37.64693400
H	37.91958700	76.48907000	39.03040800
H	37.58507100	77.79898800	37.88964000
H	52.29217100	87.17207900	27.53762400
C	52.26980500	88.26943200	27.58474900
C	52.76142300	88.77532300	28.92483900
C	51.97625400	88.62147400	30.08170500
C	54.01537500	89.39582400	29.04796100
C	52.42528000	89.06822600	31.32965300
C	54.47390200	89.84576700	30.29246700
C	53.68140000	89.68259400	31.43370100
H	51.23434400	88.57740600	27.39495500
H	52.88880000	88.64680700	26.76353500
H	51.00081500	88.14728800	30.00688900
H	54.63468200	89.52710300	28.16385400
H	51.79713400	88.93732800	32.20683200
H	55.44691500	90.32353800	30.36747800
H	54.03515000	90.03170100	32.39913700
C	39.26994900	73.54843900	41.84451900
C	39.93526200	74.50776400	42.80421400
O	41.12906100	74.35265000	43.19663400
C	39.73982000	72.08557500	41.98492300
C	39.31133300	71.47148700	43.33163600
C	39.21793600	71.24856100	40.80213300
H	38.17716300	73.60710100	41.94967900
H	40.83702400	72.09423900	41.94707700
H	39.55153100	71.66830500	39.84593900
H	38.11990800	71.21960800	40.79281300
H	39.57898800	70.21545100	40.86564900
H	39.72846000	72.03153600	44.17696600
H	38.21678500	71.46335600	43.42824900
H	39.66166800	70.43637000	43.41853100
N	39.19007700	75.56655700	43.24278800
C	39.82719400	76.74078200	43.85304100
C	40.49790600	76.46584500	45.19051900
O	41.42164200	77.21437300	45.60386500
H	38.31769200	75.73999500	42.76065000
H	40.57635300	77.16260900	43.17449500
N	40.05304300	75.40711500	45.91874800
C	40.71007000	75.02300900	47.16798100
C	42.16297900	74.54274900	46.99387000
O	42.97711400	74.68668700	47.93344500
H	39.30575400	74.84500700	45.53687900
H	40.75623300	75.87403400	47.85100200
N	42.46224900	73.95292000	45.80293800
C	43.84262800	73.62762400	45.41221300
C	44.50860000	74.83757700	44.73539300
O	45.69997500	75.14897400	45.00195100
C	43.85734600	72.34219600	44.54830500
C	45.25656100	71.91318800	44.02265900
C	45.35505200	70.37450600	43.96981200
C	45.56745900	72.51487400	42.63774600
H	41.76424300	73.93151600	45.06502400
H	44.41233600	73.46416200	46.32960200
H	43.43694400	71.54980000	45.18182400

H	43.16969000	72.47074500	43.70017100
H	46.01456300	72.27706700	44.73209100
H	46.56915700	72.24648700	42.28995200
H	45.52037700	73.60677700	42.63824600
H	44.85164300	72.15027000	41.88880700
H	45.21250400	69.92825200	44.96224400
H	44.59103900	69.95857100	43.29854300
H	46.33487000	70.06113000	43.59180100
N	43.74355600	75.55683000	43.87160700
C	44.25332800	76.74274700	43.18797900
C	44.85160000	77.79163500	44.12783100
O	45.80789200	78.52081900	43.73228600
H	42.81104200	75.23189500	43.62673300
H	45.03542700	76.48441500	42.46875900
H	43.42027900	77.18540700	42.63383900
N	44.32466100	77.87891500	45.36707600
C	44.86248300	78.74981000	46.42006500
C	46.38605400	78.63288600	46.61860700
O	47.01499200	79.64178900	47.02446500
C	44.13646400	78.43606400	47.74257500
H	43.45780400	77.38369500	45.56457700
H	44.70152400	79.80017900	46.15330600
H	44.26777700	77.38442600	48.01958400
H	44.54047400	79.06678500	48.53888200
H	43.06351700	78.63184400	47.64672000
N	46.96052200	77.41480500	46.41904900
C	48.39638500	77.20638600	46.58750700
C	49.17155800	76.93689500	45.29007000
O	50.32567300	76.45157600	45.34430200
H	46.40901100	76.62886100	46.07415700
H	48.80535400	78.10702100	47.05578500
N	48.52960700	77.27596800	44.13916400
C	49.06946900	77.01052600	42.80418700
C	49.31039600	78.28774700	41.98536400
O	50.28889900	78.35135500	41.19218500
C	48.08864000	76.07222300	42.04974200
C	48.50914200	75.68602800	40.61425200
C	47.37024100	74.94617200	39.92619900
O	47.22614700	73.69971200	40.03297800
N	46.48659200	75.72864700	39.24849900
H	47.57484800	77.61433100	44.20422600
H	50.04272400	76.53385600	42.93791300
H	47.95296000	75.16774300	42.65258200
H	47.11815400	76.58221500	42.00880700
H	49.37813700	75.02219800	40.64405800
H	48.78654500	76.58002500	40.04876200
H	46.58537000	76.73915600	39.09077000
H	45.69710200	75.26533000	38.82243400
N	48.37406300	79.26574300	42.08701400
C	48.43839500	80.50568300	41.29117700
C	49.31799500	81.55793400	41.98995900
C	47.01730100	81.02564400	41.01439200
C	46.11220200	80.05047500	40.24129300
C	46.61115800	79.68323100	38.83141500
O	47.08951700	80.62360500	38.09639500
O	46.49634200	78.45599700	38.45335800
H	47.59690600	79.16117200	42.73112300
H	48.90650700	80.24228200	40.33901400
H	46.53471300	81.29104200	41.96560100
H	47.11607800	81.95161400	40.43900900
H	45.96642000	79.12886700	40.81171700
H	45.12447100	80.52566800	40.13073200
C	47.36865200	82.49464200	35.10635700
C	47.52876900	83.58923700	36.14642100
O	47.77753500	84.80391100	35.82447700
C	48.18943500	82.71063600	33.81662900
C	47.58531900	81.88108500	32.65782800
C	49.67788900	82.38662700	34.04964400
C	48.24201900	82.13183700	31.29034100
H	47.58070400	81.52041400	35.56336600
H	48.10247500	83.77462000	33.55276100
H	47.65502300	80.81272600	32.90852500
H	46.51247300	82.11556800	32.58441100
H	50.06989100	82.92810300	34.91615500
H	50.28857900	82.65617100	33.18021700
H	49.81218600	81.31405100	34.24082600
H	47.71896700	81.58257200	30.49779400
H	48.21685800	83.19840400	31.03119700
H	49.28904500	81.80953900	31.27859600
N	47.32521400	83.23310100	37.42996900
C	47.18020100	84.24102800	38.47096700
C	48.45487100	84.95365500	38.91197200
O	48.36421400	86.01669400	39.58981300
H	47.20153600	82.23398100	37.69273800
H	46.49593900	85.02749800	38.13909400
N	49.63830800	84.40376100	38.55276800
C	50.90125700	85.06613600	38.83744800
C	51.41630900	85.99139600	37.73175800
O	52.47235400	86.64170500	37.91476700
H	49.69015900	83.50037200	38.08263700
H	50.79711900	85.67316100	39.74039100
N	50.68562800	86.05907000	36.57586900
C	51.15987700	86.84755100	35.43463900
C	50.09662400	87.80751500	34.88494500
O	50.30279800	88.43854300	33.81457400
C	51.71200200	85.95134200	34.31009500
H	49.89027900	85.43790200	36.44491200
H	51.97779000	87.46323800	35.82850800
H	52.04976300	86.56478200	33.47144200
H	52.55320500	85.36350700	34.68950400
H	50.94042000	85.26229900	33.94913900
N	48.95454100	87.94533200	35.60645900
C	47.83997900	88.78163200	35.16602700
C	46.69125800	87.96788200	34.53583900
O	46.12872100	87.05013700	35.51995300
C	47.09248300	87.22376800	33.26015800
H	48.84580600	87.40158300	36.45123000
H	48.23507300	89.50004100	34.44227000
H	45.87880600	88.67106300	34.31528600
H	46.69618800	86.24041500	35.61088900
H	46.22185000	86.70946000	32.84002300
H	47.49877400	87.91152900	32.51122000
H	47.85826300	86.47407900	33.48297700
H	43.37109200	87.32993400	42.71001500
C	42.61312400	87.01458400	43.43357900
S	41.22306900	86.13664100	42.52108000
H	42.18938100	87.89434800	43.92237300
H	43.06816200	86.34279300	44.16205800
C	51.63471600	81.27073900	37.04476000
O	50.81884300	82.17470700	37.35187300
N	51.45666300	79.95096400	37.24336800
C	50.26835200	79.35040400	37.88002000
C	49.97538100	77.94153300	37.32363900
C	49.58344100	77.89109700	35.85977500
C	48.24694300	78.12390600	35.48102200
C	50.52729600	77.60135300	34.85983200
C	47.87585900	78.06719400	34.13278300
C	50.15670600	77.55071300	33.51031900
C	48.82691100	77.78316500	33.14260200
H	49.41704200	80.01467300	37.70994500
H	50.84807900	77.29587800	37.50233400
H	49.15560300	77.54026200	37.92794500
H	47.51039300	78.33447900	36.25360600
H	51.55810200	77.39485600	35.14030800
H	46.83964400	78.23830100	33.85464300
H	50.90076400	77.32257800	32.75224100
H	48.53316300	77.73770500	32.09784200
H	52.20857400	79.32998200	36.97553700
C	41.07249700	85.35577400	38.55692300
C	45.08649400	85.76215000	41.24508800
C	43.27068900	82.44796500	44.28815800
C	38.96471900	82.77431800	42.07805400
C	42.32750100	85.73952400	38.99912200
C	43.21983200	86.65192300	38.29620400
C	44.31862300	86.84025100	39.10286700
C	44.12851300	85.99435000	40.27372400
C	45.49154600	87.73934100	38.85095700
C	42.97480900	87.26230700	36.94837000
H	42.60978900	88.29623100	37.02927900
C	44.97342300	84.83333700	42.28214100

C	46.05310500	84.46683100	43.18273400
C	45.55617100	83.46670700	44.00390800
C	44.16046500	83.26858300	43.61531300
C	47.43742100	85.04015700	43.14844000
C	46.23507200	82.65467100	45.01080900
C	47.26372200	83.01509000	45.80067200
C	41.91603700	82.29147200	44.00636400
C	40.99204900	81.50659800	44.80319900
C	39.75117800	81.61539500	44.18856400
C	39.94645400	82.46951800	43.01954200
C	41.33504100	80.76734200	46.05935200
C	38.46840200	81.04924200	44.57823200
C	38.23654900	80.01167500	45.41087400
C	39.16653200	83.48214400	40.90784000
C	38.16545900	83.66479600	39.86381900
C	38.77964600	84.35257100	38.84591700
C	40.14848500	84.61260800	39.28047800
C	36.75725800	83.15632100	39.93918100
C	38.21109700	84.79089600	37.53001300
H	38.23400100	85.88335200	37.42090100
N	42.89318400	85.34635600	40.20710800
N	43.83380300	84.09960800	42.55606700
N	41.25891400	82.86717400	42.93209800
N	40.36309700	84.07290800	40.53170100
Fe	42.10348000	84.03169800	41.51832000
H	42.23132500	86.69918700	36.37442500
H	43.90213900	87.27763800	36.36297200
H	45.87878200	81.63031400	45.10871300
H	37.59555000	81.53701200	44.14529500
H	37.17023400	84.47314500	37.41837300
H	38.77384100	84.37050200	36.68623300
H	47.68192700	84.01681100	45.80180800
H	47.69252700	82.29143100	46.48594900
H	39.03098600	79.44588900	45.88349700
H	37.22060700	79.70835400	45.64506900
H	36.17964400	83.45050200	39.05802100
H	36.23117400	83.54694000	40.82026100
H	36.72285200	82.06001300	40.00115700
H	45.31728100	88.74207400	39.26869200
H	46.41638800	87.35249200	39.29116300
H	45.66596000	87.84568200	37.77530000
H	41.46117600	79.69112600	45.87868900
H	42.25907000	81.13979100	46.51099800
H	40.53806900	80.87861300	46.80469700
H	48.16672700	84.30110300	43.49602000
H	47.53223000	85.91687800	43.80515400
H	47.73239700	85.35641900	42.14245800
O	40.09137400	76.19881400	40.41773000
C	41.13983400	76.52577100	39.74987500
C	41.59277200	75.74515900	38.60519800
O	40.81722300	74.63712900	38.34526800
C	41.18328300	73.77852700	37.22791800
C	42.71352500	76.11577600	37.87561500
C	43.43674300	77.26746700	38.24849600
C	43.04921600	78.05583100	39.34187900
C	41.93536000	77.69872600	40.09253700
O	41.50727000	78.36371400	41.20757600
C	42.13373300	79.65272900	41.56704600
H	43.04892500	75.53382400	37.02621000
H	44.33695800	77.55188300	37.71304500
H	43.64335300	78.92056800	39.59820000
H	43.56411000	82.73931500	40.20724100
H	42.18151200	73.35175500	37.37326700
H	40.43568000	72.98684200	37.22261900
H	41.14991000	74.33156200	36.28304200
H	41.58645600	79.98161900	42.44576400
H	43.18997500	79.50603400	41.81269100
H	42.03573000	80.39473400	40.77128400
O	42.67359300	82.56367100	40.57980500
H	51.66723400	84.30668800	39.01175700
H	46.73311800	83.76436800	39.34768700
H	47.44844700	89.32584200	36.03175500
H	35.27278900	78.83281700	40.70383200
H	49.39813800	82.46144500	41.37348400
H	48.89483600	81.83283600	42.96347400
H	50.32451100	81.15839700	42.14769800
H	46.03160000	86.28037500	41.13939600
H	43.66286400	81.90631700	45.13982700
H	37.97200300	82.37209100	42.24134800
H	40.75814000	85.72140500	37.58674700
H	46.29673500	82.48439600	34.85004800
H	39.51240200	73.93023200	40.84200500
H	39.05890500	77.50798400	43.99853600
H	40.11898200	74.23254500	47.64038800
H	52.59361300	81.51670300	36.56453100
H	50.43473300	79.26697500	38.96136000
H	48.58721400	76.35848900	47.25174800

$^2Pr_{rc,B}$

C	36.70011000	77.72541800	45.36896700
C	35.20204800	77.81521500	45.00785500
C	37.62071400	78.55933000	44.46164700
C	34.57958000	79.20331300	45.23677300
H	36.83689500	78.03538400	46.41574000
H	34.64911000	77.07426800	45.60306900
H	35.06967400	77.52415000	43.95442600
H	38.67478800	78.40442300	44.71829000
H	37.48946700	78.27793800	43.40817000
H	37.41425200	79.63241100	44.54387000
H	33.50801000	79.19918300	45.00246600
H	35.05030800	79.97191500	44.61358900
H	34.68936800	79.51058200	46.28513500
H	50.15654200	84.53455600	25.79361000
C	49.62798700	85.49603300	25.73037000
C	49.93457400	86.37493800	26.92490700
C	49.29489100	86.15957700	28.15871200
C	50.87816600	87.41178900	26.83520000
C	49.58738500	86.94931400	29.27707800
C	51.17781200	88.20694500	27.94820900
C	50.53482400	87.97704400	29.16954600
H	48.55725900	85.27041900	25.66314600
H	49.93145600	85.97456300	24.79273900
H	48.55685200	85.36615600	28.24679800
H	51.37873700	87.59717100	25.88783900
H	49.08036200	86.75869800	30.21956800
H	51.90929400	89.00535800	27.85879100
H	50.76452500	88.59425100	30.03286100
C	41.50894300	76.40536600	38.14784200
C	41.66678200	76.63727300	39.63531000
O	42.36909200	75.86267100	40.35983500
C	42.80099300	76.72423000	37.33033000
C	43.92380800	75.70335600	37.59127900
C	42.45488100	76.79124100	35.83009500
H	41.24871700	75.35082900	37.99196700
H	43.15192400	77.72049300	37.63988900
H	41.71999600	77.57749800	35.62343000
H	42.04444700	75.83361400	35.48299600
H	43.35160000	77.00562400	35.23768800
H	44.15490700	75.61694800	38.65579200
H	43.62785200	74.70843200	37.23262300
H	44.83603200	75.99430100	37.05755600
N	41.02597900	77.71510100	40.15720300
C	41.24172200	78.16249000	41.53484400
C	40.82117200	77.18201900	42.61532100
O	41.29942900	77.28122400	43.77600300
H	40.56321300	78.37993900	39.52658600
H	42.30298600	78.36960100	41.71210600
N	39.93108800	76.20497400	42.29946100
C	39.57948000	75.16886000	43.27163900
C	40.78792700	74.37410600	43.79934600
O	40.73091900	73.84967200	44.93162300
H	39.55402800	76.17700200	41.36386400
H	39.10525100	75.60618300	44.15370900
N	41.85703600	74.27478900	42.95675300
C	43.17163000	73.79875700	43.41168900
C	44.14728300	74.97320500	43.59143700
O	44.90314500	75.04594300	44.59511400
C	43.71491900	72.70191000	42.45870600
C	45.14537700	72.18253900	42.78535100
C	45.23884400	70.66440300	42.52943100

C	46.22961300	72.92474600	41.97678000
H	41.79995500	74.69553100	42.03403000
H	43.02420800	73.37921500	44.40891800
H	42.99232400	71.87720700	42.51219600
H	43.68637700	73.07830300	41.42528600
H	45.33842300	72.35809500	43.85368000
H	47.23959900	72.60313200	42.25074200
H	46.18580800	74.00740400	42.13250600
H	46.09350300	72.73725800	40.90197200
H	44.52708400	70.10890900	43.15302400
H	45.02029500	70.43244900	41.47783700
H	46.24711100	70.29599300	42.74953600
N	44.17494900	75.91068500	42.60022700
C	45.19623200	76.94623900	42.57254600
C	45.17509700	77.93097000	43.73589000
O	46.22706300	78.59069600	43.99647300
H	43.54675500	75.84907400	41.80109200
H	46.19687700	76.50030400	42.58207400
H	45.09499300	77.50116100	41.63412100
N	44.05058000	78.05825100	44.46319600
C	44.00311000	78.91181800	45.65915200
C	45.05395600	78.53139900	46.71681000
O	45.40950700	79.40165200	47.55433500
C	42.59132600	78.86800800	46.26954300
H	43.18734600	77.58545200	44.18843000
H	44.25135100	79.94020700	45.37618600
H	42.32441600	77.84592900	46.55779200
H	42.55714500	79.50873600	47.15258300
H	41.85233800	79.21852600	45.54442200
N	45.51691100	77.25198100	46.72882100
C	46.53472500	76.81159100	47.67994400
C	47.91875200	76.53579400	47.07452300
O	48.74137900	75.83626800	47.71086900
H	45.19315800	76.57647900	46.03753700
H	46.63500900	77.59373300	48.43873000
N	48.16969500	77.10515400	45.86372600
C	49.40466800	76.84114900	45.11130800
C	49.99049500	78.09920000	44.45418000
O	51.22546700	78.32654000	44.50448400
C	49.13965600	75.75105200	44.03588700
C	50.33680700	75.46186200	43.10282100
C	49.91871700	74.49251500	42.00604700
O	49.67335700	73.28082400	42.24791300
N	49.77840600	75.04039100	40.76911000
H	47.42847800	77.63446100	45.41621100
H	50.14655900	76.48714500	45.82918400
H	48.83923600	74.82850800	44.54366700
H	48.28336200	76.07681100	43.42940300
H	51.14814400	75.00353800	43.67890100
H	50.71938900	76.38808000	42.66474600
H	49.95529500	76.03137900	40.56314300
H	49.47126500	74.43263400	40.02346000
N	49.13169800	78.86123300	43.72218300
C	49.60018300	79.96044800	42.86069600
C	49.89347700	81.22883100	43.68238100
C	48.54401800	80.21733500	41.76662600
C	48.30209300	79.02648800	40.80808300
C	49.49233700	78.78023900	39.87265100
O	49.71189900	79.68191600	38.96652300
O	50.21635500	77.74184700	40.04422300
H	48.12851200	78.69542400	43.75031600
H	50.53656000	79.62197900	42.40038100
H	47.59673800	80.48859100	42.25514000
H	48.86615800	81.08339800	41.17522900
H	48.09832300	78.11635500	41.37788900
H	47.42286300	79.24940200	40.18724700
C	47.88451000	80.05290500	36.09396100
C	47.84439200	81.49099700	36.59299600
O	47.31892000	82.44094300	35.92357300
C	47.67719300	79.86918200	34.57697400
C	47.34669700	78.38697200	34.27367600
C	48.90077000	80.36240200	33.78109700
C	46.97965300	78.10446800	32.80765300
H	48.81734500	79.58400000	36.43261800
H	46.80876200	80.47311000	34.27938200
H	48.20175400	77.75912500	34.56837600
H	46.50606700	78.07996800	34.91439700
H	49.16020700	81.38672300	34.06404400
H	48.71131700	80.34575900	32.70217400
H	49.77225900	79.72382100	33.98223400
H	46.68125900	77.05765700	32.67366800
H	46.14286800	78.73693200	32.48493000
H	47.82177000	78.29508300	32.13289000
N	48.32989800	81.69112800	37.83573400
C	48.03965600	82.90906300	38.58009200
C	48.93716400	84.12088300	38.34440300
O	48.64083800	85.21334700	38.90179900
H	48.82180400	80.90941800	38.33150500
H	47.01872900	83.23659700	38.35728900
N	50.02121500	83.95725800	37.55116600
C	50.86251900	85.08736500	37.16851500
C	50.51253300	85.69096100	35.80173200
O	50.53123900	86.92968400	35.62268300
H	50.23387200	83.04985900	37.14348300
H	50.74047200	85.88040400	37.90623900
N	50.22586400	84.79205600	34.80649800
C	49.92855800	85.23891900	33.44747700
C	48.42752100	85.32908600	33.12310400
O	48.06535000	85.88570600	32.05037300
C	50.64317000	84.35494200	32.40832400
H	50.41966000	83.80462800	34.97042200
H	50.29492400	86.26810500	33.36692800
H	50.41057100	84.70507900	31.39979800
H	51.72658000	84.39970900	32.55881300
H	50.32256700	83.30934300	32.49535500
N	47.54584800	84.79327600	34.00486300
C	46.09997600	84.92096400	33.80427400
C	45.39730400	83.66882100	33.23817900
O	45.21929600	82.63821700	34.25503600
C	46.08196500	83.09558600	31.99553200
H	47.88069800	84.32056800	34.83793400
H	45.95412500	85.75298100	33.10915600
H	44.37531200	83.97430700	32.97931500
H	46.04862600	82.50350800	34.79200200
H	45.50072800	82.25296700	31.60787200
H	46.18431100	83.85819000	31.21615600
H	47.08512400	82.73496300	32.24639400
H	40.87915800	87.48495500	41.55746000
C	41.54652800	87.59260300	42.41311800
S	41.09264800	86.35545000	43.76679800
H	41.42071200	88.58365400	42.85751200
H	42.58331500	87.45753000	42.10397600
C	52.06301900	81.26286700	35.38961200
O	51.26358800	82.19406500	35.69550300
N	52.67588300	80.45479300	36.26254600
C	52.48302800	80.50359800	37.72718800
C	53.65987100	79.81158800	38.44562800
C	53.89540500	78.38319400	37.98139500
C	52.99459200	77.35731700	38.33067200
C	55.00517800	78.06829000	37.17564500
C	53.21701900	76.04939600	37.88712100
C	55.22012800	76.75749800	36.72763300
C	54.32550900	75.74388100	37.08513000
H	51.53132300	80.02965500	38.00386400
H	54.57635200	80.39981100	38.30502900
H	53.42913700	79.82167300	39.51717000
H	52.12161100	77.57457900	38.94624200
H	55.71826300	78.84926200	36.91907000
H	52.52266000	75.26753100	38.17950900
H	56.08676600	76.53111600	36.11290600
H	54.49232900	74.72488100	36.74850600
H	53.24347200	79.69365500	35.90673100
C	42.32839900	85.23106800	39.72361900
C	44.59015700	83.99615600	43.83102500
C	40.33455300	82.88887500	45.87000800
C	38.05849500	84.22835300	41.79968900
C	43.30942800	85.01861600	40.67515800
C	44.72750200	85.25173800	40.45528900
C	45.36954500	84.90374500	41.62225000
C	44.33882500	84.46013600	42.55314300

C	46.83492200	84.96526700	41.93501100
C	45.30651500	85.77536500	39.17541300
H	44.98643100	86.80916600	38.98524300
C	43.63359800	83.55725900	44.73382500
C	43.92832000	83.09474000	46.08123500
C	42.71114700	82.81285100	46.67405200
C	41.68850300	83.07823700	45.66912800
C	45.28825000	82.99096600	46.69945100
C	42.42651000	82.41151400	48.05013000
C	43.20862100	81.65651700	48.84553800
C	39.34041100	83.13239400	44.93634900
C	37.92584000	82.97614100	45.18751900
C	37.26925300	83.37127500	44.03213700
C	38.30044300	83.75857000	43.08019100
C	37.33135600	82.45134400	46.45882800
C	35.84130300	83.38301800	43.73602900
C	34.82700100	83.49852500	44.61487100
C	39.02629800	84.59953400	40.88384700
C	38.74069200	85.01700000	39.51987200
C	39.95076000	85.27898200	38.92198000
C	40.97331400	85.03246100	39.92467900
C	37.36439400	85.11759700	38.93285100
C	40.23353100	85.72172000	37.51760300
H	40.77460400	86.67647100	37.49402400
N	43.07742800	84.53284800	41.96196800
N	42.26453400	83.52579400	44.48600900
N	39.56545300	83.59297000	43.63697800
N	40.39915600	84.60431500	41.11844300
Fe	41.31738600	84.25678900	42.86728800
H	44.97898600	85.17071200	38.31963500
H	46.39952200	85.76252000	39.18399000
H	41.48926100	82.78783100	48.46127600
H	35.58020800	83.28674200	42.68258400
H	39.31047700	85.85667000	36.94650100
H	40.84914800	84.98895100	36.97980000
H	44.12351900	81.18227600	48.50410100
H	42.91026700	81.45080800	49.86982700
H	34.98320000	83.64420700	45.67769400
H	33.79498600	83.47626800	44.27950600
H	37.39819500	85.46433800	37.89613300
H	36.73545400	85.82181800	39.49313000
H	36.84606000	84.14926000	38.93829800
H	47.43088000	85.15236300	41.03713000
H	47.05633900	85.76593500	42.65412600
H	47.19235400	84.02854600	42.38243600
H	36.39970100	81.91154300	46.25922900
H	38.01109500	81.76225800	46.96975100
H	37.09304900	83.25844200	47.16589000
H	45.66957900	81.96174300	46.67478200
H	45.24712600	83.27836800	47.75665500
H	46.01920700	83.63588700	46.20308500
O	42.33685900	81.08236000	39.77490500
C	42.03787600	81.02701900	38.39709300
C	40.93474200	80.46429800	37.77177200
O	39.94976200	79.75639500	38.48807300
C	38.76672400	80.53623900	38.92902500
C	40.86226100	80.56320400	36.36932500
C	41.87514900	81.20166000	35.64487900
C	43.00102300	81.76693100	36.27830500
C	43.04118200	81.65447600	37.65702300
O	44.03411200	82.14759800	38.52858700
C	43.72906600	81.60683900	39.85308800
H	40.01269100	80.11431500	35.86663900
H	41.80978400	81.25208400	34.56390800
H	43.79549100	82.23529900	35.69926600
H	41.03954700	81.37176800	41.14051100
H	38.41605900	81.16175300	38.10343400
H	38.01218000	79.79153500	39.18515100
H	39.02674800	81.12666700	39.81015500
H	44.40061500	80.77295000	40.07519100
H	43.76205800	82.39932400	40.59345800
H	40.08251300	81.83375600	42.36348300
O	40.28037000	81.12934800	41.71334200
H	51.90941700	84.76321100	37.15556200
H	48.08882500	82.67228300	39.64781600

H	45.63528600	85.17386500	34.76336800
H	37.01015900	76.67132600	45.32285500
H	50.28188700	82.02754900	43.03797800
H	48.98213700	81.58968600	44.17555300
H	50.64106600	81.00578500	44.44847100
H	45.62455100	83.97106700	44.14996600
H	40.02533600	82.52768200	46.84238800
H	37.02589400	84.30986100	41.48321900
H	42.64807300	85.58511100	38.75121700
H	47.08083100	79.52407000	36.63042100
H	40.68485500	77.01834400	37.76621100
H	40.70846300	79.11305300	41.68268800
H	38.86645300	74.48506200	42.80068300
H	52.30996600	81.06458200	34.33903500
H	52.42454000	81.55625900	38.02335100
H	46.22024300	75.88902400	48.17511400
